# Noncanonical Amino Acids in Biocatalysis

**DOI:** 10.1021/acs.chemrev.4c00120

**Published:** 2024-07-03

**Authors:** Zachary Birch-Price, Florence J. Hardy, Thomas M. Lister, Anna R. Kohn, Anthony P. Green

**Affiliations:** Manchester Institute of Biotechnology, School of Chemistry, University of Manchester, Manchester M1 7DN, U.K.

## Abstract

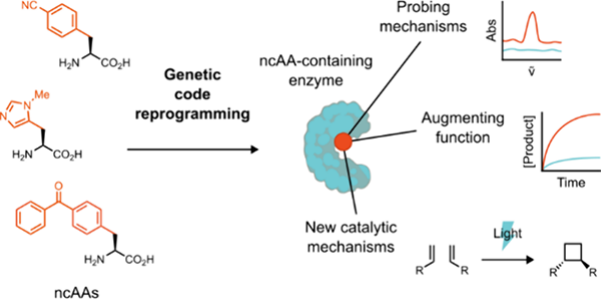

In recent years, powerful genetic code reprogramming methods have emerged that allow new functional components to be embedded into proteins as noncanonical amino acid (ncAA) side chains. In this review, we will illustrate how the availability of an expanded set of amino acid building blocks has opened a wealth of new opportunities in enzymology and biocatalysis research. Genetic code reprogramming has provided new insights into enzyme mechanisms by allowing introduction of new spectroscopic probes and the targeted replacement of individual atoms or functional groups. NcAAs have also been used to develop engineered biocatalysts with improved activity, selectivity, and stability, as well as enzymes with artificial regulatory elements that are responsive to external stimuli. Perhaps most ambitiously, the combination of genetic code reprogramming and laboratory evolution has given rise to new classes of enzymes that use ncAAs as key catalytic elements. With the framework for developing ncAA-containing biocatalysts now firmly established, we are optimistic that genetic code reprogramming will become a progressively more powerful tool in the armory of enzyme designers and engineers in the coming years.

## 1 Introduction

Nature has evolved proteins with a diverse array of structures and functions using a standard set of twenty amino acid building blocks, as defined by the universal genetic code. Enzymes are a subset of proteins that accelerate the biochemical processes needed to sustain life. These biological catalysts use complex networks of active site residues and well-defined substrate binding pockets to process challenging transformations with unrivalled efficiencies and specificities.^1^ In some cases, enzymes recruit additional functional components such as metal ions or organic cofactors to expand upon the limited chemical diversity contained within the standard amino acid side chains.^2^

Over the past decades the fields of biocatalysis and enzymology have benefitted greatly from increasingly sophisticated methods for protein engineering, which have given us unprecedented control over protein sequence, structure and function. For example, site directed mutagenesis has been extensively used to study the roles of individual amino acids in catalysis, advancing in our understanding of enzyme mechanisms.^3^ More extensive engineering of proteins has been enabled by methods such as directed evolution^4,5^ and computational (re)design,^6−8^ leading to the development of biocatalysts with improved stability, selectivity, efficiency and substrate range.^9,10^ These approaches have even allowed access to enzymes with mechanisms and catalytic functions that are unknown in nature.^11,12^

**Figure 1:**
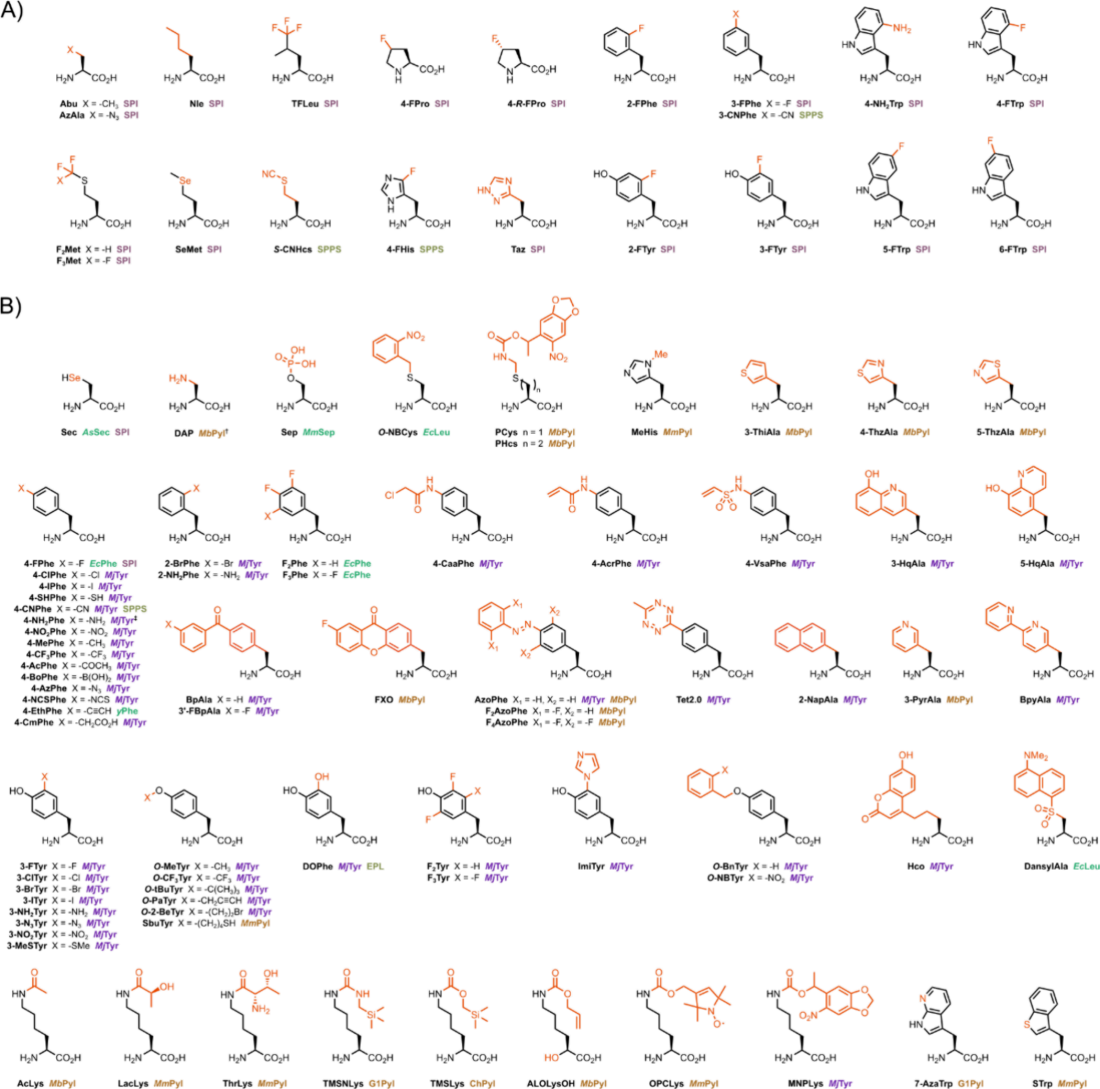
NcAAs discussed in this review. (A) NcAAs incorporated via selective pressure incorporation (SPI), expressed protein ligation (EPL), or solid-phase peptide synthesis (SPPS). (B) NcAAs incorporated by GCE. The orthogonal translation system(s) used to incorporate each ncAA are listed. For several ncAAs, multiple incorporation techniques are discussed in this review, and these are also listed. ^†^DAP is incorporated as a precursor featuring a photocleavable group, which matures to DAP upon irradiation at 365 nm. ^‡^4-NH_2_Phe is incorporated as 4-AzPhe, which is then chemically reduced in situ to form 4-NH_2_Phe.

Although powerful, most enzyme engineering approaches only make use of nature’s standard alphabet of twenty canonical amino acids (cAAs). These standard amino acids are limited in their chemical diversity, which ultimately restricts our control of protein structure and function. To address this limitation, recent years have seen the emergence of powerful genetic code reprogramming methods that allow introduction of new functional elements into proteins as noncanonical amino acid (ncAA) side chains.^13,14^ These methods can be divided into global replacement strategies, where one of the twenty standard amino acids is replaced by a noncanonical structural analogue, or site selective genetic code expansion (GCE) strategies where proteins are synthesized from 21 or more amino acids. To date, these methods have been used to encode hundreds of structurally diverse ncAAs into proteins for diverse applications (Figure 1).^15,16^ Genetic code reprogramming has allowed introduction of new spectroscopic handles^17−19^ and targeted replacement of individual atoms or functional groups,^20,21^ providing new insights into how enzymes operate. These methods have also been used to develop engineered biocatalysts with augmented properties.^22−26^ By combining genetic code reprogramming with directed evolution, it has also been possible to embed new modes of catalysis into proteins that would be difficult to access within the constraints of the genetic code.^27,28^

In this review article, we discuss the different approaches available to incorporate ncAAs into proteins and illustrate how these amino acids have been used for diverse applications in enzyme design, engineering, and characterization.

## 2 Enabling Technologies

### 2.1 Post-Translational Protein Modifications

New functionality can be introduced into proteins post-translationally using a variety of chemical or enzymatic methods.^29,30^ The thiol group of Cys is commonly targeted for protein modification, usually *via* alkylation or disulphide bond exchange reactions, due to its high nucleophilicity and relative scarcity in proteins.^31^ Although achieving specific and selective modification is often challenging, post-translational protein functionalization is highly versatile and has allowed incorporation of diverse nonproteogenic elements.^32−35^ Covalent modification of Cys residues to introduce new catalytic elements has provided artificial enzymes for a range of chemical transformations. For example, flavin analogues have been installed into the active site pocket of the cysteine protease, papain, to generate biocatalysts with oxidoreductase activity.^36−38^ Ruthenium and rhodium complexes have also been introduced into protein scaffolds *via* Cys functionalization, to provide enzymes for ring closing metathesis^39^ and hydroformylations,^40−42^ respectively.

### 2.2 Solid-Phase Peptide Synthesis

Functionalized peptides and proteins can be produced using solid-phase peptide synthesis (SPPS), involving sequential coupling of protected amino acids on a solid support.^43^ This approach is technically challenging and time-consuming but offers flexibility, allowing synthesis of user-defined peptide sequences containing a variety of canonical and artificial building blocks, including those that are toxic to cells or poorly compatible with the cellular translation machinery (e.g., *D*-amino acids).^44−47^ Peptides synthesized using SPPS are typically restricted to sequences of less than 100 residues; however larger proteins can be constructed by combining SPPS with a variety of ligation strategies including native chemical ligation (NCL) and expressed protein ligation (EPL).^48−54^ NCL offers greater flexibility with respect to the number and distribution of ncAAs that can be introduced into the polypeptide chain; however, producing large proteins is both costly and technically challenging. In contrast, EPL allows for more facile production of large modified proteins but is typically limited to the introduction of ncAA-containing synthetic sequences at the *C*-terminus. These synthetic or semisynthetic methods have been used to create polymer-modified glycoprotein mimetics,^55−58^ proteins with modified backbones^59−61^ and defined patterns of posttranslational modifications,^62−64^ split-proteins,^65^ proteins containing fluorescent probes^66−68^ and proteins with altered catalytic properties.^47,69,70^

### 2.3 Selective Pressure Incorporation

**Figure 2:**
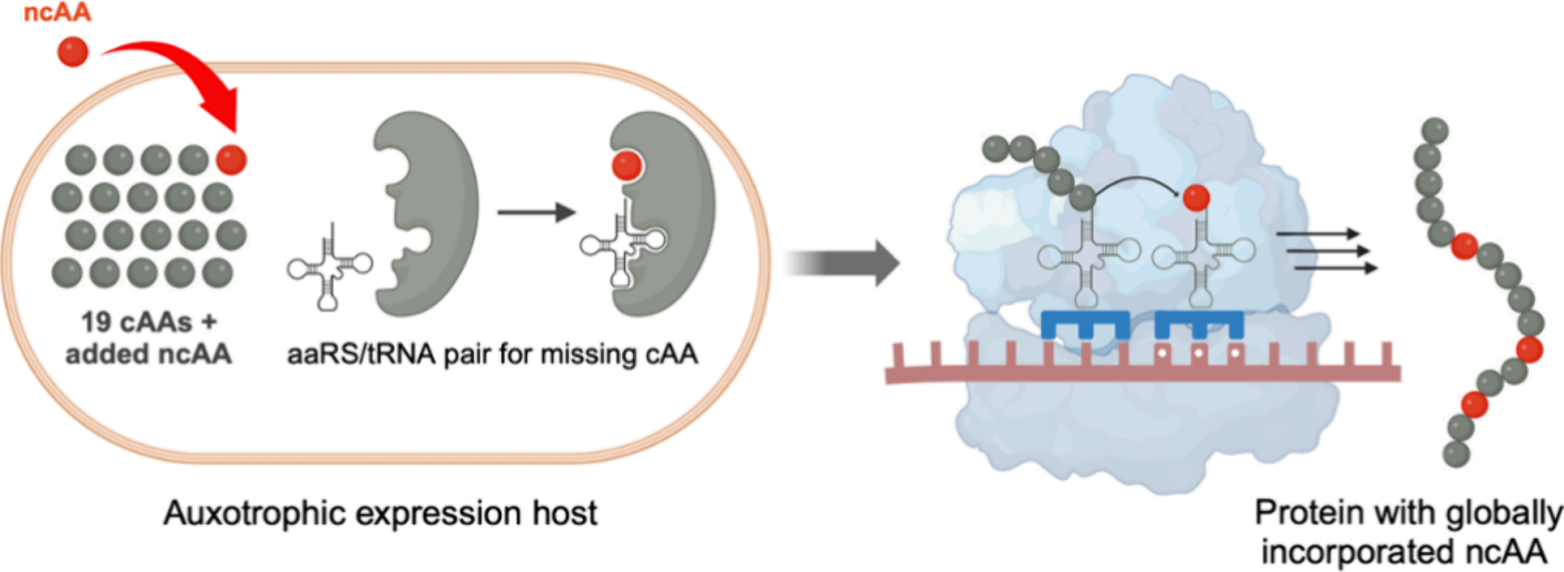
SPI of ncAAs. SPI employs an auxotrophic expression system to globally replace a target canonical amino acid (cAA) with a close structural analogue. An endogenous aaRS loads its cognate tRNA with the ncAA which is incorporated into proteins. Created with BioRender.com.

Selective pressure incorporation (SPI) exploits the promiscuity of native aminoacyl tRNA synthetases (aaRSs) to aminoacylate a canonical tRNA with a close structural analogue of its cognate cAA, leading to sense codon reassignment (Figure 2). This method results in global replacement of one of the twenty standard amino acids by a ncAA. SPI relies on the use of natural or engineered auxotrophic expression strains that are deficient in a target amino acid.^71−73^ A seminal study by Cowie and Cohen reported the quantitative substitution of Met for selenomethionine (SeMet) in proteins produced by the Met auxotroph *Escherichia coli* (*E. coli*) ML304d.^74^ In this case, the auxotrophic strain displayed normal exponential growth when Met was swapped for SeMet in the growth media, installing SeMet throughout every protein in the bacterial proteome. Other auxotrophic strains have also been developed, enabling the global replacement of Trp with 4-fluorotryptophan^75^ (4-FTrp) and valine with α-aminobutyric acid.^76^ However, for many ncAAs, global incorporation throughout the host proteome is detrimental to cell growth. To address this challenge, auxotrophic strains are often cultured in defined media containing a low concentration of the cAA, which becomes depleted during cell growth. The ncAA is then supplemented into the media at an appropriate growth state when protein expression is induced. Alternatively, the cells can be harvested prior to induction and resuspended in fresh media containing ncAA but lacking the cAA. To further expand the range of ncAAs that can be incorporated using SPI, the substrate promiscuity of canonical aaRSs has been extended using protein engineering.^76−79^

SPI has enabled the successful incorporation of a wide range of ncAAs into recombinant proteins for diverse applications (Figure 1A). For example, proteins containing heavy atom analogues of cAAs are routinely used in protein X-ray crystallography to aid experimental phasing.^80−84^ Isosteric amino acid analogues have been used to investigate protein folding, stability and activity.^85−90^ SPI has also been used to install spectroscopic probes^91−95^ and biorthogonal handles for protein conjugations.^77,79,96−101^ As detailed in the sections below, this approach has also been widely used to improve enzyme stability and activity.

### 2.4 Genetic Code Expansion

GCE enables the site selective incorporation of ncAAs *in vivo* or *in vitro*, in response to a reassigned codon. In this way proteins containing 21 or more amino acids can be biosynthesized.

#### 2.4.1 *In Vitro* Genetic Code Expansion

**Figure 3:**
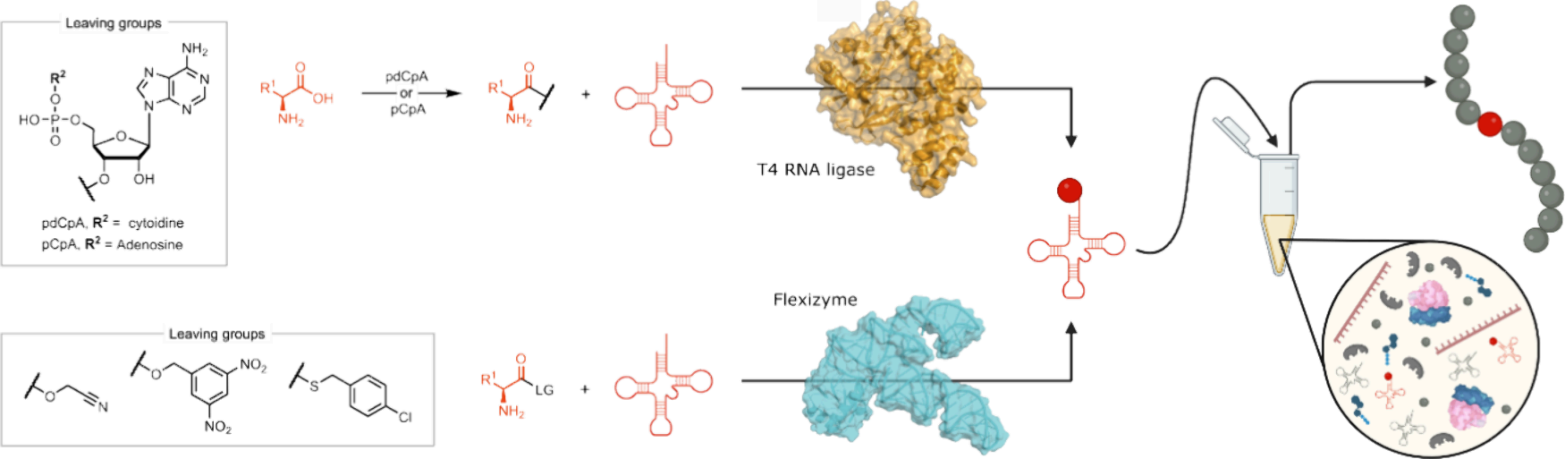
Strategies for the generation of ncAA-loaded tRNAs employ either chemoenzymatic methods (top left, PDB: 2C5U(102)) or Flexizymes (bottom left, PDB: 3CUN(103)). These ncAA-tRNAs can then be incorporated into a polypeptide chain using cell-free expression (CFE) systems (right). Created with BioRender.com.

Peptides and proteins containing ncAAs have been ribosomally synthesized *in vitro* using cell free extracts (e.g., *E. coli* lysates) or reconstituted protein synthesis systems (e.g., PURExpress).^104−106^ Here, tRNAs precharged with the target ncAA are supplemented into an *in vitro* expression system and decoded by the ribosome in response to a repurposed codon (Figure 3). Early work on *in vitro* genetic code reprogramming focused on chemically modifying aminoacyl-tRNAs.^107,108^ For example, conversion of a Phe-loaded tRNA to the corresponding α-hydroxy acid enabled production of proteins with polyester backbones.^108^

The development of chemo-enzymatic strategies for synthesizing aminoacyl-tRNAs has provided a more general approach that has expanded the range of ncAAs that can be incorporated into proteins. Here, an *N*-protected amino acid is first chemically coupled to di(adenosine 5′-)diphosphate or a 5′-phospho-2′-deoxyribocytidylriboadenosine (pdCpA) dinucleotide at the 3′-hydroxyl group. The resulting aminoacylated nucleotide fragment is then ligated to a truncated tRNA using T4 RNA ligase and deprotected to yield the final aminoacyl tRNA.^104,109^ Alternatively, aminoacylation ribozymes, which are RNA-based catalysts, can be used to directly charge tRNAs with ncAAs.^110^ In particular, Flexizymes have proven to be valuable catalysts for mediating transacylation of activated amino acid esters onto the 3′-hydroxyl group of tRNAs.^111,112^ By mixing Flexizymes with tRNA and the respective amino acid ester, nearly any tRNA can be loaded with almost any desired ncAA (Figure 3).^105^ To date, efficient and precise ribosomal translation of proteins containing diverse ncAAs has been achieved.^113^ Combining Flexizyme genetic code reprogramming technology with mRNA display has proven especially valuable for discovery of macrocyclic peptide binders in drug development.^114^ Although powerful, one drawback of these *in vitro* methods is that they cannot be easily scaled due to the requirement to supply super stoichiometric quantities of aminoacyl-tRNAs as well as the high costs associated with reconstituted *in vitro* translation systems. These challenges have so far limited the use of these technologies in biocatalysis due to the relatively large quantities of protein required for most applications.

#### 2.4.2 *In Vivo* Genetic Code Expansion

**Figure 4:**
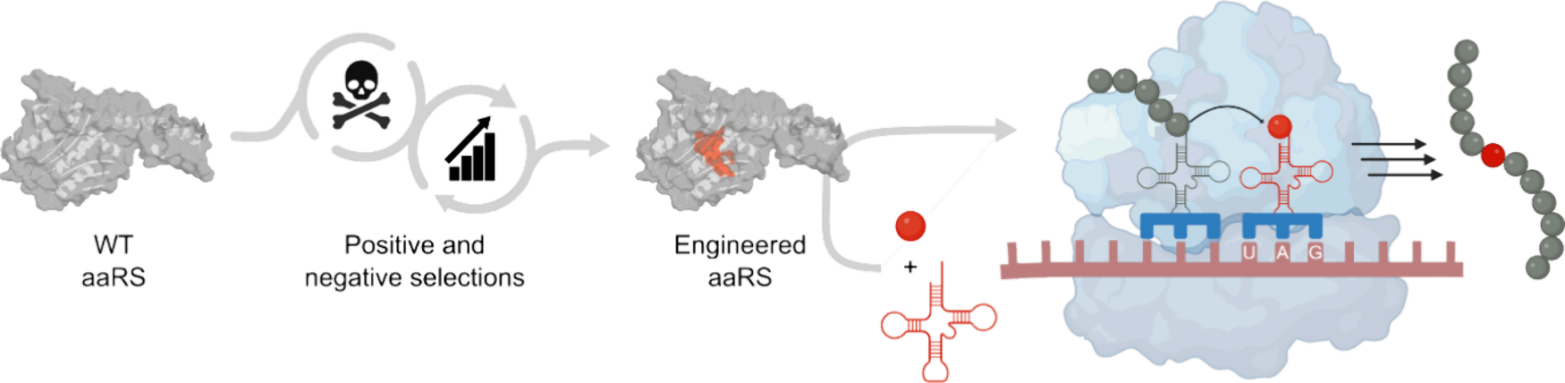
Positive and negative selection processes can be used to engineer orthogonal aaRS-tRNA pairs to improve incorporation efficiency and/or specificity. The engineered aaRS catalyzes an aminoacylation reaction between its cognate tRNA and ncAA, with the ncAA added to the growing polypeptide chain during translation in response to a repurposed codon (e.g., the amber stop codon, UAG). Created with BioRender.com.

*In vivo* genetic expansion methods provide a more scalable approach to the synthesis of ncAA-containing proteins. These methods rely on an orthogonal aminoacyl-tRNA synthetase–tRNA pair to direct the incorporation of an ncAA in response to a repurposed codon introduced into the gene of interest (Figure 4).^115^ Of the codons available, the amber codon (UAG) is most commonly used, although reassigned sense codons and quadruplet codons have also been exploited.^116,117^ The aaRS/tRNA pair used for ncAA incorporation must be orthogonal in the context of all endogenous aaRS/tRNA pairs within the host organism. This orthogonality is most commonly achieved by importing a heterologous aaRS/tRNA pair from a different domain of life.^118^ If necessary, orthogonality can be improved through aaRS and/or tRNA engineering. Finally, the substrate specificity of the heterologous aaRS is engineered so that it uniquely recognizes the ncAA of interest.^118^ Although several methods have been reported to engineer aaRSs,^119−123^ this is most often achieved by subjecting large active site libraries to a two-stage selection protocol that links cell viability to aaRS activity and specificity.^124^ A positive selection step, in which cell survival is dependent upon suppression of an amber codon introduced into the chloramphenicol acetyl transferase (CAT) gene, selects for aaRSs that aminoacylate the suppressor tRNA with the target amino acid. A subsequent negative selection step then removes aaRSs that incorporate cAAs. The positively selected clones are transformed into cells containing a gene encoding a toxic barnase protein with amber mutations at permissive sites. The cells are then grown in the absence of the ncAA, and the presence of aaRS variants that are able to utilize cAAs will result in cell death. The activity and specificity of surviving clones can be quantified using a fluorescence screening assay which relies on suppression of an amber codon introduced into green fluorescent protein (GFP).^125^

Using this approach, hundreds of structurally and functionally diverse ncAAs can now be incorporated into proteins (Figure 1B).^126,127^ For example, a large number of aromatic ncAAs have been incorporated into proteins produced in *E. coli* using engineered TyrRS-tRNA^Tyr^ pairs derived from *Methanocaldococcus jannaschii* (*Mj*TyrRS-tRNA^Tyr^).^128^ The structural diversity of encodable ncAAs has been further expanded following the discovery of PylRS-tRNA^Pyl^ pairs, which naturally suppress amber codons to incorporate ‘the 22nd amino acid’ pyrrolysine (Pyl, O) in methanogenic archaea.^127,129,130^ Due to a high degree of active site plasticity and orthogonality in *E. coli*, yeast and mammalian cells, PylRS systems have emerged as the most versatile platform for GCE *in vivo*.^127,131^

## 3 Probing Enzyme Mechanisms with Noncanonical Amino Acids

Site directed mutagenesis has proven to be an invaluable tool in enzymology, providing a versatile strategy for identifying catalytically important residues. However, standard mutations of active site residues can sometimes lead to unintended structural changes that can complicate interpretation of structure–activity relationships. Furthermore, mutation of key catalytic residues often dramatically reduces activity, which provides important but limited mechanistic insights. The incorporation of ncAAs can open new avenues to study complex biochemical mechanisms by allowing more nuanced perturbations to enzyme structure and function.^14,20,22,132^ Such modifications can be used to introduce spectroscopic probes, enable covalent trapping of intermediates and fine-tune active site environments by replacement of specific atoms or functional groups.

### 3.1 Incorporation of Spectroscopic Handles and Biophysical Probes

Strategic incorporation of ncAAs with distinct spectroscopic and biophysical properties has been used to study enzyme function and structure through a range of techniques, including NMR,^133,134^ EPR,^135^ IR,^136^ and X-ray crystallography.^137^ Additionally, ncAAs with fluorescent sidechains such as dansyl-Ala^138^ or (7-hydroxycoumarin-4-yl)ethylglycine (Hco),^20^ or with biorthogonal handles to which fluorescent molecules can be conjugated,^139−141^ have been introduced into peptides and proteins to monitor folding and conformational changes,^142−144^ for example in superoxide dismutase,^138^ myoglobin (Mb),^145^ deubiquitinases,^146^ and T4 lysozyme.^147^

#### 3.1.1 Noncanonical Amino Acids to Facilitate Protein Structure Determination

Global incorporation of SeMet through SPI has enabled great advances in protein structure determination by X-ray crystallography, through additional anomalous signals derived from the presence of heavy atoms within the crystal lattice.^148^ Genetic incorporation of 4-iodophenylalanine (4-IPhe)^137^ or 3-iodotyrosine (3-ITyr)^149^ gives a site selective way to introduce an alternative heavy atom to facilitate such single-wavelength anomalous diffraction (SAD) experiments and allow structure determination with less diffraction data. Additionally, a metal-chelating ncAA, (8-hydroxyquinolin-3-yl)alanine (3-HqAla), provided a binding site for Zn(II) within the crystal lattice of *O*-acetylserine sulfhydrylase, which enabled SAD phasing to determine protein structure.^150^

Recently, X-ray free-electron lasers (XFELs) have allowed structural characterization of transient reaction intermediates using time-resolved serial femtosecond crystallography. Light is a convenient trigger for time-resolved experiments, and several natural photoenzymes have been studied with XFELs.^151−157^ Although not yet applied to catalysis, GCE can be used to introduce artificial phototriggers into proteins, such as photocaged AAs or triplet sensitizers. The Wang laboratory used GCE to encode (*S*)-2-amino-3-(7-fluoro-9-oxo-9H-xanthen-2-yl)propanoic acid (FXO), an ncAA that has a strongly absorbing xanthone side chain that undergoes a photocrosslinking when placed in the hydrophobic pocket of a human liver fatty acid binding protein.^158^ Time-resolved crystallography experiments were used to monitor conformational changes in the xanthone side chain between 10 and 300 ns after irradiation. In principle, this approach could be extendable to studying mechanisms of designed enzymes with triplet photosensitizers (*vide infra*).^159^

#### 3.1.2 Nuclear Magnetic Resonance Studies with Noncanonical Amino Acids

Protein nuclear magnetic resonance (NMR) spectroscopy is a widely used technique that detects signals from spin-active nuclei, and can provide information about reaction kinetics or conformational changes upon ligand binding. To study proteins *via* NMR, proteins can be expressed in ^15^N- and/or ^13^C-enriched media, resulting in uniform labeling of the entire protein. NcAAs can be used to simplify the interpretation of protein NMR data by enabling site-specific isotope labeling or by introducing NMR-active nuclei into proteins, which can be used as sensitive probes that can report on changes to the local conformation and/or electronic environment upon, for example, ligand binding.^160,161^ This approach can prove especially valuable for analysis of large proteins, where interpretation of spectra can be complicated. NcAA incorporation has been widely used to selectively install isotopic labels and new spin active nuclei to facilitate protein NMR analysis. In this section we present illustrative examples of where ncAAs have been used to study enzymes through NMR. The reader is directed to excellent reviews for a more comprehensive overview.^133,162^

One early report from 1968 studied staphylococcal nuclease by replacing 14 of the amino acids with their deuterated analogues.^160^ The simplified NMR spectra facilitated analysis of the effects of metal ion and inhibitor binding. Site-specific isotopic enrichment was first achieved using a chemically aminoacylated suppressor tRNA to introduce a single ^13^C-labeled Ala82 into T4 lysozyme in response to a TAG codon.^134^ This enabled isolation of the signal from Ala82 in ^13^C NMR spectra and allowed for observation of changes in shift dispersion upon denaturation. More recently, an engineered tRNA^Pyl^/G1PylRS pair was used to encode ^15^N-/^13^C-labeled 7-azatryptophan (7-AzaTrp) into a protease from the Zika virus, establishing a method for achieving site-specific peak assignments in Heteronuclear Single Quantum Coherence (HSQC) spectra.^163^

Three different ncAAs (*O*-trifluoromethoxytyrosine (*O*-CF_3_Tyr), ^13^C-/^15^N-labeled *O*-MeTyr, and ^15^N-labeled *O*-nitrobenzyltyrosine (*O*-NBTyr)) were introduced into eleven positions of the active site of the thioesterase domain of human fatty acid synthase. The differential chemical shifts in HSQC and ^19^F NMR spectra in response to ligand binding confirmed the ligand binding site, and gave information on protein motion.^164^ Similarly, ^13^C-*O*-MeTyr was used to provide evidence for the existence of multiple, ligand-dependent conformational states of the cytochrome P450 CYP119.^165^

NcAAs can also be used to introduce spin-active nuclei that do not naturally occur in proteins. Due to the intrinsic NMR sensitivity of ^19^F and the lack of fluorine-containing cAAs, ncAAs such as 5-fluorotryptophan (5-FTrp), 6-FTrp, 3-FTyr, and 4-FPhe have provided useful spectroscopic handles for studying protein structure and function.^166−175^

SPI was used to introduce trifluoromethionine (F_3_Met) and difluoromethionine (F_2_Met) at positions 1, 14 and 107 of lysozyme from bacteriophage λ (LaL).^174,176^ Of three sites of ncAA incorporation, the F_2_Met14 residue in the core of the protein showed the largest chemical shift difference between the two fluorine signals, likely due to restricted side chain movement. Upon binding of an oligosaccharide inhibitor, the two F_2_Met14 signals diverge even further, suggesting a conformational change to the enzyme in a region closer to one of the two fluorine atoms.^177^ In a similar manner, replacement of Met with F_3_Met in DNA polymerase I from *Thermus aquativus* (KlenTaq) enabled ^19^F-NMR studies to study enzyme dynamics.^178^ Individual ^19^F resonances could be resolved and the spectra could be used to distinguish between the free enzyme, the binary complex in the presence of a DNA primer, and the ternary complex with a ddNTP bound. This method has also been used to study conformational changes upon ligand binding at the dimeric interface of nitroreductase and histidinol dehydrogenase.^168^ The ^19^F spectra of nitroreductase containing 4-CF_3_Phe at position 124 showed a single peak corresponding to the ncAA, which shifted upfield upon binding of an inhibitor due to conformational changes.

Fluorine NMR signals show scalar ^19^F-^19^F coupling when in proximity. Orton et al. demonstrated these effects are observable within the hydrophobic core of the *E. coli* protein cis–trans prolyl-isomerase B by positioning multiple fluorinated ncAAs.^179^ Genetic incorporation of two 4-CF_3_Phe or *O*-CF_3_Tyr residues gave observable ^19^F-^19^F Total Correlation Spectroscopy (TOCSY) and Double Quantum Filtered Correlation Spectroscopy (DQF-COSY) cross peaks with high sensitivity. These studies suggest that through space ^19^F-^19^F couplings between fluorine substituted ncAAs can offer a sensitive tool for studying protein structure and function.

NcAAs can also be used to introduce functional groups that occupy distinct chemical shift regions within ^1^H NMR spectra. For example, Ekanayake et al. developed an engineered aminoacyl-tRNA synthetase to incorporate *Ν*_ε_-(((trimethylsilyl)methyl)-carbamoyl)lysine (TMSNLys) for study of the dimeric SARS-CoV-2 main protease.^133^^1^H NMR signals from the trimethylsilyl (TMS) group of TMSNLys and a second ncAA, *N*_ε_-(((trimethylsilyl)-methoxy)carbonyl)lysine (TMSLys) appear at ∼ 0 ppm, a clear spectral region with few features from aqueous buffer or protein environments. Distinct peak shifts were observed upon binding of two ligands, a cyclic peptide inhibitor and Calpeptin. Interestingly, the expected perturbations were not observed with one putative allosteric ligand, pelitinib, suggesting that this ligand does not bind in the region previously proposed in the literature.

#### 3.1.3 Incorporation of Noncanonical Amino Acids for Electron Paramagnetic Resonance Spectroscopy

Electron paramagnetic resonance (EPR) spectroscopy is a powerful biophysical technique for characterizing species with unpaired electrons, such as transition metals complexes or organic radicals.^180^ Site-selective spin labeling has been achieved by reacting Cys residues with nitroxide-labeled reagents, although this method relies on the introduction and/or removal of cysteine residues that might have functional significance.^181^ As an alternative, GCE can be used to directly encode paramagnetic moieties as amino acid side chains^135^ or ncAAs with bioorthogonal handles to which spin labels can be efficiently conjugated.^182−185^

Direct genetic incorporation of a spin-labeled amino acid ((*S*)-2-amino-6-(1-oxy-2,2,5,5-tetramethylpyrroline-3-carboxamido)hexanoic acid, OPCLys) was achieved using an engineered tRNA^Pyl^/PylRS pair.^135^ Purified GFP with the spin-active ncAA at position 39 gives a nitroxide-characteristic EPR signal with a spectral shape indicative for low N–O• mobility, in contrast to that of the free amino acid that shows a spectrum characteristic to that of a fast-tumbling small molecule. Interestingly, quantification of this signal reveals that the intensity is approximately 50% of that expected, likely due to some instability of nitroxide radicals *in vivo*.

A higher degree of spin-labeling is achievable through post-translational derivatization of ncAAs. Genetic incorporation of 4-acetylphenylalanine (4-AcPhe) has been used to introduce nitroxide spin labels into proteins by acid-catalyzed derivatization of the ncAA side chain with a hydroxylamine nitroxide, first in the model protein T4 lysozyme.^186^ This approach has been extended to include alternative ncAA-spin probe combinations. By incorporating ncAAs with either azide or alkyne functional groups, Cu(I)-catalyzed azide-alkyne cycloadditions (CuAACs) or strain-promoted azide-alkyne cycloadditions (SPAACs) can be used to introduce spin labels into proteins.^183,187−191^ For example, Gd(III) moieties were conjugated to two sites in the Zika virus NS2B-NS3 protease using 4-azidophenylalanine (4-AzPhe).^192^ Double electron–electron resonance (DEER) measurements in the presence and absence of a known inhibitor for this protease revealed similar distance distributions, suggesting the commonly used linked construct of this protease remains in the closed conformation, irrespective of ligand binding.

#### 3.1.4 Noncanonical Amino Acids for Protein Infrared Spectroscopy

Infrared spectroscopy (IR) is a well-established method of study protein structure and function.^193,194^ The IR spectra of proteins have a transparent window between 1800 and 2500 cm^–1^, which can be exploited by incorporating CD (carbon-deuterium), CN,^195−199^ SCN,^200^ N_3_,^201−204^ and NO_2_^205^ bonds into proteins to introduce distinct spectroscopic features. In this way, deuterated Met has been used as a vibrational probe to study a range of proteins including myeloperoxidase,^206^ dihydrofolate reductase,^207^ and plastocyanin.^208^

The addition of ncAAs containing cyano-^19^ and nitro-^205^ groups to the genetic code affords a method for site specific introduction of vibrational probes,^209^ that are sensitive to hydrogen bonding, packing interactions, and protein conformation.^210^ The Boxer laboratory introduced 4-cyanophenylalanine (4-CNPhe), 3-cyanophenylalanine (3-CNPhe), and *S*-cyanohomocysteine (*S*-CNHcs) into the active site of Ribonuclease S (RNaseS) through SPPS of peptide fragments (residues 1–20) that, when combined with a recombinantly expressed protein fragment (residues 21–124), gave active RNase S variants with no significant changes to enzyme structure or activity.^136^ Vibrational Stark Effect (VSE) experiments using each ncAA were used to calibrate the sensitivity of the nitrile stretch to external electric fields, allowing a quantitative interpretation of the frequency shifts, providing a measure of the local electrostatic fields (see section 3.3.2). An expansion of this method used ^13^C-labeled 4-CNPhe to assess solvatochromic effects and account for hydrogen bonding to the aromatic nitriles, allowing an estimate of the average total electrostatic field within an enzyme active site.^211^

Genetically encoded 3-azido-Tyr (3-N_3_Tyr) served as a 2-dimensional IR probe in the study of the metalloenzyme DddK, an iron-dependent enzyme that catalyzes the conversion of dimethylsulfoniopropionate to dimethylsulfide.^212^ Standard substitutions of Tyr64 abolished catalytic function, however substitution to 3-N_3_Tyr was well-tolerated and allowed measurement of fs–ps time scale water dynamics. The addition of even low concentrations of the denaturation reagent guanidinium hydrochloride was shown to decrease water confinement in the active site and substantially reduce catalytic activity without affecting the wider protein structure. The authors proposed that Tyr64 is activated as a catalytic base through deprotonation by a neighboring ordered water molecule, and disruption of this process by addition of the denaturation reagent causes loss of catalytic activity.

### 3.2 Covalent Trapping of Reactive Intermediates and Transient Complexes

NcAAs have been used to covalently trap close analogues of catalytic intermediates and catalytically relevant protein–protein complexes to facilitate their characterization. This approach has provided new structural insights into transient reaction intermediates and can be used to interrogate biosynthetic pathways *in vivo*.

#### 3.2.1 Capturing Acyl-Enzyme Intermediates

**Figure 5:**
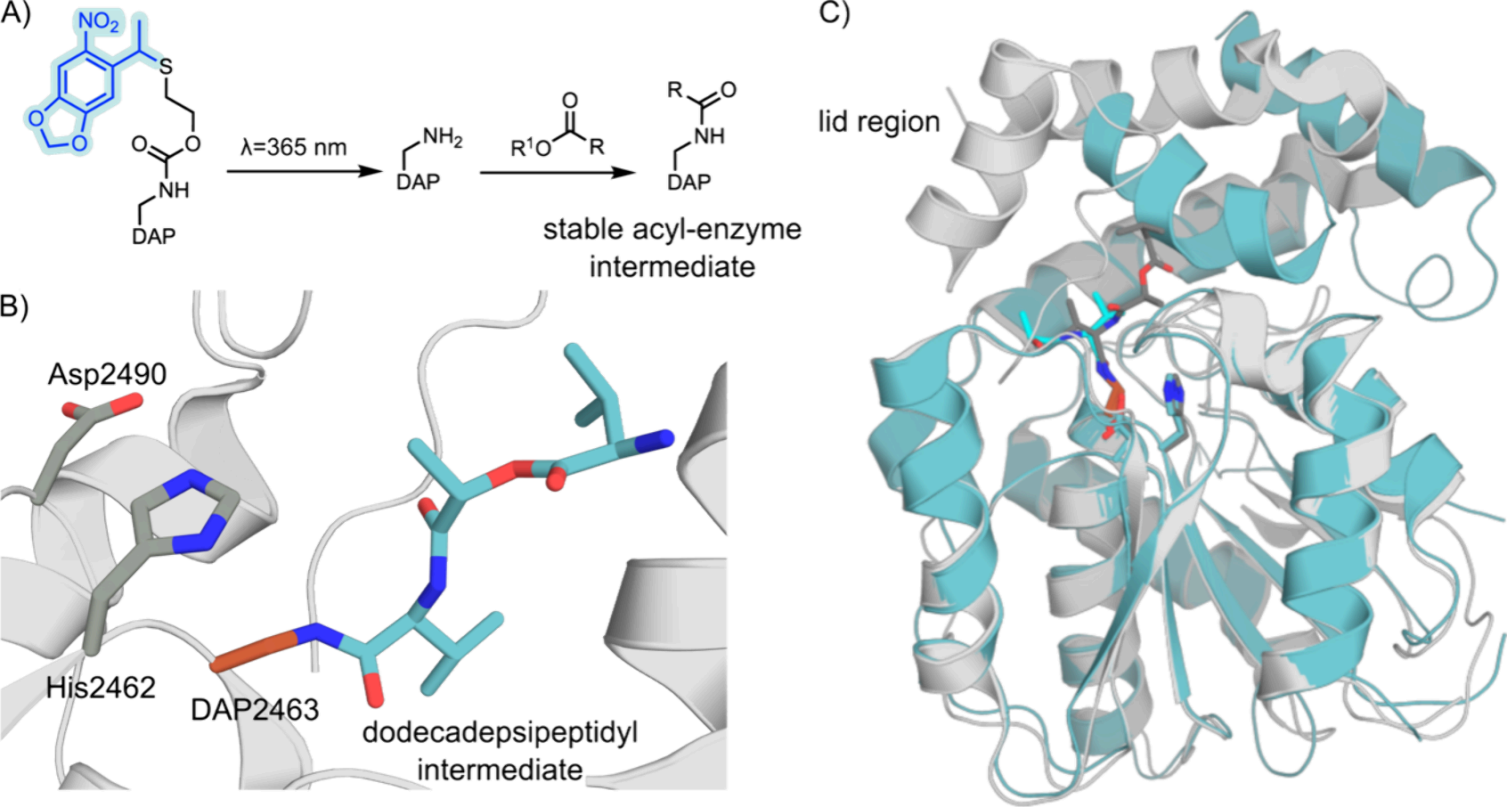
DAP incorporation into Valinomycin synthetase. (A) Genetically encoded (2*S*)-2-amino-3-([(2-[1-(6-nitrobenzo[*d*][1,3]dioxol-5-yl)ethyl]thio)ethoxy)carbonyl] ncAA is photodeprotected by irradiation at 365 nm to give DAP, which forms stable acyl-enzyme intermediates with an amide bond that is resistant to hydrolysis. (B) The active site of Valinomycin synthetase (protein shown as a gray cartoon, PDB: 6ECE(213)) with a noncanonical DAP nucleophile in position 2463 (atom-colored sticks, brown carbons) bound to a dodecadepsipeptide substrate (atom colored sticks, blue carbons). (C) Large structural differences are observed in the lid region of Valinomycin synthetase when bound to a dodecadepsipeptidyl intermediate (gray cartoon, PDB: 6ECE(213)) in comparison to a tetradepsipeptidyl intermediate (blue cartoon, PDB: 6ECD(213)).

Many enzymes such as Ser hydrolases, Cys proteases, and ubiquitinases proceed *via* the formation of transient acyl-enzyme (ester or thioester) intermediates. Replacement of the catalytic serine/cysteine residue with 2,3-diaminopropionic acid (DAP) gave a powerful new method of trapping these intermediates with nonhydrolyzable amide bonds (Figure 5A).^213^ The structural similarity between DAP and Cys/Ser complicates its direct incorporation through cellular translation. Instead, the Chin lab developed an engineered tRNA^Pyl^/PylRS pair to incorporate a photocaged derivative of DAP that can be deprotected post-translation upon irradiation with light. This approach was used for structural analysis of valinomycin synthetase (Figure 5B,C), a member of the nonribosomal peptide synthetase family. Structural characterization of a covalently linked substrate-enzyme complex by X-ray crystallography revealed a large conformational change to a ‘lid’ region upon substrate acyl-enzyme complex formation.

Subsequent work from the Chin laboratory described the use of DAP incorporation to create ‘substrate traps’ in mammalian cells.^214^ Proteases and other hydrolases equipped with DAP nucleophiles were purified from live cells with substrate fragments bound *via* stable amide bonds, thus allowing the annotation of proteins of previously unknown function.

#### 3.2.2 Photo-Cross-Linking to Map Protein–Protein Interactions

Benzophenones are powerful photocross-linking agents that have been widely used as photophysical probes.^215^ The addition of 4-benzoylphenylalanine (BpAla) to the genetic code using an engineered tRNA^Tyr^/*Mj*TyrRS pair allowed introduction of this functional motif into proteins with unparalleled site selectivity.^216^ This approach has enabled mapping of transient protein–protein interactions through covalent photocrosslinking,^217^ including complexes between glutathione *S*-transferase (GST) dimers,^218^ SecA Atpase and the SecYEG translocon,^219^ the transcriptional activator Gal4 and the inhibitor protein Gal80,^220^ the large subunit of rubisco and its chaperone RbcX_2_,^221^ acyl carrier proteins and ketosynthases,^222,223^ and vaccinia H1-related (VHR) phosphatase dimers.^224^ In one notable example, this approach was used to identify a new preinitiation complex in the mechanism of a human mitochondrial RNA polymerase (mtRNAP).^225^ Mammalian transcription apparatus contains mtRNAP, a transcription initiation factor-like protein TFB2M, and a major nucleoid protein TFAM. Upon incorporation of BpAla into the *C*-terminal region of TFAM, photoinduced cross-linking to mtRNAP was observed, dependent on DNA-binding, independent of the major nucleoid protein TFB2M. These data suggest the mtRNAP mechanism involves formation of an initial complex involving mtRNAP, TFAM and promoter DNA.

### 3.3 Modulating Noncovalent Interactions and Intermediate Lifetimes

GCE allows for selective and targeted substitution of individual functional groups or atoms within an enzyme active site. These molecular edits can then be correlated with changes in catalytic activity and reaction mechanism, to build detailed structure–activity relationships that are not possible to achieve with standard mutagenesis methods.^226,227^ In this section, we illustrate examples of how ncAAs have be used to tune the p*K*_a_ and/or redox potential of selected residues,^228^ modulate hydrogen bonding networks^229^ or other noncovalent interactions,^230^ and to perturb the lifetimes of reactive intermediates.^231^

#### 3.3.1 Tuning the p*K*_a_ and/or Reduction Potential of Key Residues

Using ncAAs to modulate the p*K*_a_ and/or reduction potential of key functional residues has provided new insights into the mechanisms of a diverse set of enzymes.

Variants of RNase A with the catalytic His, His12 and His119, replaced by 4-fluorohistidine (4-FHis) were synthesized by stepwise peptide ligation, to give single and double mutants of the enzyme.^48^ The pH-rate profiles of each variant for the cleavage of uridyl-3′,5′-adenosine were different to that of the wild type (WT) due to the lower p*K*_a_ of 4-FHis cf. His. RNase A His12(4-FHis) has a broader pH profile and is able to effect catalysis at ∼ 2 pH units lower than the WT, consistent with its proposed role as a catalytic base. In contrast, replacement of His119, which is thought to serve as a catalytic acid, reduced activity substantially, likely due to the more acidic 4-FHis existing in an inactive nonprotonated state.

Replacement of an active site His of a porcine pancreatic phospholipase A_2_ (PLA) by 1,2,4-triazole-3-alanine (Taz) using SPI yields a catalytically active enzyme with an altered pH-rate profile to the WT.^232^ WT PLA has a pH optimum of 6, with no activity at pH 3. However, at both pH 6 and pH 3 PLA His48Taz is active, at approximately a 5-fold lower rate in comparison to the WT maximum. The mechanism of phospholipase relies on water activation by basic His48. The lower p*K*_a_ of Taz compared with His ensures that it remains in its active, nonprotonated state at lower pH and is able to activate the nucleophilic water. In contrast, replacement of the only remaining His in a His31Asn/His137Asn double mutant of LaL by Taz had limited effect on the pH-rate profile.^233^

To tune the p*K*_a_ of an active site tyrosine in the DNA polymerase KlenTaq, 2,3,5-trifluorotyrosine (2,3,5-F_3_Tyr) was introduced at position 671 using GCE.^234^ Structural analysis of KlenTaq in complex with a DNA template containing an abasic site shows Tyr671 occupies the position of the missing base in the DNA template, forming a hydrogen bond to the N3 of purine bases to guide incorporation of dATP and dGTP.^235^ Replacement of Tyr671 with 2,3,5-F_3_Tyr671 using GCE dramatically reduced activity toward single nucleotide incorporations at abasic sites, showing how even subtle perturbations to hydrogen bonding interactions can impact enzyme activity.

**Figure 6:**
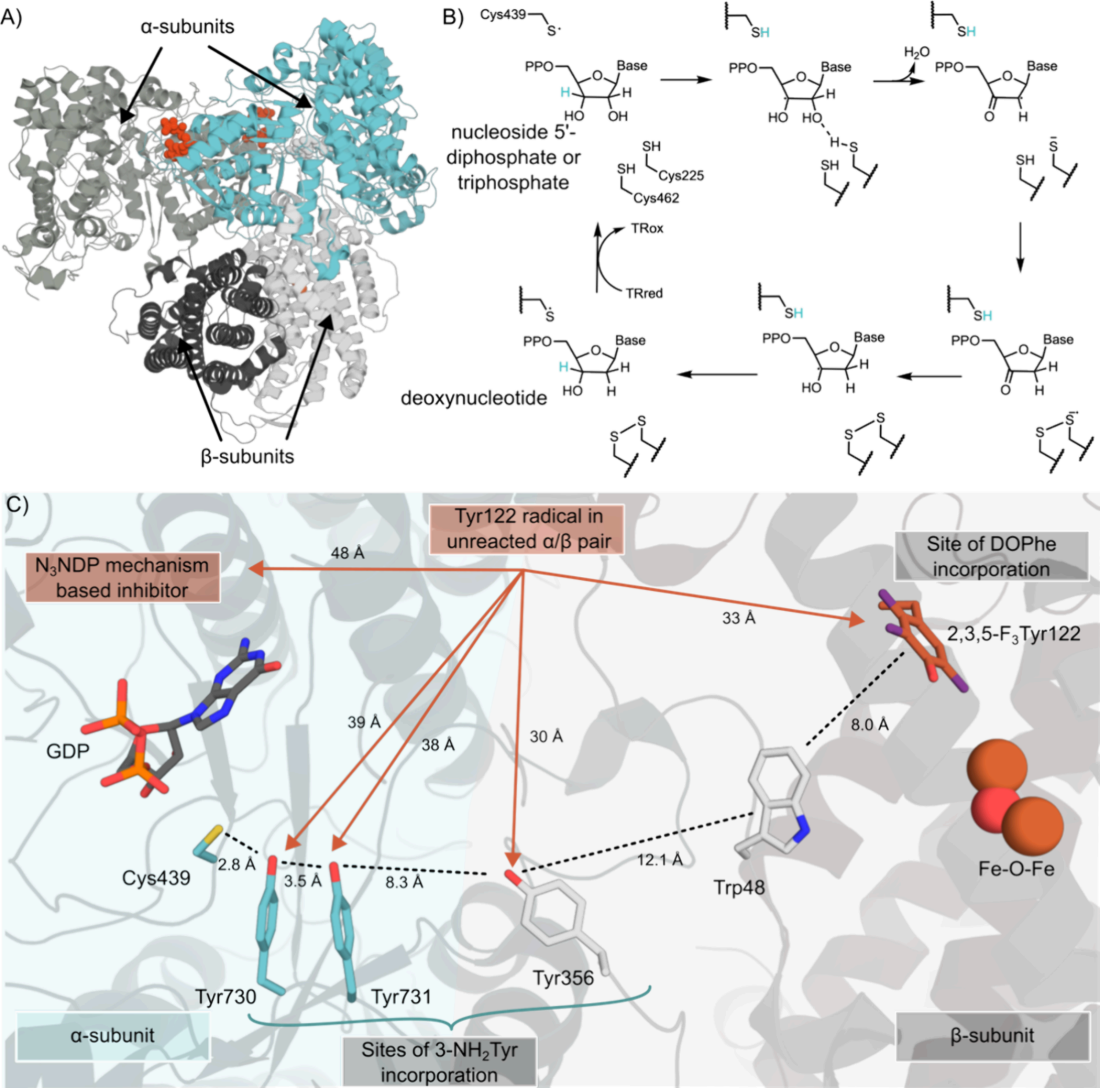
Mechanistic studies on RNRs using ncAAs have shed light on the electron transfer pathway and enabled structural characterization of the active form of the multimer. (A) A cryogenic electron microscopy structure of RNR (PDB: 6W4X(231)) in its active α2β2 form was captured using a 2,3,5-F_3_Tyr122 mutation. The protein chains are shown as cartoons, and GDP and TPP are shown as red and gray spheres, respectively. (B) The mechanism of RNRs, which catalyze the conversion of nucleoside di- and triphosphates to deoxynucleotides.^236^ TR = thioredoxin. (C) DEER experiments provided information on the relative distances between the Tyr122 radical in the unreacted α/β pair and radicals on an N_3_NDP mechanistic inhibitor or radicals trapped on 3-NH_2_Tyr.

Class I ribonucleotide reductases (RNRs) catalyze the conversion of nucleotides to 2′-deoxynucleotides through a mechanism that involves generation of a transient thiyl radical and long-range, reversible radical transfer between subunits α and β (Figure 6A,B).^236^ Early docking studies suggested the diferric-Tyr122 radical cofactor in the β-subunit is ∼ 35 Å from the oxidized residue Cys439 in the α subunit, with the proposed radical transfer pathway following Tyr122β → W49β → Tyr365β → Tyr731α → Tyr730α → Cys439α.^237^ To study the RNR mechanism, the oligomeric state of the enzyme, and to conclusively identify the redox active residues involved in radical transfer, the Stubbe laboratory has used ncAA incorporation to great effect (Figure 6).^236^ Mutation to canonical residues along the electron transfer pathway abolishes radical transfer, however, site specific introduction of fluorinated tyrosines (F_n_Tyr, n = 2–4),^238,239^ 3-aminotyrosine (3-NH_2_Tyr),^240,241^ and 3,4-dihydroxyphenylalanine (DOPhe, introduced by EPL methods)^242,243^ yields redox-competent variants of the two subunits (α and β). Replacing a Tyr residue along the radical pathway with 3-NH_2_Tyr or DOPhe traps the radical at that position, as it is unable to oxidize the next amino acid in the chain.^240^ UV–vis and EPR spectroscopies were used to quantify the presence of trapped DOPhe• or 3-NH_2_Tyr• radical signals. The stoichiometry of radical generation gave evidence for the active form of RNR requiring two α/β pairs (i.e., one radical equivalent per α2β2).

DEER spectroscopy has been used to measure the distance between a trapped 3-NH_2_Tyr• radical (generated at positions 356 in the β subunit or 730 and 731 in the α subunit) and a Tyr122 radical in the adjacent subunit pair (Figure 6C).^238^ Similarly, inter-radical distances have been derived between 3-NO_2_Tyr122• and Tyr356•, 3-NO_2_Tyr122•, or Tyr731• in adjacent subunit pairs.^244^ Additionally, the distance between Tyr122 and a nitrogen-centered radical covalently attached to Cys439 was measured using a mechanistic inhibitor, 2-azido-2-deoxycytidine diphosphate. The distances derived from DEER experiments are shown with red arrows in Figure 6C. Replacement of Tyr122 with 2,3,5-F_3_-Tyr or 3-NO_2_Tyr similarly generates 0.5 equivalents of radicals per α/β pair, further suggesting that RNR activity is reliant on formation of an α2β2 complex.^244^ The incorporation of 2,3,5-F_3_-Tyr at position 122, in combination with a Glu52Gln mutation, enabled structural characterization of the active α2β2 form by cryo-electron microscopy.^231^ The 2,3,5-F_3_-Tyr122 substitution allowed trapping of the radical at Tyr356 along the radical transfer pathway leading to tighter subunit affinity.

**Figure 7:**
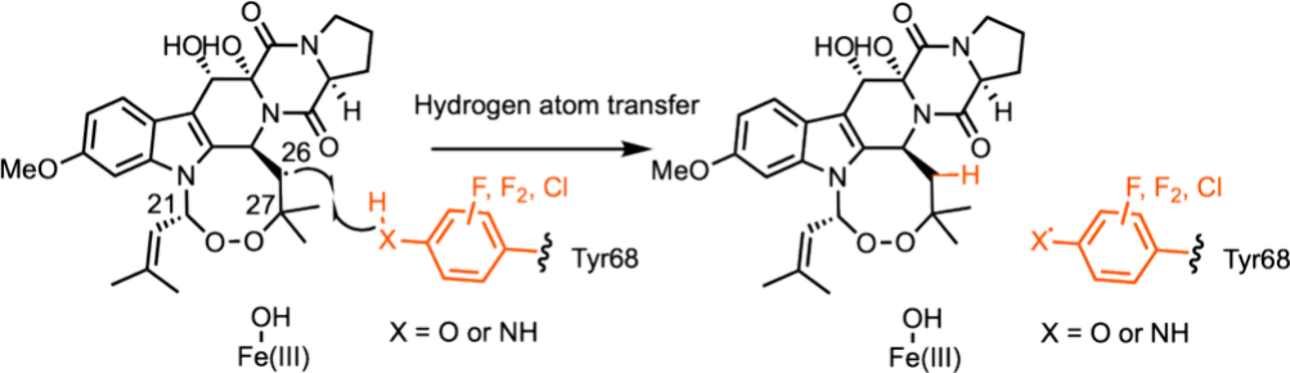
Mechanistic proposal for the FtmOx1-catalyzed hydrogen atom transfer from Tyr68 to C26•.

Noncanonical Tyr analogues were also used to investigate the role of a key catalytic residue in the Fe(II) and 2-oxoglutarate-dependent enzyme, Verruculogen synthase (FtmOx1).^245^ FtmOx1 installs an endoperoxide between C_21_ and C_27_ of fumitremorgin B using a high energy ferryl intermediate to break an allylic C_21_–H bond, followed by dioxygen capture, addition of the C_21_-O–O• radical to the C_27_ alkene, and hydrogen atom transfer (HAT) to the resulting C_26_• radical (Figure 7). Replacement of both active site Tyr residues (68 and 244) with ncAA analogue 3-FTyr facilitated unambiguous identification of Tyr86 as the hydrogen donor to C_26_•, with Tyr224 playing no essential role. Substitution of Tyr68 caused a blue shift in the sharp Tyr• absorption feature observed by stopped-flow absorption spectroscopy (maximum signal intensity observed at 0.35 s). X-band EPR spectra of samples freeze-quenched after 0.35 s exhibit large hyperfine coupling from ^19^F ortho to the phenolic oxygen radical that are not seen with the WT enzyme. These effects were not replicated in Tyr244 variants, confirming the role of Tyr68. Substitution of Tyr68 or Tyr244 with both 3-NH_2_Phe or 3-ClTyr preserved catalytic function. The radical species observed with 4-NH_2_Phe has a UV–vis absorbance spectrum in agreement with previously observed 4-methylaniline radicals and an EPR signal with additional hyperfine splittings from both ^14^N and the remaining proton.

The photoenzyme protochlorophyllide oxidoreductase (POR) catalyzes the reduction of protochlorophyllide to chlorophyllide.^246^ Recently, Taylor et al. replaced a key conserved tyrosine in the POR active site, which had previously been proposed to act as a proton donor in POR photocatalysis, with fluorinated Tyr analogues.^247^ Tyr fluorination led to a reduction in catalytic activity and impaired substrate binding. However, time-resolved laser spectroscopy reveals that the rate of proton transfer is almost identical in the WT and 3-FTyr193 variants. These data suggest that Tyr193 is unlikely to serve as a proton donor but instead is important for substrate binding.

*S*-adenosylmethionine (SAM)-dependent methyltransferases are known to form strong carbon-oxygen hydrogen bonds (CH···O) from an active site Tyr residue to their substrate SAM. To understand the importance of this interaction, Horowitz et al. incorporated 4-NH_2_Phe in place of Tyr335 in the active site of lysine methyltransferase SET7/9.^248^ This mutation was shown to be structurally conservative by X-ray crystallography, but reduced SAM binding affinity by ∼ 10,000 fold (to ∼ 1 mΜ). In contrast, SET7/9 Tyr355(4-NH_2_Phe) was able to bind the coproduct *S*-adenosyl-L-homocysteine with a similar affinity to that of the WT methyltransferase (∼900 μM), suggesting that the Tyr hydroxyl plays an important role in discriminating substrate vs product binding. The *k*_cat_ of SET7/9 Tyr355(4-NH_2_Phe) is 35-fold lower than that of the WT enzyme.

#### 3.3.2 Modulating Electric Fields

**Figure 8:**
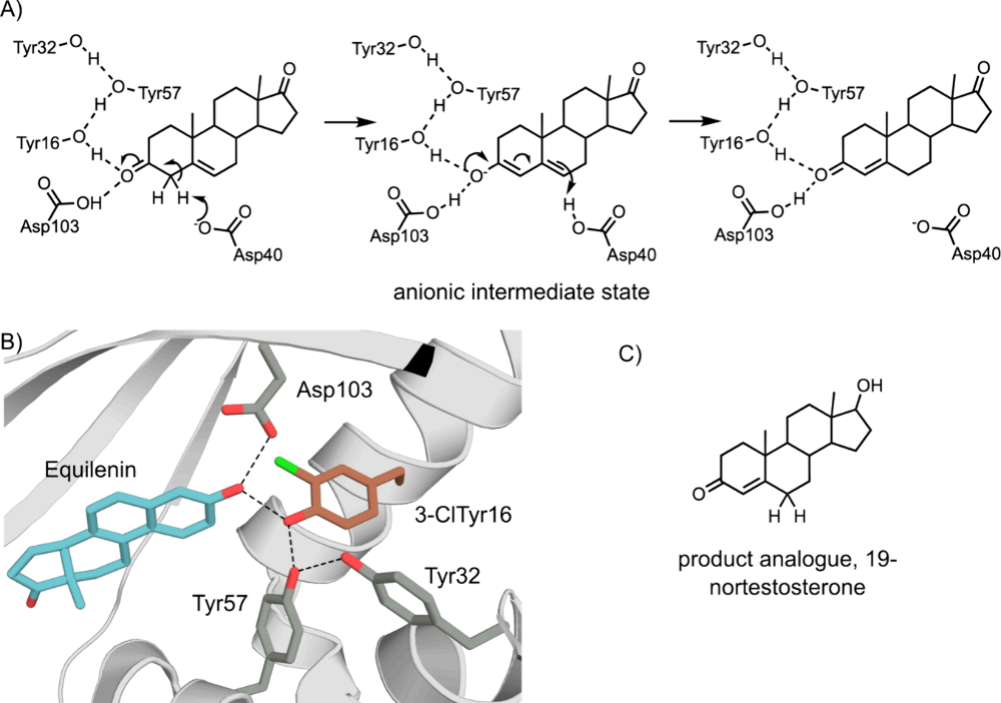
3-ClTyr incorporation into Ketosteroid Isomerase (KSI) to tune the active site electric field. (A) The mechanism of KSI. (B) The active site of KSI (PDB: 5KP1(254)) with the ncAA 3-ClTyr in the active site, shown with orange carbons. The protein backbone is shown as a gray cartoon. Active site residues and the substrate and transition state analogue equilenin are shown as atom-colored sticks, with gray and blue carbons, respectively. (C) The product analogue 19-nortestosterone used for VSE experiments.

Enzymes can apply strong electric fields to activate substrates in their active sites. VSE spectroscopy can be used to measure such effects, provided a suitable spectroscopic handle is available in the substrate or the protein.^249−251^ Ketosteroid isomerase (KSI) catalyzes a rate-limiting double bond migration of steroid substrates, which contain an IR-visible carbonyl bond (Figure 8A).^252^ The Boxer laboratory used VSE spectroscopy to observe the large electric field applied to the carbonyl of a product analogue (Figure 8C) in the KSI active site.^229^ This active site has an oxyanion hole consisting of Asp(H)103 and Tyr16, which forms part of an extended hydrogen-bonding network with Tyr32 and Tyr57 (Figure 8B).^253^ Using GCE, each of the three active site Tyr residues were substituted with 3-chlorotyrosine (3-ClTyr). The impact of these substitutions on the local electric field was measured using VSE spectroscopy and correlated with the changes in catalytic activity compared to the WT enzyme.^254^ The results show a linear relationship between the electric field applied to the carbonyl and the Δ*G*^⧧^, suggesting that the role of the Tyr16 hydroxyl and the adjacent hydrogen bonding network is to tune the electrostatic potential for efficient KSI catalysis.

#### 3.3.3 Noncanonical Amino Acids to Modulate Cation−π Interactions

Terpene synthases catalyze complex carbocation-based cyclization and rearrangement cascades to give a diverse set of terpenoid natural products. Aromatic ncAAs have been used to modulate cation−π interactions in intermediates in these enzymes.

NcAAs with a range of electron-withdrawing substituents were used to tune cation intermediate stabilization in aristolochene synthase.^255^ Replacement of a key active site residue Trp344 by Phe, 4-ClPhe, 4-CF_3_Phe, and 4-NO_2_Phe revealed a correlation between the electronic properties of the aromatic side chains and catalytic activity, with more electron-rich substituents leading to higher total turnovers, implicating residue 344 in stabilization of a key cationic intermediate.

A combination of canonical and noncanonical substitutions were used to interrogate cation-π interactions formed by two active site Phe residues in prokaryotic squalene-hopene cyclase (SHC), which mediates the conversion of squalene to hopene via a C_22_-hopanyl cation intermediate.^256^ Interestingly, substitution of Phe365 or Phe605 with the more electron-rich aromatics *O*-MeTyr, Tyr, and Trp, intended to increase the strength cation−π interactions, increases the catalytic rate at low temperature but leads to a rate reduction at higher temperatures, plausibly due to increased conformational disorder upon introduction of larger aromatic sidechains. To further investigate the effect of the cation-π binding energies on catalytic rates, Phe365 and Phe605 were systematically replaced by 4-FPhe, 3,4-F_2_Phe, and 3,4,5-F_3_Phe, which are of similar van der Waals radii to Phe. The catalytic rate decreases with increasing fluorination at either position in a manner proportional to the predicted cation-π binding energies of the aromatic side chains.

#### 3.3.4 Tuning Metal Coordination Environments

Introduction of noncanonical metal coordinating residues into proteins allows the electronic and steric properties of catalytic metal centers to be fine-tuned, providing new structure–activity relationships that cannot be obtained with standard amino acid substitutions.

**Figure 9:**
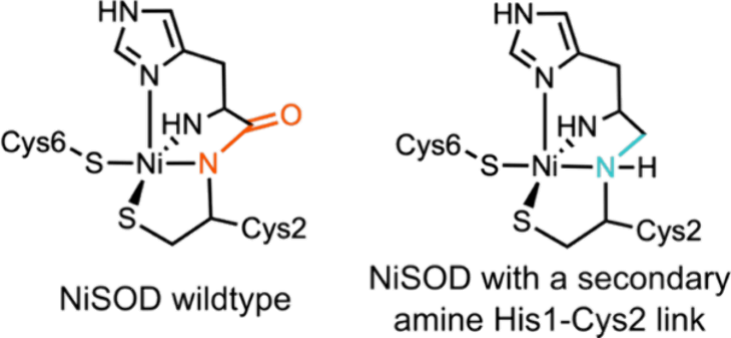
Active site of WT NiSOD (left) and a variant with a secondary amine backbone substitution (right).

Nickel-dependent superoxide dismutases (NiSODs) have a metal coordination environment comprised of two Cys ligands, the *N*-terminal amine, the imidazole of His1, and the backbone amidate of Cys2 (Figure 9).^257^ The catalytic nickel center can exist as a five-coordinate pyramidal Ni(III) or a four-coordinate planar Ni(II) species, which lacks the imidazole ligand of His1. To investigate the role of the backbone amidate ligand, a semisynthetic variant of NiSOD with the His1-Cys2 amide replaced by a secondary amine was prepared by NCL (Figure 9).^258^ This backbone modification leads to a ∼ 100 fold reduction in the catalytic rate. X-ray absorption near edge structure (XANES) and EPR spectroscopies reveal that the modified variant exists almost exclusively in the four-coordinate Ni(II) state, *ca*. 50:50 Ni(II)/Ni(III) mixture observed with WT preparations. These experiments suggest that the redox potential of the WT enzyme is finely tuned by the coordination environment to allow facile access to catalytically important Ni(II) and Ni(III) states.

The metal coordination environment of [NiFe]-dependent hydrogenase was modified by systematically replacing each of the four coordinating Cys ligands by Sec using *allo*-tRNAs and selenocysteine synthases.^259,260^ In all cases, the catalytic rate of Sec-containing hydrogenases were lower than the WT, retaining between 3 and 14% of the WT activity. However, replacement of the nonbridging Cys576 ligand, which coordinates the nickel cofactor at a position *cis* to the putative H_2_ binding site, led to increased oxygen tolerance. The authors suggest that the increased size of the Se atom may prevent O_2_ binding to the vacant metal coordination site, or alternatively that the more nucleophilic Sec attacks reactive oxygen intermediates to generate a selenoxide that is easily reduced to release H_2_O. Interestingly, Sec-containing thioredoxin (TR) has also been shown to be more oxygen tolerant than its Cys-containing counterpart.^261^ In another study, Wu et al. capitalized on the redox properties of selenocysteine to develop an artificial glutathione peroxidase by replacing the catalytic serine of subtilisin with Sec using a two-step chemical method. Interestingly this Sec modified subtilisin also displays acyl transferase activity.^262^

**Figure 10:**
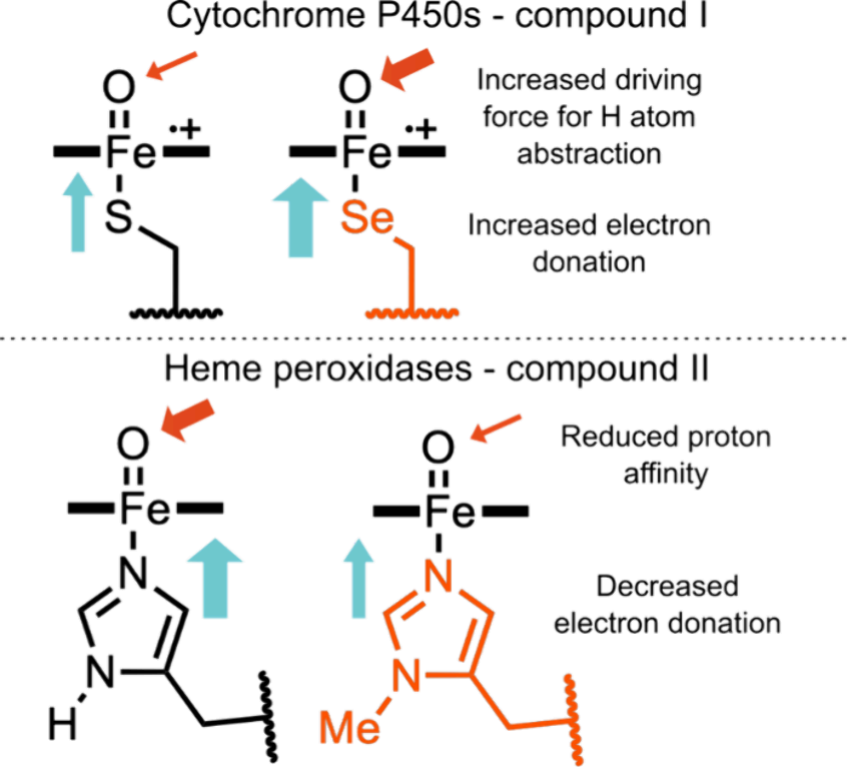
Electron donation to the iron center affects ferryl reactivity. (top) Cytochrome P450s are capable of hydrogen atom abstraction by the intermediate Compound I. Increased electron donation through an ncAA selenolate ligand increases the rate compared to WT P450. (bottom) Heme peroxidase compound II is reduced through proton coupled electron transfer. His to MeHis substitution decreases the electron donation to the ferryl intermediate and reduces its proton affinity, slowing the rate of compound II reduction.

The identity of the metal-coordinating axial ligand varies across heme enzyme families and is known to play a critical role in controlling catalytic function.^263,264^ As such, there is considerable interest in understanding the relationships between proximal ligand electron donation, the structures and reactivities of the Fe(IV)=O intermediates compounds I and II, and overall catalytic function (Figure 10).^265^ Natural cytochrome P450s contain Cys-ligated heme iron centers that can perform challenging oxidations of unactivated C–H bonds, chemistry which is inaccessible with histidine-ligated heme peroxidases, even though both enzyme classes use similar Fe(IV)=O intermediates.

The role of the cysteine axial ligand has been explored through its targeted replacement by selenocysteine in a variety of cytochrome P450 enzymes, including P450cam, CYP125, and CYP119.^266−270^ Upon mutating the axial Cys to Sec, spectroscopic features and spin states of P450s are similar to the WT enzymes, with minor red-shifts in the Soret maxima and changes in the Raman spectra that demonstrate that Sec is a more electron donating ligand than Cys. It was subsequently shown that the compound I state of Sec-ligated CYP119 was able to cleave C–H bonds more rapidly than the thiolate-ligated WT, providing a direct link between ligand strength and ferryl reactivity.^269^ This increased reactivity can be attributed to generation of a more basic compound I state in the Sec-ligated enzyme, increasing the driving force for H-atom abstraction.

A similar approach has been used to derive relationships between ligand strength and ferryl heme reactivity in heme peroxidases. These enzymes have an “imidazolate-like” axial ligand, that is more electron donating than a typical histidine, due to a hydrogen bond between the noncoordinating *N*_δ_ atom and a conserved aspartate in the proximal pocket. GCE was used to replace the axial His175 of cytochrome *c* peroxidase (C*c*P) by a less-electron donating MeHis, which lacks the proximal hydrogen bonding interaction to Asp235.^271,272^ This substitution was shown to be structurally conservative by X-ray crystallography and resulted in a ∼ 20-fold reduction in *k*_cat_ for ferrous cyt*c* oxidation with negligible changes in *K*_M_. In contrast to the situation encountered in P450s, ligand replacement in C*c*P had minimal impact on the reactivity of compound I (an oxidized neutral ferryl heme and a Trp191 radical cation). Instead, His175MeHis substitution caused a 10-fold reduction in the rate of proton-coupled electron transfer to compound II. This trend can be attributed to weaker electron donation from the axial MeHis ligand affording an electron deficient ferryl-oxygen with reduced proton affinity.

In a subsequent study, Trp51 in the distal pocket of C*c*P was replaced by a noncanonical 3-(benzothienyl)alanine (STrp), which is structurally similar to Trp but lacks the N–H moiety that forms a hydrogen bond to the ferryl oxygen in compounds I and II.^230^ Removal of this hydrogen bond stabilized compound I, but leads to a more basic and reactive compound II state. This increased compound II reactivity manifests in a > 60-fold increase in peroxidase activity toward the small molecule substrate guaiacol (2-methoxyphenol), but interestingly has minimal effect on the rate of oxidation of the biological redox partner, cyt*c*.

### 3.4 Mimics of Post-Translational Modifications

The structures and functions of native proteins are often tailored through PTMs. In many cases, these covalent modifications cannot easily be recapitulated in recombinant proteins, and as a result their functional significance can be difficult to elucidate.^273^ GCE offers a powerful approach to probe the role of PTMs, through selective introduction of ncAAs that are structural mimics of post-translationally modified residues.^274−276^

#### 3.4.1 Lysine and Tyrosine Modifications

Lys residues are subject to a range of reversible PTMs for regulating enzyme function, protein–protein interactions, and cellular localization.^277^ The genetic encoding of *N*_ε_-lysine derivatives using engineered tRNA^Pyl^/Pyl-tRNA synthetase pairs^278−283^ have been used to great effect in the study of individual PTM function in histones^284−289^ and enzymes throughout the citric acid cycle.^290−293^ For example, acetylation of Lys295 decreases citrate synthase activity by 10-fold, whereas acetylation of Lys238 leads to a 2-fold activity increase.^294^ GCE was also used to incorporate *N*_ε_-acetylLys (AcLys) into the selenoprotein thioredoxin reductase, to investigate the effect of the PTM on enzyme activity and regulation.^295^ A series of thioredoxin reductase variants with acetylated Lys at positions 141, 200, and 307 were all shown to have increased activity compared with the parent enzyme. The authors propose that AcLys incorporation destabilizes inactive multimeric states and favors the active dimeric form of the enzyme.

Dual incorporation of phosphoserine (Sep)^296^ and AcLys was used to mimic the coexisting acetylation and phosphorylation PTMs present in native malate dehydrogenase (MLDH).^297^ Lysine acetylation at position 140 increased enzyme activity by 3.5-fold, while a single Ser280Sep mutation reduced activity to *ca.* 30% of the WT enzyme. Simultaneous incorporation of both ncAAs restored the MLDH activity to WT levels, suggesting the two PTMs work in synergy to moderate the activity. Structural modeling of the homotetramer reveals positions 140 and 280 are in proximity at the dimeric interface, opposite a hydrophobic surface region of the protein, suggesting that the PTMs serve to modulate surface charge and stabilize the oligomeric state.

Stop codon suppression (SCS) was used to incorporate *N*_ε_-threonyllysine (ThrLys) to study the effect of this recently discovered PTM on Aurora Kinase A activity.^298^ Threonylation of Lys162 completely inhibits kinase activity toward a synthetic heptapeptide, with computational modeling suggesting that the modified side chain occupies the ATP binding site. This inhibitory effect can be reversed by the deacetylase Sirtuin 3, which removes the threonylated group from the Lys162.

Human glycolysis enzymes have been found to be heavily lactylated,^299^ with lactylation of fructose-bisphosphate aldolase A at the active site Lys147 being a particularly prevalent protein modification. GCE was used to replace Lys147 by a noncanonical lactyllysine (LacLys), which resulted in inhibition of enzyme activity. These results led the authors to propose a lactylation-dependent negative feedback loop in glycolysis, whereby enzymes upstream of an overactivated glycolysis pathway are inhibited leading to reduced glycolytic flux and decreased lactate levels.

Tyrosine PTMs can also be studied using ncAA analogues. 4-carboxymethyl-Phe (4-CmPhe) was used as a mimic of phosphotyrosine to study how the PTM regulates function in the SAM-dependent enzyme protein arginine methyltransferase 1 (PRMT1).^275^ Tyr291(4-CmPhe) substitution did not affect the kinetic parameters for methylation of histone H4, but caused a large increase in the *K*_M_ for two peptides that mimic either the histone H4 tail region or the site of methylation. These data suggest that the phosphorylation of Tyr291 plays a role in the modulation of the substrate specificity of PRMT1 *in vivo*.

#### 3.4.2 Noncanonical Amino Acids to Mimic Post-Translational Cross-Links

Thioether-bonded tyrosine–cysteine cross-links (Tyr-Cys cross-links) are a PTM common to a range of metalloenzymes, including galactose oxidase,^300^ cytochrome *c* nitrite reductase,^301^ and cysteine dioxygenase.^302^ To explore the role of this PTM, a model of *T. nitratireducens* cytochrome *c* nitrite reductase (*Tv*NiR) was developed in sperm whale Mb with either Tyr or 2-amino-3-(4-hydroxy-3-(methylthio)phenyl)propanoic acid (3-MeS-Tyr) at position 33, to mimic the Tyr-Cys cross-link of the WT nitrite reductase.^303^ Hydroxylamine reduction activity increased 4-fold with 3-MeSTyr in place of Tyr33. These activity improvements can be attributed to the 3-methylthio modification lowering the p*K*_a_ and reduction potential of the active site Tyr, leading to an increased rate of proton coupled electron transfer to the substrate.

**Figure 11:**
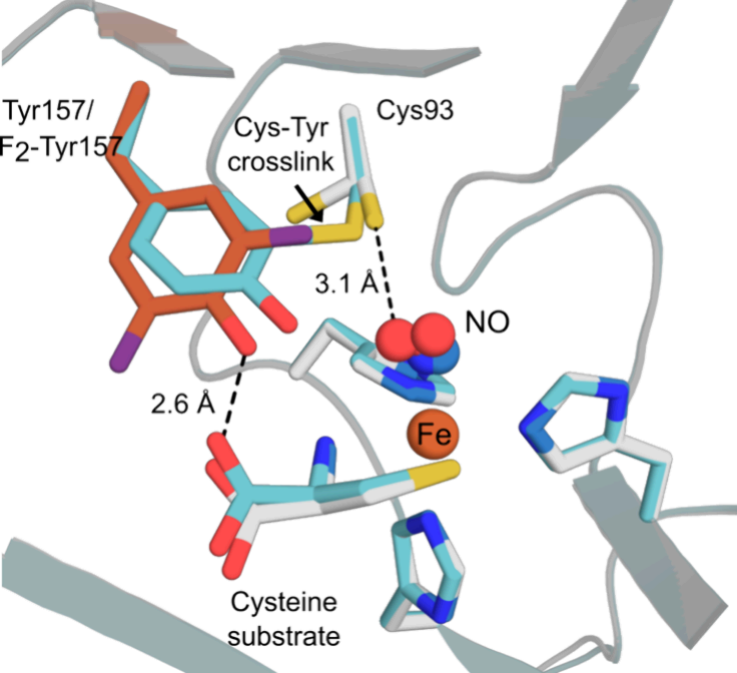
Anaerobic X-ray crystal structures of the active sites of Human Cysteine Dioxygenase (CDO, PDB: 6N43(306)) and CDO Tyr157F_2_-Tyr (PDB: 6BPR(306)) in complex with the substrate cysteine and NO. CDO and CDO Tyr157F_2_-Tyr are shown as cartoons in blue and gray, respectively, with key active site residues and the substrate cysteine shown as atom-colored sticks with blue and gray carbon atoms. The noncanonical F_2_-Tyr157 is shown with orange carbon atoms.

NcAA incorporation can also be used to study Tyr-Cys cross-link formation, which is often challenging due to the rapidity of the oxidative cross-linking reaction. The nonheme iron enzyme cysteine dioxygenase (CDO) features a Tyr157-Cys93 cross-link adjacent to the iron center which increases catalytic efficiency by around 10-fold.^304^ Site-specific substitution of Tyr157 for 3,5-F_2_-Tyr and subsequent crystal growth under anaerobic conditions enabled acquisition of a noncross-linked structure while retaining Cys93 (Figure 11).^305^ Interestingly, exposure of CDO 3,5-F_2_-Tyr193 crystals to oxygen resulted in cross-link formation and cleavage of one of the C–F bonds of the ncAA.^306^ The positioning of the precross-linked residues in the CDO 3,5-F_2_-Tyr193 structure suggests that the first target for oxidation during the cross-linking reaction is Cys93, given its proximity to the dioxygen binding site of the metal ion.

OvoA is a nonheme iron enzyme that catalyzes the oxidative coupling of histidine and cysteine substrates, but also possesses ∼ 10% competing cysteine dioxygenase activity despite lacking a Tyr-Cys cross-link. Interestingly, replacement of active site Tyr417 with 3-MeSTyr alters the product distribution of the enzyme, with 30% of the Cys substrate oxidized to the corresponding sulfinic acid.^307^

The functional importance of post-translational Tyr-His cross-links can also be interrogated using ncAAs. Heme copper oxidases (HCOs) selectively catalyze the four electron reduction of molecular oxygen to water without releasing reactive oxygen species (ROS).^308^ The copper center is coordinated by three His residues, one of which forms a cross-link from the N_ε_ atom to the C6 of a neighboring Tyr residue. To better understand the mechanistic significance of this conserved cross-link,^309^ Liu et al. incorporated (*S*)-2-amino-3-(4-hydroxy-3-(1H-imi-dazol-1-yl)phenyl)propanoic acid (ImiTyr) at position 33 in a previously reported functional HCO model based on Mb (*vide supra*).^310^ The resulting enzyme performed over 1000 cycles of oxygen reduction with less than 6% ROS produced, demonstrating 8-fold higher H_2_O/ROS selectivity and 3 times as many turnovers as the previous HCO model that lacks a Tyr-imidazole cross-link.^311^ A related study investigated the effect of Tyr33 p*K*_a_ in the HCO model by incorporating a series of Tyr analogues by GCE.^312^ The rate of O_2_ reduction was found to be inversely proportional to the p*K*_a_ of the phenolic proton, consistent with a mechanism in which Tyr33 acts as a proton donor to facilitate O–O cleavage. Interestingly, introduction of 3-MeTyr, which has a similar p*K*_a_ to Tyr but a lower reduction potential, also gave an increase in rate compared to the Tyr33 variant.^313^

## 4 Augmenting Function

Augmenting the functions and properties of existing enzymes is a cornerstone of modern biocatalysis. The ability to improve and control enzyme activity, selectivity, and tolerance to process conditions is vital in enabling the full industrial potential of biocatalysts to be realized. Though a large degree of enzyme optimization can be attained using cAAs, the expanded structural and chemical diversity offered by ncAAs can provide new avenues to access optimized biocatalysts. Many enzyme properties can be augmented using ncAAs, including stability and reusability, substrate and product selectivity, catalytic efficiency, and regulation of activity.

### 4.1 Stability and Immobilization

Enzymes deployed in industrial processes must often withstand elevated temperatures and organic solvents, and are more cost-effective when recovered and reused across multiple reaction cycles.^314,315^ NcAAs can be an effective tool to improve enzyme stability and solvent tolerance, and can open up new biorthogonal chemistries to immobilize enzymes in defined orientations on solid supports. A variety of approaches can be used to achieve stabilization and immobilization, reflecting the diversity of ncAAs available and the different attributes of SPI and SCS methodologies. This section reviews examples of enzyme stabilization achieved through ncAA-mediated noncovalent interactions, covalent cross-links within and between macromolecules, and site-specific immobilization on a range of solid supports.

#### 4.1.1 Stabilization *via* Noncovalent Interactions

NcAAs can be used to stabilize enzymes by strengthening favorable noncovalent interactions without disrupting tertiary structure. Incorporation of halogenated ncAAs has often been shown to increase enzyme thermostability,^316−318^ an effect attributed to dipolar interactions or the formation of halogen bonds between the positive σ-hole of the halogen atom and a neighboring lone pair or negatively charged group.^319^ Halogenated ncAAs that are isostructural to their canonical counterparts are often well-accommodated in enzymes, with the similarity between C–F and C–H bond lengths being particularly advantageous in this regard.^25,320^ Enzyme stabilization using less conservative ncAA modifications has also been investigated, most commonly employing aliphatic side chain substitutions designed to increase hydrophobic interactions.^321,322^ The examples discussed below illustrate how ncAA incorporation can increase thermostability and solvent tolerance across a range of enzyme classes, and in some cases that these improvements can also correlate with enhanced catalytic performance.

Thermostable and solvent-tolerant lipases are widely used in many chemical industries.^323,324^ Budisa et al. globally substituted 5-FTrp, 3-FTyr or 4-fluorophenylalanine (4-FPhe) for their respective cAAs in *Candida antarctica* lipase B (CalB) *via* SPI.^325^ Despite some moderate changes in secondary structure (as observed by circular dichroism), all three variants retained lipase activity, and exhibited an increased shelf life. In particular, the 4-FPhe variant was 50% more active than the WT enzyme following storage for several months at 4 °C. A similar study globally incorporated 6-FTrp, 4-FPhe and 4-fluoroproline (4-FPro) simultaneously into *Thermoanaerobacter thermohydrosulfuricus* lipase (TTL), substituting almost 10% of the residues with fluorinated analogues.^326^ Surprisingly, this extensive fluorination had minimal effects on secondary structure or enzyme activity, with the modified enzyme retaining around 60% of the WT activity. A subsequent study investigated the effect of separate global substitution of ten different ncAAs into TLL, including fluorinated Phe, Tyr and Pro, as well as norleucine and azidohomoalanine.^327^ All variants evaluated retained at least some activity, with the 3-fluorophenylalanine (3-FPhe) variant displaying 25% greater efficiency than the WT. Interestingly, this variant also exhibited an expanded substrate scope, with a broader range of triacylglycerol chain lengths (C_2_-C_18_) accepted compared to the WT (C_6_-C_10_). A further study incorporated 13 different ncAA analogues into TTL and investigated the solvent and surfactant tolerance of the resulting variants.^321^ In many cases, these substituted variants displayed an increased tolerance to organic solvents compared to the WT, with some exhibiting higher activity in solvent compared to in aqueous buffer. For example, SPI of 4-*R*-fluoroproline (4-*R*-FPro) displayed a 4.5-fold increase in activity in *tert*-butanol and 3.9-fold in acetonitrile. Many of the variants were also more resistant to denaturation, with 4-aminotryptophan (4-NH_2_Trp) and 3-FPhe substitutions enabling 70% of activity to be retained after incubation in 0.5 M guanidinium chloride, conditions which led to complete inactivation of the WT enzyme.

Transaminases are important biocatalysts due to their ability to produce enantiopure amines, a motif found in a large proportion of pharmaceutical intermediates and chemical commodities.^328,329^ SPI of 3-FTyr into ω-transaminase from *Vibrio fluvialis* JS17 resulted in a soluble enzyme with 20% higher transaminase activity compared to the WT.^317^ Thermostability was also enhanced, with a two hour 70 °C incubation reducing the activity of the fluorinated variant to 36%, compared to only 3.3% for the WT. The fluorinated enzyme also demonstrated increased solvent tolerance, with 90% of activity retained in 50% *v*/*v* DMSO cosolvent, whereas the WT exhibited a comparable loss in activity in less than 10% DMSO. Importantly, substrate specificity and enantioselectivity were unaffected by 3-FTyr incorporation, enabling the modified transaminase to catalyze the production of enantiopure (*S*)-1-phenylethylamine on a preparative scale in 89% isolated yield. A later study demonstrated *in vivo* biosynthesis of 2-fluorotyrosine (2-FTyr) *via* transgenic tyrosine phenol lyase and subsequent incorporation into two ω-transaminases from *Sphaerobacter thermophilus*.^330^ The resulting enzymes exhibited high levels of 2-FTyr incorporation and increased thermostability compared to their parent enzymes. Again, substrate selectivity was preserved, and in a high temperature kinetic resolution of racemic α-methylbenzylamine the fluorinated variants yielded the *R* enantiomer of the amine to > 99% *ee*, compared to under 50% for the WT enzymes.

**Figure 12:**
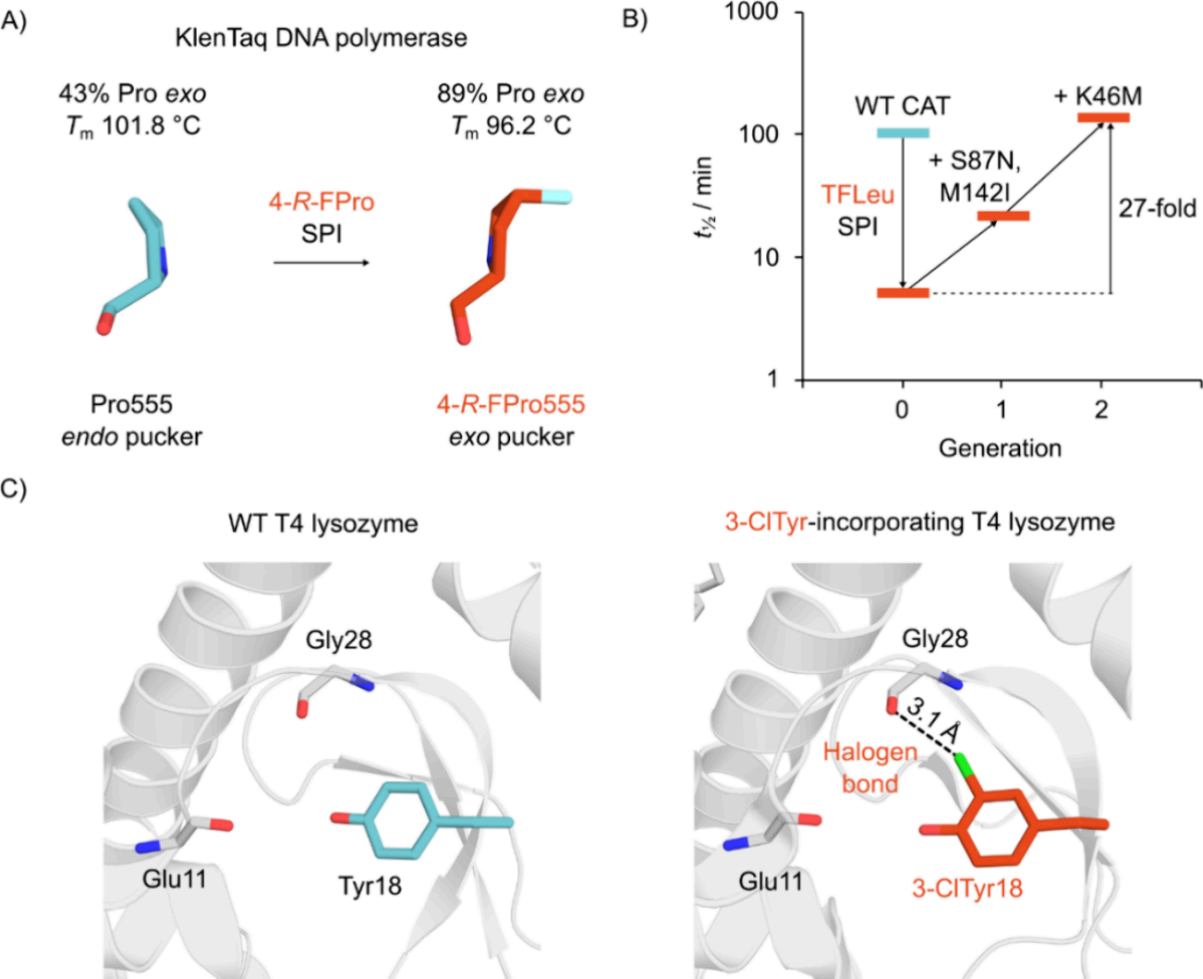
NcAA-mediated noncovalent interactions influence enzyme stability. (A) SPI of 4-R-FPro in KlenTaq DNA polymerase switches many Pro puckers from endo to exo, as illustrated by the substitution of Pro555 (left, gray carbons) to 4-R-FPro555 (right, orange carbons) (PDB: 4DLG, 4DLE(335)). (B) Evolutionary trajectory of TFLeu-incorporating CAT (orange bars) starting from WT CAT (gray bar) against the half-life of enzyme inactivation at 60 °C. (C) Structures of T4 lysozyme with canonical Tyr18 (left, gray carbons) and noncanonical 3-ClTyr18 (right, orange carbons). Glu11 and Gly28 backbone atoms shown (white carbons). Halogen bond between Gly28 backbone oxygen and 3-ClTyr18 chlorine atom indicated with a dashed line (PDB: 1L63,^340^5V7E^339^).

In some cases, global replacement of residues with halogenated analogues can simultaneously introduce both beneficial and deleterious mutations into an enzyme. Votchitseva et al. globally incorporated 3-FTyr into a phosphotriesterase (PTE),^331^ an enzyme class which has garnered interest for their ability to degrade organophosphate-based pesticides and chemical warfare agents for the purposes of bioremediation.^332,333^ The modified PTE exhibited an extended pH tolerance down to pH 5.5, plausibly due to the lower p*K*_a_ of 3-FTyr compared to Tyr, as well as increased thermostability, with preincubation at 60 °C having minimal effect on paraoxon hydrolysis activity compared to ∼ 90% inactivation of the WT. However, under optimum conditions the catalytic efficiency of the variant was ∼ 400-fold lower than WT, plausibly due to Tyr309 substitution in the leaving group binding pocket, highlighting how ncAA incorporation at residue-level specificity can simultaneously introduce desirable and undesirable effects at different sites. A similar study globally replaced all 32 Pro residues in KlenTaq DNA polymerase with 4-*R*-FPro.^334^ Despite the large number of substitutions made and the highly dynamic nature of polymerases during catalysis, the fluorinated variant retained DNA extension activity, exhibiting 94% of the WT dNTP consumption rate and a similar fidelity. However, thermostability was somewhat reduced, with residual activity only half that of the WT after a one hour incubation at 95 °C. Crystallization of the fluorinated polymerase^335^ revealed that 4-*R*-FPro has a tendency to adopt an *exo* puckered conformation, forcing some positions to adopt this conformation rather than their WT *endo* pucker (Figure 12A), which may have contributed to the reduction in stability. Interestingly, the authors noted that the fluorinated variant exhibited enhanced crystallizability compared to the WT, with crystals formed under a broader range of conditions. Several surface 4-*R*-FPro positions were identified where the fluorine atoms were in close proximity to neighboring symmetry chains, potentially forming new crystallization contacts.

Enzyme engineering can be used to overcome deleterious effects arising from global ncAA incorporation. Panchenko et al. investigated the effects of global leucine replacement with 5′,5′,5′-trifluoroleucine (TFLeu) in CAT.^336^ The fluorinated enzyme retained 76% of WT activity but was much less thermostable, exhibiting a 27-fold reduction in half-life at 60 °C. TFLeu-containing CAT was subsequently evolved by screening acetyltransferase activity after preincubation of lysates at 60 °C for one hour.^90^ After two rounds, a triple mutant was identified which exhibited comparable thermostability and activity to the nonfluorinated WT CAT (Figure 12B). Introduction of the same mutations into the nonfluorinated enzyme had a much smaller effect on half-life, demonstrating the specificity of the mutations for adapting the enzyme to TFLeu incorporation. Another study globally incorporated 4-FPhe into dimeric S5 PTE, leading to improved activity and stability.^316^ The fluorinated enzyme displayed improved catalytic efficiency toward paraoxon and 2-napthylacetate hydrolysis (2.9-fold and 4.7-fold respectively). Preincubation at 65 °C led to complete inactivation of the WT, while the 4-FPhe variant retained up to 41% activity. Differential scanning calorimetry revealed that 4-FPhe incorporation increased the *T*_m_ of the two endothermic transitions by 1–2 °C. More notably, for samples subjected to a second heating scan, the WT showed no transitions whereas the two transitions were retained for the fluorinated variant, suggesting that the modified enzyme could refold after thermal denaturation. However, the authors noted the fluorinated variant gave substantially poorer yield of soluble protein compared with the WT enzyme, suggesting that not all 4-FPhe substitutions were beneficial. In a follow-up study, all phenylalanine positions in the PTE were interrogated *in silico* with Rosetta modeling software.^337^ The positions were all replaced with 4-FPhe, then singly mutated to each of the 19 other cAA identities, to discover mutations which gave a better predicted energy score than 4-FPhe. Through this investigation, 4-FPhe104Ala was identified as potentially beneficial to enzyme stability, as it relieved a steric clash arising from 4-FPhe substitution at the dimer interface. Expression of this 4-FPhe-F104A mutant doubled soluble protein yield and increased the thermal unfolding transition temperatures. Paraoxon hydrolase activity was retained at similar levels to WT PTE, and the mutant was more stable under storage conditions, with 66% residual activity recorded after seven days compared to < 50% for WT PTE both with and without 4-FPhe incorporation. Interestingly, introducing the F104A substitution into otherwise unmodified PTE dramatically decreased stability and activity, demonstrating successful prediction of a 4-FPhe context-specific stabilizing mutation by the Rosetta protocol.

SCS offers a more targeted approach to ncAA-mediated enzyme stabilization, by enabling incorporation only at positions where the ncAA confers a benefit. Ohtake et al. systematically replaced each of the 15 Tyr residues in microbial transglutaminase (MTG) with 3-ClTyr *via* suppression of the UAG stop codon.^318^ The single mutants at positions 20 and 62 exhibited increased residual activity relative to the WT following preincubation at 60 °C, while the other mutations tested were either neutral or deleterious. Testing combinations of the beneficial and neutral mutations revealed a triple mutant with a 5.1-fold longer half-life than the WT as well as a 1.4-fold increase in activity. The researchers then demonstrated simultaneous incorporation of an α-hydroxy acid analogue of *N*_ε_-allyloxycarbonyllysine (ALOLysOH) immediately after an *N*-terminal inhibitory peptide domain *via* sense codon reassignment. This generated a thermostabilized MTG variant which undergoes automaturation in basic conditions due to hydrolysis of the backbone ester bond.

Another study utilized 3-bromotyrosine (3-BrTyr) for the stabilization of azoreductase (AzoR).^338^ Each Tyr residue in AzoR was individually replaced with 3-BrTyr, revealing three positions where bromination was beneficial to thermostability. Simultaneous substitution at these three sites yielded a variant with a 13-fold longer half-life than the WT enzyme at 78 °C. The authors also subjected GST to a similar engineering strategy, substituting Tyr residues with 3-ClTyr. Systematic identification and combination of beneficial substitutions generated a variant with four 3-ClTyr incorporation sites that exhibited 79% residual activity after a 10 min preincubation at 60 °C, compared to < 5% for WT GST.

SCS can also be used to install single substitutions explicitly designed to introduce new stabilizing interactions. By supplementing an *in vitro* translation reaction with chemically aminoacylated suppressor tRNAs, Mendel and co-workers substituted Leu133 in the hydrophobic core of T4 lysozyme with a variety of ncAA analogues chosen to sample a range of sizes and shapes for the aliphatic side chain without disrupting neighboring residues.^322^ They correctly predicted that enzyme thermostability would correlate with ncAA side chain size, with norvaline and ethylglycine reducing *T*_m_, and 2-amino-4-methylhexanoic acid and 2-amino-3-cyclopentylpropanoic acid increasing it by up to 4.3 °C, due to their larger side chains establishing more extensive hydrophobic interactions. In a different study, Scholfield et al. engineered a halogen bond into T4 lysozyme by substituting Tyr18 with 4-iodophenylalanine (4-IPhe).^320^ Crystal structures of the modified and WT enzyme evidenced the formation of a halogen bond between the iodine atom and the backbone carbonyl of Glu11, coupled with displacement of the aromatic Tyr ring from its WT position. Replacement of the iodine with a methyl group did not result in the same displacement. However, 4-IPhe incorporation slightly decreased overall enzyme stability due to disruption of favorable hydrogen bonds made by the 4-hydroxyl of the WT tyrosine. A subsequent study incorporated 3-ClTyr at the same site.^339^ This modification preserved hydrogen bond interactions made by the hydroxyl group and introduced a new halogen bond between the chlorine and the carbonyl oxygen of G28 (Figure 12C), leading to a 1.0 °C increase in *T*_m_ and a 15% increase in enzymatic activity at 40 °C compared to the WT.

#### 4.1.2 Stabilization *via* Covalent Cross-Linking

**Figure 13:**
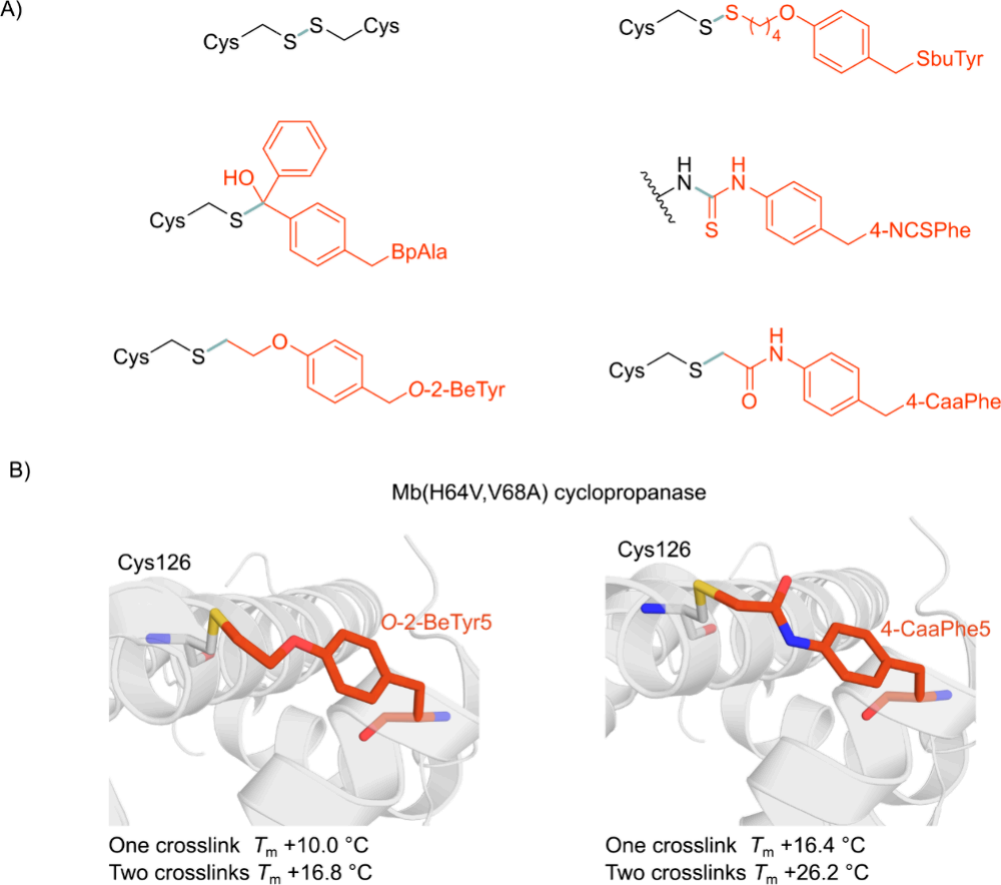
Covalent cross-links mediated by ncAAs. (A) Cross-links generated between cAAs (black) and ncAAs (orange). Cross-linking bonds shown in gray. Top left: canonical Cys-Cys cross-link. Top right: Cys-SbuTyr cross-link. Middle left: Cys-BpAla cross-link. Middle right: amino group-4-NCSPhe cross-link. Bottom left: Cys-O-2-BeTyr cross-link. Bottom right: Cys-4-CaaPhe cross-link. (B) Structures of Cys-O-2-BeTyr cross-link (left) and Cys-4-CaaPhe cross-link (right) in Mb(H64V,V68A), with *T*_m_ increases given by one and two cross-links indicated. ncAAs shown with orange carbons and Cys with white carbons (PDB: 7SPE, 7SPH(351)).

Disulfide bridge formation through oxidative linking of Cys side chain thiols has long been known to enhance enzyme stability,^341,342^ and engineering new disulfide bridges into enzymes has proven an effective method of stabilization.^343,344^ However, disulfide links between canonical Cys residues are subject to geometric and chemical constraints that limit the range of enzymes in which these linkages can be successfully installed.^345^ The use of noncanonical analogues can overcome some of these constraints, enabling more facile and diverse deployment of covalent cross-linking for enzyme stabilization (Figure 13A). Liu et al. developed noncanonical Tyr analogues derivatized with aliphatic thiols through the *para* hydroxyl motif, enabling formation of longer disulfide cross-links.^346^ SCS was used to incorporate the thiol-containing ncAAs in a library of *N*-truncated TEM-1 β-lactamase where one random codon per gene was replaced with TAG. The *N*-terminal truncation was previously shown to destabilize the β-lactamase such that it could no longer confer antibiotic resistance to the host cell above 37 °C.^347^ Growth of library-transformed cells in the presence of the lactam antibiotic ampicillin at 40 °C was used to select for active, stabilized enzyme mutants. A single hit was identified containing a disulfide bridge formed between a Cys residue at position 65 and the thiol ncAA *O*-(4-mercaptobutyl)tyrosine (SbuTyr) at position 184, which increased the *T*_m_ by 9 °C compared to the parent enzyme. Analysis of WT crystal structures revealed the cross-link was formed across the hinge region of two half-domains, which had previously been identified as a hotspot for stabilizing mutations.^348^ Interestingly, the distance between the two β carbons of residues 65 and 184 was 11.1 Å, far exceeding the maximum distance of ∼ 5.5 Å for a canonical Cys disulfide.^345^

The use of ncAAs can also improve the chemical stability of covalent cross-links. Moore and co-workers developed redox-stable thioether “staples” formed between Cys thiols and *O*-2-bromoethyltyrosine (*O*-2-BeTyr) residues^349^ (Figure 13B). The RosettaMatch algorithm was used to identify positions in a cyclopropanation-competent Mb variant which could accommodate Cys-*O*-2-BeTyr cross-links in a strain-free configuration, as well as compensatory mutations of surrounding residues to maximize favorable interactions around the staples. Out of nine designs experimentally evaluated, five successfully formed thioether cross-links, all of which exhibited *T*_m_ increases ranging from 3.9 to 10.0 °C over the parent enzyme. Substitution of *O*-2-BeTyr with isosteric but noncross-linking *O*-propargyltyrosine (*O*-PaTyr) lowered the *T*_m_ values to below WT, confirming the thioether cross-links were essential for the observed stability increases. Combination of the two most stabilized variants resulted in a doubly stapled design with a 16.8 °C higher *T*_m_ than the parent enzyme. Impressively, this variant also retained high levels of activity and stereoselectivity for styrene cyclopropanation, as well as exhibiting enhanced organic solvent tolerance. A different study conducted a similar RosettaMatch protocol on a multimodular pullulanase.^350^ Installation of Cys-*O*-2-BeTyr cross-links in the *N*/*C*-terminal domains increased *T*_m_ by up to 7.0 °C, though hydrolase activity was adversely affected in the majority of designs. Compensatory mutations designed to improve packing around the cross-links yielded a stabilized variant with 44% higher activity than the WT. A later study investigated the use of alternative cross-linking ncAAs.^351^ Substitution of *O*-2-BeTyr in the aformentioned Mb variants with phenylalanine derivatives containing a chloroacetamido (4-CaaPhe), acrylamido (4-AaPhe) or vinylsulfonamido (4-VsaPhe) group led to successful cross-link formation and increased *T*_m_ values relative to the WT enzyme. Notably, 4-CaaPhe incorporation led to even larger stability improvements than *O*-2-BeTyr, with up to a 16.4 °C increase in *T*_m_ given by a single Cys-4-CaaPhe cross-link, and a 26.2 °C increase for two cross-links. The 4-CaaPhe-stabilized mutants were shown to retain the same levels of cyclopropanation activity and stereoselectivity as the parent enzyme, with experimental crystal structures showing good agreement with the design models (Figure 13B). Intriguingly, 4-CaaPhe incorporation was also able to rescue some designs which had previously failed to form cross-links using *O*-2-BeTyr, suggesting a higher stapling efficiency and greater tolerance of 4-CaaPhe to different local environments.

NcAA-mediated cross-links can also be used to stabilize interactions between protein monomers. Homodimeric homoserine *O*-succinyltransferase (MetA) catalyzes an essential step in methionine biosynthesis in *E. coli*, but possesses only mesophilic thermostability which leads to inhibited cell growth above 44 °C.^352^ Li et al. exploited this property by screening a BpAla scanning library of MetA variants for increased growth of cells lacking native MetA at 44 °C.^353^ A consensus Phe21BpAla mutation emerged which conferred substantially faster growth on the host cells. Isolation of the mutant enzyme revealed a remarkable 21 °C increase in *T*_m_ over the WT. Position 21 is located near to the dimer interface, suggesting the possibility of a cross-link spanning the two subunits. Mutation of nucleophilic residues in the region facing Phe21 across the interface revealed that Cys90 was essential to the increase in stability, with a Cys90Ser substitution reverting *T*_m_ back to that of the WT. Further evidence of a Cys-BpAla cross-link was provided *via* trapping of the adduct with β-mercaptoethanol and ^13^C NMR studies. An alternative enzyme-stabilizing covalent linkage strategy was developed by Xuan et al..^354^ 4-Isothiocyanate phenylalanine (4-NCSPhe) was shown to react with Lys side chains, forming covalent thiourea adducts. The salt bridge formed between Lys17 and Asp123 in Mb was replaced with a thiourea cross-link via incorporation of 4-NCSPhe at position 123, which increased the *T*_m_ by 4.8 °C compared to the WT. A subsequent study utilized 4-NCSPhe to create stabilizing cross-links in MetA.^355^ Using the same elevated temperature cell growth assay described above, a Phe264(4-NCSPhe) mutant was identified which gave a dramatic 24 °C increase in *T*_m_ over the WT. Though initially only the monomeric species was observed after SDS-PAGE analysis, it was found that incubation of the mutant at 37 °C led to the appearance of a dimeric species, suggesting that cross-link formation was temperature dependent. The increased thermostability corresponded with increased transferase activity at higher temperatures, with over 60% residual activity recorded at 60 °C, compared to negligible activity of the WT above 50 °C. Proteolysis and mass spectrometry (MS) fragment analysis revealed a temperature-dependent thiourea cross-link formed between 4-NCSPhe264 and *N*-terminal Pro2. Inspection of a WT crystal structure revealed that these two positions are over 30 Å apart across the dimer interface and are partially buried by neighboring loops, suggesting that thermally induced conformational changes are necessary to bring the two positions into closer contact, rationalizing why cross-linking only occurs at higher temperatures.

Enzyme stability and solvent tolerance can also be enhanced *via* ncAA-mediated cross-linking to solubilizing polymers. Deiters et al. reported the cross-linking of alkyne-derivatized polyethylene glycol (PEG) to 4-azidophenylalanine (4-AzPhe) incorporated at position 33 in superoxide dismutase.^356^ The copper-catalyzed coupling reaction yielded singly PEGylated enzyme with 70–85% conversion, and with no significant loss in activity compared to the WT. A similar approach was used to generate singly PEGylated CalB.^357^ Global substitution of the five methionine residues in the enzyme for azidohomoalanine (AzAla) yielded a variant with a single solvent-exposed azido group, as only one of the methionine residues was located on the enzyme surface. Covalent cross-linking to PEG *via* a CuAAC reaction gave a monofunctionalised species which displayed similar activity to that of the nonfunctionalized enzyme. Another study generated alkyne-decorated polymersomes, which were then cross-linked to AzAla-containing CalB.^358^ The enzyme-decorated polymersomes displayed lipase activity, and could also be recovered from the aqueous phase *via* filtration, providing a facile method of enzyme recycling. Teeuwen et al. utilized the CuAAC reaction to cross-link the same AzAla CalB variant to the alkyne group of homopropargylglycine-containing elastin-like polypeptides (ELPs), which can be used to control enzyme solubility and aggregation.^359^ The cross-linked species was purified from unreacted enzyme by taking advantage of the temperature-dependent phase transition of the ELPs, which reversibly form aggregates above a certain temperature. The residual lipase activity of the enzyme-elastin conjugate was found to be *ca*. 50% of the nonconjugated AzAla CalB variant. Enzymes containing ncAAs can also be cross-linked to solubilizing polymers using the copper-free SPAAC reaction. Debets et al. prepared dibenzocyclooctyne-functionalized PEG, which was then incubated with AzAla CalB for three hours.^360^ Full conversion to the PEGylated enzyme conjugate was achieved, with the majority of the enzyme found to di-PEGylated, suggesting that the higher reactivity associated with the strain-promoted reaction enabled cross-linking to buried as well as surface AzAla residues. In a more recent study, Wilding et al. developed a CFE screening system to assess the effects of SPAAC-mediated PEGylation at six different positions in T4 lysozyme via site-specific incorporation of 4-AzPhe.^361^ Interestingly, PEGylation efficiency did not correlate well with the solvent accessibility or hydrophobicity of each site, as previously assumed. In most positions, PEGylation led to slight increases in *T*_m_ with minimal impacts on activity. Interestingly however, PEGylation at position 91, which is located in an unstructured loop, was detrimental to both thermal stability and enzyme activity, despite this position previously being identified as optimal for lysozyme immobilization.^362^

#### 4.1.3 Immobilization

**Figure 14:**
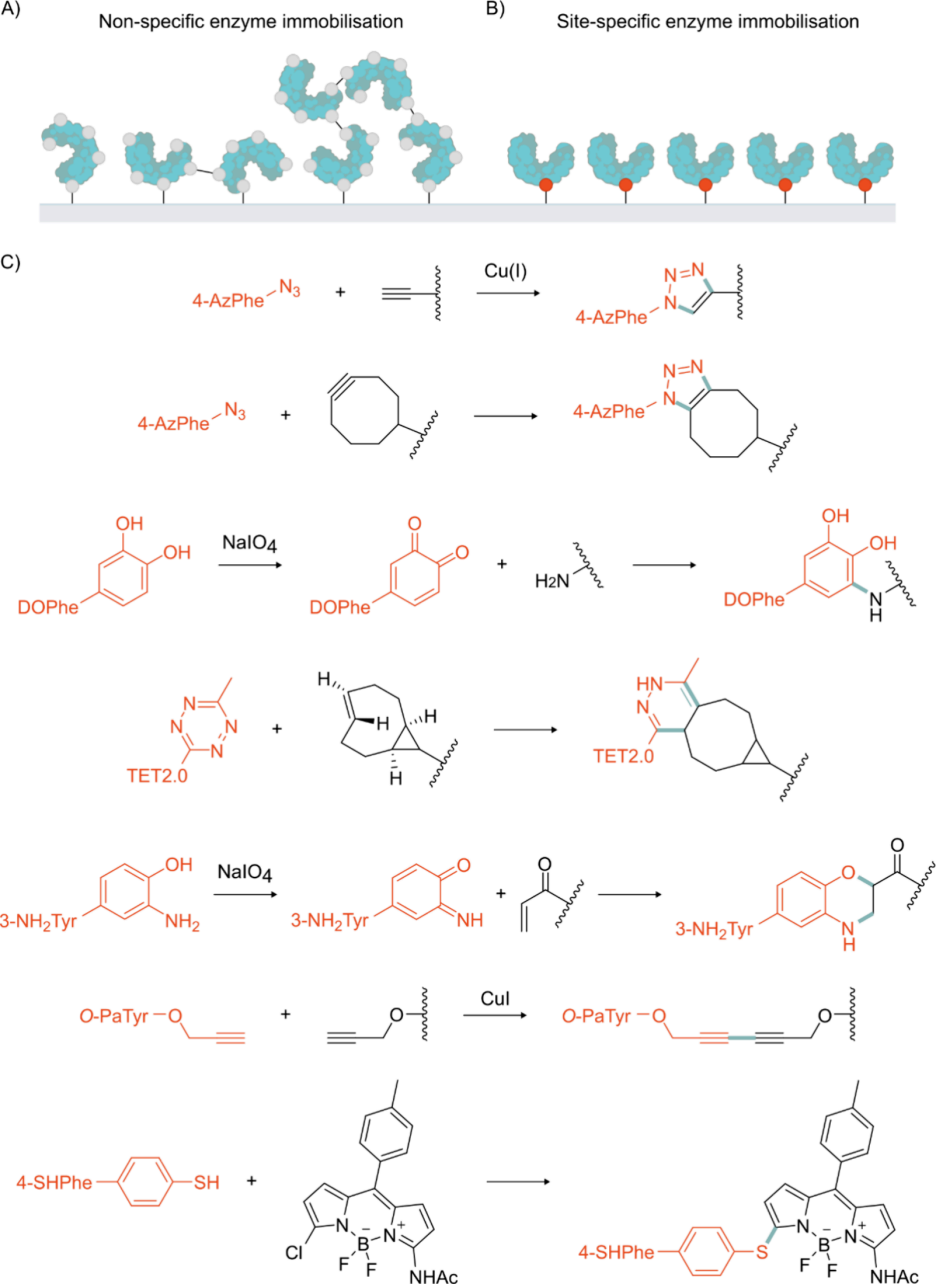
NcAA-mediated enzyme immobilization. (A) Schematic representation of nonspecific enzyme immobilization, mediated by cross-linking at multiple reactive surface residues (gray circles), resulting in multiple enzyme orientations relative to the solid support, as well as enzyme–enzyme cross-linking leading to multilayer immobilization. (B) Schematic representation of site-specific enzyme immobilization, mediated by a ncAA (orange circles) incorporated site specifically, resulting in a monolayer with a single defined enzyme orientation. (C) Immobilization chemistries utilizing ncAAs (orange). From top to bottom: CuAAC, SPAAC, DOPhe–amine coupling, tetrazine-sTCO Diels–Alder cycloaddition, 3-NH_2_Tyr-acryloyl Diels–Alder cycloaddition, Glaser–Hay alkynyl coupling, and 4-SHPhe-BODIPY coupling.

A well-established strategy for enhancing enzyme stability is *via* immobilization on a solid support.^363^ Immobilisation can increase enzyme thermostability, solvent tolerance and catalyst lifetime, as well as enabling facile biocatalyst recovery and reuse.^315^ However, nonspecific immobilization methods, such as covalent cross-linking of surface amino groups with glutaraldehyde, can substantially decrease effective activity by immobilizing the enzymes in unproductive orientations where the active site is occluded, preventing substrate access^25,364^ (Figure 14A). Site-specific ncAA incorporation enables precise immobilization of enzyme molecules in productive orientations (Figure 14B), with a range of biorthogonal cross-linking chemistries now available (Figure 14C). In some cases, ncAA-mediated immobilization can also be used to enhance enzyme activity by facilitating rapid electron exchange with functionalized electrodes.^365^

Azide-alkyne cycloadditions are an efficient method to generate biorthogonal cross-links for enzyme immobilization. Lim et al. site-specifically incorporated 4-ethynylphenylalanine (4-EthPhe) at position 43 of murine dihydrofolate reductase (mDHFR).^366^ Optimisation of the CuAAC reaction conditions enabled efficient cross-linking while maximizing residual enzyme activity. Conjugation of 4-EthPhe to a biotin-PEG3-azide linker enabled immobilization of the enzyme to a streptavidin-coated plate which displayed DHFR activity after washing, while a control immobilization where the biotin linker was omitted resulted in an inactive plate. Another study investigated the effects of different immobilization sites on T4 lysozyme activity.^362^ Site-specific incorporation of 4-propargyloxyphenylalanine (4-PaPhe) enabled enzyme immobilization onto azide-decorated magnetic beads via CuAAC. 4-PaPhe incorporation at the mouth of the active site led to a substantial 43% reduction in activity upon immobilization, whereas incorporation at more distal sites only resulted in 19–24% reductions. Additionally, one of these variants, Leu91 4-PaPhe, exhibited higher activity than the WT enzyme immobilized *via* nonspecific epoxy cross-linking. Enzyme lifetime was also improved, with the immobilized Leu91 4-PaPhe variant retaining 79% activity after three freeze–thaw cycles, compared to 42% residual activity for the epoxy-immobilized WT, and < 27% for nonimmobilized controls. A comparable trend was observed after incubation in 2 M urea. In a similar study, Wang et al. individually substituted five Tyr residues for 4-AzPhe in *Geobacillus sp*. lipase and immobilized the mutants on a cyclooctyne-decorated support *via* SPAAC.^367^ Triolein hydrolysis activity of the mutants increased by 5–37% upon immobilization, apart from a variant cross-linked at a position adjacent to the active site, which exhibited a slight decrease in activity. All of the immobilized mutants compared favorably with the glutaraldehyde-immobilized WT, displaying up to 6.8-fold higher activity. Additionally, the SPAAC-linked mutants were modestly more thermostable than the randomly cross-linked WT, with up to 1.2-fold higher residual activity recorded after a 29 h incubation at 50 °C. Li et al. also utilized 4-AzPhe, immobilizing aldehyde ketone reductase (AKR) variants to a bicyclononyne-functionalized resin.^368^ Variants containing 4-AzPhe at one of five positions were tested, all of which displayed 5–16% increased activity upon immobilization and were also more thermostable than the free unmodified enzyme. Impressively, combining all five 4-AzPhe substitutions into one variant enhanced thermostability further, with over 70% residual activity recorded after 24 h incubation at 60 °C, compared to 5% for the free enzyme.

Other biorthogonal chemistries can also be used to immobilize ncAA-substituted enzymes. DOPhe can be oxidized with sodium periodate to form an orthoquinone intermediate, which can then react with an amino-functionalized coupling partner.^369^ Deepankumar and co-workers site-specifically incorporated DOPhe into an ω-transaminase and demonstrated successful immobilization on chitosan and polystyrene beads.^370^ They combined this approach with global incorporation of 4-*R*-FPro, which substantially increased thermostability, doubling the half-life of the enzyme at 70 °C, as well as modestly improving tolerance of a range of organic solvents. Immobilisation of the stabilized variant reduced activity by less than 10% and enabled efficient biocatalyst recovery, with almost complete preservation of enzyme activity following 10 cycles of a 12 h kinetic resolution process. Another study utilized the inverse-electron-demand Diels–Alder (IEDDA) reaction between 1,2,4,5-tetrazines and strained *trans*-cyclooctenes (sTCOs) for immobilization of thermostable carbonic anhydrase II (tsCA).^371^ This conjugation strategy was chosen for its high rate constant (∼72,000 M^–1^ s^–1^) which allowed a reduction in immobilization time, thereby minimizing nonspecific adsorption to the solid support. A previously developed tetrazine-containing ncAA, Tet2.0,^372^ was incorporated into tsCA separately at three surface positions using SCS, chosen to orient the active site toward bulk solvent, toward the solid support, or parallel to the support. The cross-linking reaction between these variants and sTCO-decorated magnetic beads was found to be efficient enough to allow substoichiometric quantities of enzyme to be used, enabling precise control of the amount of enzyme immobilized on the beads. The orientation of the immobilized enzyme was found to control hydrolysis activity, with the solvent-facing variant retaining 90% activity compared to preimmobilization, the parallel-facing variant 75%, and the support-facing variant 60%. The researchers also demonstrated successful enzyme-limited immobilization onto an sTCO-functionalized flat surface, demonstrating the versatility of the technique. Switzer et al. used Glaser-Hay alkynyl coupling to immobilize a 4-PaPhe-substituted carboxylesterase to an alkyne-decorated Sepharose resin.^373^ Four surface Tyr residues were identified and individually substituted with 4-PaPhe, with immobilization of all four variants yielding catalytically active resins. Solvent tolerance was greatly improved by immobilization, with the most active variant displaying increased activity in neat THF compared to neat aqueous buffer, whereas activity of the free WT enzyme was reduced by over 60% in a 1:1 mixture of buffer and THF. The immobilized carboxylesterase also displayed impressive recyclability and lifetime, with almost no activity lost after three years of storage and 18 reaction cycles, compared to the free WT which only retained 20% activity after 6 months of storage.

NcAA-mediated immobilization can also enhance direct electron transfer (DET) between redox enzymes and electrodes, enabling more efficient electrocatalysis, current generation, and reaction monitoring. Ray et al. site-specifically incorporated 3-aminotyrosine (3-NH_2_Tyr) at a surface position in Mb, which was used to immobilize the protein on an acryloyl-derivatized gold electrode via oxidation of the ncAA to the 2-iminoquinone and subsequent Diels–Alder cycloaddition.^374^ As a control, WT Mb was randomly immobilized using a carbodiimide reagent. Atomic force microscopy (AFM) characterization revealed that the ncAA-immobilized protein formed a monolayer with an average feature height of 5 nm, whereas the WT was immobilized in an uneven, multilayer arrangement with an average feature height of 175 nm. Cyclic voltammetry measurements indicated DET could occur between the electrode and both forms of immobilized Mb. Electrocatalytic oxidation of thioanisole was then investigated, with randomly immobilized WT Mb exhibiting 87% conversion and the 3-NH_2_Tyr-immobilized Mb a comparable 81%, despite the far fewer number of enzyme molecules immobilized in the monolayer arrangement. A different study utilized the SPAAC reaction to immobilize 4-AzPhe-containing mutants of a small laccase to cyclooctyne-derivatized carbon nanotubes.^365^ During electro-biocatalytic oxidation of 2,6-dimethoxyphenol, an electron transfer efficiency of 28.7% was recorded when 4-AzPhe was incorporated at position 47, compared to 1.9–7.5% at three other surface positions, and 1.3–3.4% when the enzyme was randomly immobilized by cross-linking surface amino groups to succinimidyl ester-decorated nanotubes. The success of this immobilization position was attributed to its proximity to a water channel connecting the catalytic copper cluster in the active site with the enzyme surface, which may have facilitated electron transfer to the electrode. The 4-AzPhe-immobilized enzyme also exhibited a longer lifetime, with catalytic current decreasing by only 13.6% after 8 days of storage at room temperature, compared to 47.0% for the randomly immobilized control. In another study, Xia and co-workers developed an orthogonal aaRS to enable site-selective incorporation of 4-thiolphenylalanine (4-SHPhe) into tryptophan oxidase (TrpOx).^375^ This allowed the enzyme to be derivatized with a boron-dipyrromethene (BODIPY) moiety *via* a nucleophilic substitution reaction with the ncAA thiol. BODIPY molecules can bind to carbon nanotubes by forming strong π-π interactions, enabling immobilization of BODIPY-conjugated enzymes. AFM characterization of nanotubes incubated with 4-SHPhe-modified enzyme revealed 51.5% coverage when BODIPY was present and only 12.3% when it was absent, with the residual coverage attributed to nonspecific adsorption. On addition of the substrate tryptophan, an oxidative current was detected only from the electrode incubated with both BODIPY and enzyme, demonstrating the immobilized enzyme was functional and that site-specific immobilization was required for efficient electron transfer.

### 4.2 Improving Enzyme Selectivity

The introduction of ncAAs into enzymes can be used to reshape substrate binding pockets, leading to altered substrate profiles, product distributions and stereoselectivities.

#### 4.2.1 Altering Substrate Profiles

**Figure 15:**
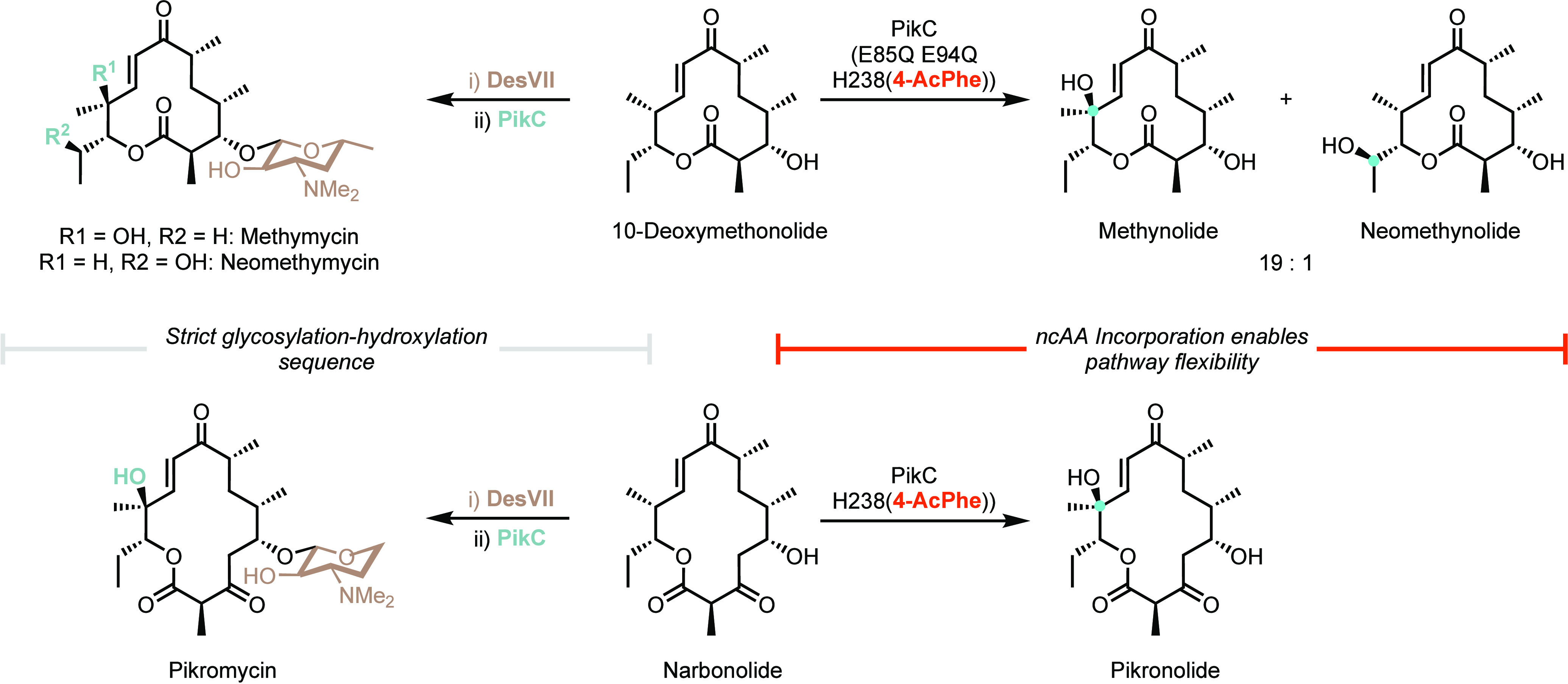
Introduction of 4-AcPhe into PikC, a CYP450 enzyme, enabled biosynthetic reprogramming through allowing C(sp^3^)–H oxidation to occur in the absence of an amino-sugar moiety (brown).

The introduction of ncAAs has been used to alter the substrate preference of PikC, a P450 enzyme that catalyzes a C(sp^3^)–H oxidation step in the biosynthesis of macrolide antibiotics.^376^ PikC substrates require an amino-sugar motif appended during the previous biosynthetic step by the glycosyltransferase DesVII, but it was demonstrated that installation of an ncAA could alleviate this requirement (Figure 15). Surveying a range of aromatic ncAAs at several positions around the substrate binding pocket revealed the mutation His238(4-AcPhe) enabled the site-selective hydroxylation of two different aglycone substrates (10-deoxymethonolide and narbonolide), differentiated from their respective final antibiotic by only the amino-sugar moiety (Figure 15, right). Oxidation site-selectivity for 10-deoxymethonolide could be improved further to 19:1 (methynolide:neomethynolide) by transplanting two known mutations (Glu85Gln and Glu94Gln) into the His238(4-AcPhe) construct. By contrast, the WT PikC oxidizes the glycosylated analogue of 10-deoxymethonolide in a ∼ 1:1 ratio. Structural analyses and docking studies suggested the changes in substrate specificity induced by 4-AcPhe incorporation were due to the acetyl motif supplying hydrogen-bonding and hydrophobic interactions that are normally provided by the desosamine motif in the native substrates. Additional hydrogen-bonding interactions were observed between the C5-hydroxyl group in narbonolide and the acetyl group. Interestingly, the His238(4-AcPhe) mutation also led to improvements in activity and site-selectivity toward the native substrate. The ability to reprogram biosynthetic sequences by altering substrate specificities could have applications in structural diversification for analogue discovery and enabling combinatorial biosynthesis.

#### 4.2.2 Altering Product Distributions

**Figure 16:**
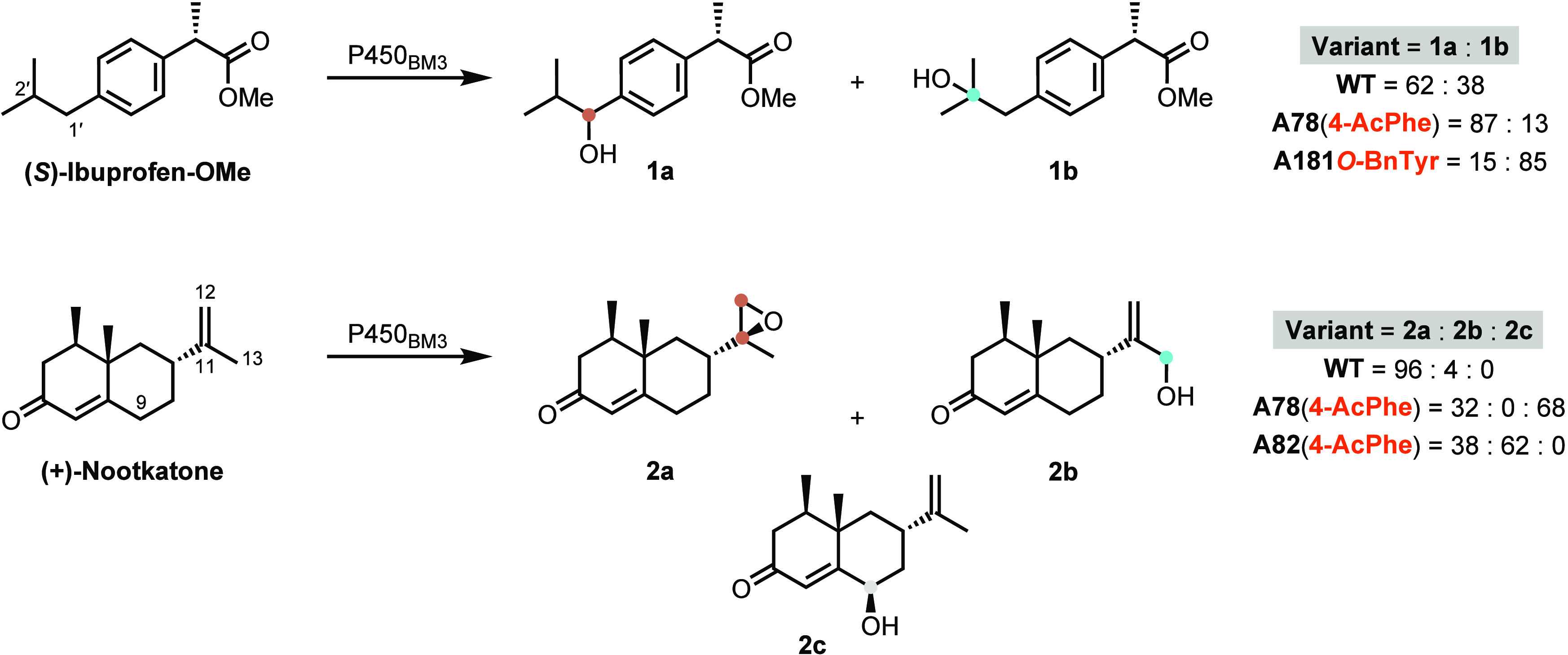
Incorporation of ncAAs at various positions within P450_BM3_ alters the oxidation product distributions for (S)-ibuprofen-OMe and (+)-nootkatone substrates.

Fasan and co-workers have investigated the effect of installing ncAAs into a P450 on oxidation regioselectivity.^377^ Significant changes in oxidation product distribution were achieved when 4-AcPhe or *O*-BnTyr were incorporated at one of eleven positions located around the active site or proximal to the prosthetic heme ligand in P450_BM3_ (Figure 16). For (*S*)-ibuprofen methyl ester, the Ala78(4-AcPhe) mutation shifted the oxidation product ratio further toward the C1′ oxidation product (**1a**), whereas Ala181*O*-BnTyr led to a reversal in regioselectivity, furnishing C2′ oxidation product (**1b**) as the major species (15:85 **1a**:**1b**). For (+)-nootkatone, a new oxidation product, **2c**, that was not formed by the WT enzyme was observed; the Ala78(4-AcPhe) mutant formed this as the major product (32:68 **2a**:**2c**). The Ala82(4-AcPhe) mutant reduced the native epoxidation activity and concomitantly increased C(sp^3^)–H oxidation activity; **2a** and **2b** were formed in a 38:62 ratio respectively. In these cases, canonical substitutions to Tyr at positions 78 and 181 did not give rise to altered product distributions, demonstrating how substrate binding pockets can be more extensively remodelled using ncAAs compared with using cAAs alone on the regioselectivity of P450 oxidations.^377^

#### 4.2.3 Improving Stereoselectivity

NcAA incorporation has been used to improve the stereoselectivity of *Acinetobacter baylyi* DKR.^378^ The WT DKR used in this study catalyzes the reduction of 2-chloro-2-phenylethanone with modest selectivity (9% *ee*) in favor of the (*R*)-enantiomer. Mutating Trp222 to smaller cAAs led to a switch in enantio-preference to the (*S*)-enantiomer, suggesting that steric bulk at position 222 could be a determinant of facial selectivity for hydride delivery. A number of ncAAs with bulky aromatic side chains were incorporated at this position, with the largest improvement in stereoselectivity achieved with *O*-*t*BuTyr (up to 34% *ee*), the bulkiest ncAA tested.

An expanded genetic code has also been used to improve the diastereoselectivity of *Pseudomonas alcaligenes* lipase during menthol propionate hydrolysis through enhancing substrate specificity for L-menthol propionate over seven other stereoisomers present in the mixture.^379^ Guided by MD simulations, a number of substituted aromatic ncAAs were installed at different positions around the active site. The most improved mutant identified was Ala253(2-BrPhe) which selectively catalyzed L-menthol propionate hydrolysis with 94% conversion and > 95% diastereomer selectivity. In another study, Kourist and co-workers investigated the effect of five different ncAAs at ten positions around the substrate binding pocket of the hydrolase *Pseudomonas fluorescens* esterase.^380^ A split-GFP assay was employed to rapidly assess folding of the fifty possible variants, leading to the identification of several sites that are intolerant of ncAA incorporation. The activity and selectivity of the folded, soluble variants were assessed in the kinetic resolution of ethyl 3-phenylbutyrate. While conversions were lower than those achieved with the WT enzyme, enantioselectivity was improved from 27 to 68% *ee* with a Phe162(2-NapAla) variant, while an Ile224(4-AzPhe) substitution led to a switch in enantio-preference.

### 4.3 Improving Kinetic Parameters

In addition to improving enzyme stability and selectivity, the introduction of one or multiple ncAAs has been shown to improve enzyme kinetic parameters in effects not replicable with cAAs. Substitutions using both SPI and GCE have been shown to increase *k*_cat_, increase total turnover number (TTN), and improve substrate binding.

#### 4.3.1 Noncanonical Ligands in Artificial Metalloenzymes

**Figure 17:**
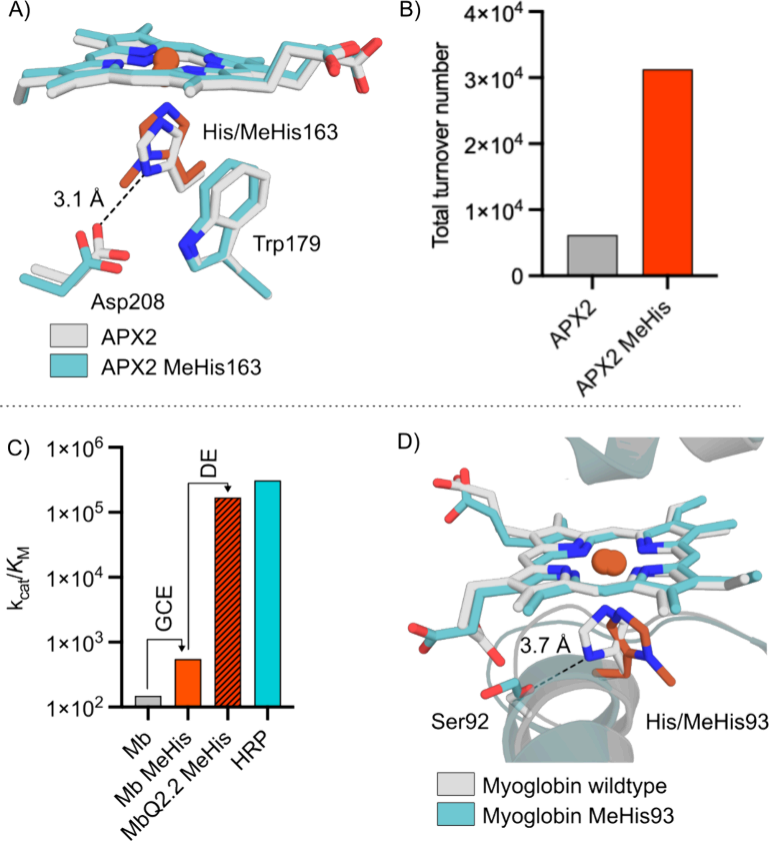
Peroxidases with MeHis proximal ligands. (A) An overlay of the crystal structures of APX2 (PDB: 1OAG(382)) and APX2 MeHis163 (PDB: 5L86(381)). Key active site residues and the heme are shown as atom-colored sticks with gray and blue carbons, respectively. MeHis is shown with brown carbons. (B) TTN achieved by APX2 and APX2 MeHis. (C) The catalytic efficiency toward guaiacol (2-methoxyphenol) oxidation for Mb variants and horseradish peroxidase (HRP). (D) An overlay of the crystal structures of Mb (PDB: 1A6K(383)) and Mb MeHis93 (PDB: 5OJ9(384)). The protein backbones are shown as cartoons, and key active site residues and the heme are shown as atom-colored sticks with gray and blue carbons, respectively. MeHis93 is shown with brown carbon atoms.

Heme peroxidases employ a conserved His residue as their axial iron ligand, which forms a strong hydrogen bond from the γ N–H to a neighboring aspartate, an interaction known to increase the electron donating capability of His (see section 3.3.4). Introduction of 3-methylhistidine (MeHis) by GCE into the axial position of an engineered ascorbate peroxidase (APX2) disrupts this hydrogen bond and surprisingly leads to a modest increase in the catalytic efficiency of guaiacol (2-methoxyphenol) oxidation (Figure 17A).^381^ Remarkably, this structurally conservative substitution also greatly increases the TTN achieved prior to enzyme deactivation (31,300 and 6,200 for APX2 MeHis and APX2, respectively, Figure 17B).

The oxygen binding protein Mb lacks the proximal pocket aspartate, which is thought to be one contributing factor to the weak peroxidase activity displayed by this protein.^385^ Inspired by the improved catalytic activity of APX2, the proximal ligand (His93) in sperm whale Mb was mutated to MeHis, causing a rotation of the imidazole plane (Figure 17D) and a ∼ 4-fold increase in the *k*_cat_ of guaiacol oxidation (1.4 vs 6.6 s^–1^).^384^ This increased activity was further improved by both rational mutation and directed evolution, to give a final variant with a catalytic efficiency for guaiacol oxidation that is 1,140-fold higher than WT Mb and in the order of natural peroxidases (Figure 17C).

**Figure 18:**
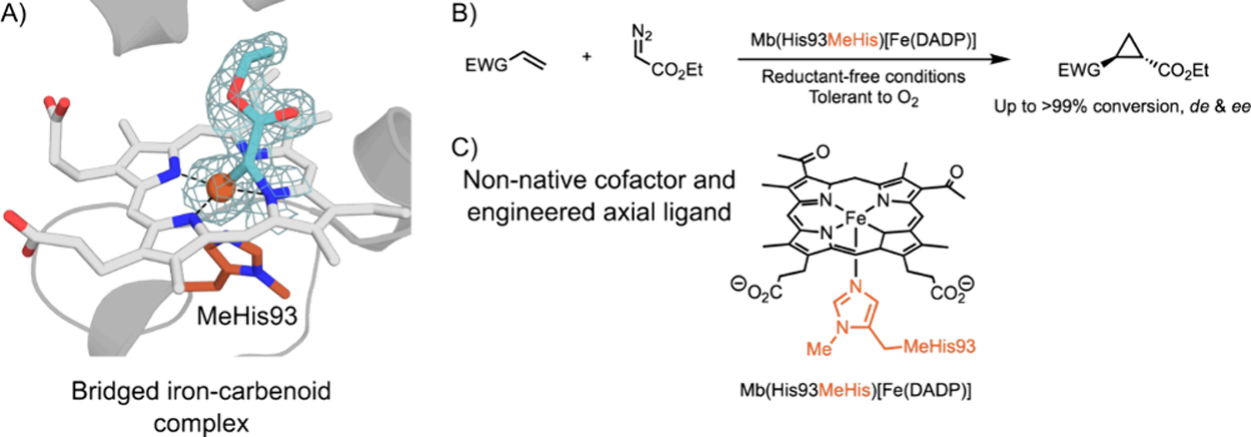
Biocatalytic cyclopropanations by Mb* MeHis93. (A) The bridged ion carbenoid intermediate observed by X-ray crystallography (PDB: 6F17(386)). A 2F_O_–F_C_ map contoured at 1.5 σ is shown around the bridged carbenoid intermediate and the iron atom. (B) The cyclopropanation reaction catalyzed by engineered Mbs. (C) The non-native cofactor and MeHis ligand used to expand the scope of biocatalytic cyclopropanations.^388^

Axial ligand substitutions in Mb have also been shown to improve nonbiological carbene transfer chemistry. Mutation of the axial iron binding His93 to MeHis in an engineered Mb (Mb*) obviates the requirement for a priming reductant and allows for efficient carbene transfer under aerobic conditions, which contrasts with the oxygen sensitivity of the His93-containing Mb*.^386^ These properties were attributed to increased electrophilicity of the Fe(III) center, promoting direct attack of the substrate ethyl diazoacetate (EDA) to produce the carbenoid adduct. This intermediate was captured and characterized through X-ray crystallography and found to form an “unusual” bridging Fe(III)–C–N(pyrrole) geometry (Figure 18A), that the authors suggest is a reaction intermediate in Mb* MeHis93 and in the His-containing enzyme.^386^

Pott et al. described the introduction of a wider range of noncanonical ligands into Mb, namely 5-thiazoylalanine (5-ThzAla), 4-thiazoylalanine (4-ThzAla) and 3-(3-thienyl)alanine (3-ThiAla), and found both Mb*(MeHis) and Mb*(5-ThzAla) led to improved oxygen tolerance *cf*. Mb*.^387^ This augmented activity extended to abiological N–H insertion reactions, with MeHis93 and 5-ThzAla93. Introduction of 4-ThzAla as a ligand in Mb*, was found to give the most effective catalyst for S-H insertion reaction of EDA and thiophenol, plausibly due to the higher redox potential of the heme increasing the rate of radical recombination of the intermediate thiol radical with the iron-carbenoid.

Noncanonical ligand substitutions have also been used to expand the scope of biocatalytic cyclopropanations. Installing a non-native iron porphyrin cofactor and a noncanonical axial MeHis ligand into the Mb* variant of Mb afforded a selective artificial metalloenzyme that could catalyze cyclopropanation reactions with electron deficient alkenes (Figure 18B,C).^388^ Mechanistic studies indicate that this expanded scope is enabled by radical-type carbene transfer reactivity from the combined effect of the non-native cofactor and axial ligand. In a subsequent study, a wider range of noncanonical ligands were explored at position 93, including 4-NH_2_Phe and 3-(3′-pyridyl)-Ala (3-PyrAla), albeit with reductions in TTN and/or selectivity compared to the MeHis-containing variant.^389^

Replacement of one key His ligand (His264) in the zinc metalloenzyme mannose-6-phosphate isomerase (ManA) by MeHis gave a functional enzyme that permitted growth of an *E. coli* strain lacking endogenous ManA activity, albeit at a lower growth rate.^390^ Directed evolution of ManA His264MeHis led to the generation of an organism whose growth was strictly dependent on the presence of ncAA, providing a possible strategy for the biocontainment of recombinant organisms.

NcAAs have also been introduced to the distal pocket of heme proteins to tune the properties of the heme cofactor and augment catalytic function. Mutation of the distal pocket His64 of Mb to DOPhe led to a 54- and 10-fold increase in catalytic rate toward benzaldehyde oxidation and thioanisole sulfoxidation, respectively.^391^ These improvements were proposed to arise from increased stabilization of the ferryl intermediate compound I from additional hydrogen bonding interactions in the His64DOPhe mutant, with the ncAA performing the hydrogen bonding role of the distal pocket His/Arg residues that are conserved in natural peroxidases.

#### 4.3.2 Noncanonical Amino Acids to Tune Enzyme–Substrate Interactions

A range of cAAs and ncAAs were introduced into the Phe124 position of the prodrug activator nitroreductase, and the activity toward the prodrug CB1954 was found to increase 30-fold with 4-NO_2_Phe.^392^ This residue forms π-stacking interactions with the aryl ring of the prodrug substrate, and previous studies interrogating this position had identified its importance to activity.^393^ Interestingly, no correlation between ring electron density and catalytic rate was observed. Instead, it was hypothesized that a polarized aromatic ring at position 124 aids in π-stacking with the polarized aromatic substrate.

Mutation of the active site residue Tyr309 in a bacterial PTE to Hco increased the rate of hydrolysis of the insecticide paraoxon (∼8-fold improvement in *k*_cat_).^394^ This improvement was suggested to arise from electrostatic repulsion between the negatively charged 4-nitrophenolate product and the anionic ncAA side chain to facilitate the rate-limiting product release step.

#### 4.3.3 Noncanonical Amino Acids to Introduce Conformational Changes

Conformational changes resulting from ncAA incorporation have been reported to augment biocatalyst properties by stabilizing structural elements, improving substrate packing or increasing the rate of product release. Pagar et al. described the systematic incorporation of three ncAAs, 4-MePhe, 4-CF_3_Phe, and BpAla, into each of the positions 33, 86, and 88 in an (*R*)-selective transaminase, (*R*)-ATA,^395^ which was developed for industrial sitagliptin production.^396^ Phe88(4-MePhe), Phe88(4-CF_3_Phe), and Phe88BpAla variants all showed increased catalytic activity, the most active variant contained BpAla and gave a 15-fold improvement in activity with 1-phenylpropan-1-amine. Introduction of a Phe86Ala mutation into the BpAla containing variant afforded a Phe86Ala/Phe88BpAla double mutant with 30% higher residual activity after 55 °C incubation, which was used to perform kinetic resolution of 1-phenylpropan-1-amine with benzaldehyde as an amine acceptor.

In another study, the *k*_cat_ of a transketolase engineered to accept aromatic aldehydes was improved by 2-fold with a Tyr385(4-CNPhe) substitution, with reduced substrate inhibition.^397^ An alternative substitution, Tyr385(4-NH_2_Phe), increased the *T*_m_ by 5 °C. Structural modeling suggested this was due to the stabilization of an active site helix through an intersubunit hydrogen bond with Gly262.

SPI of 5-FTrp in place of four canonical Trp residues within the M1–1 isoenzyme of rat GST led to a 4-fold increase in *k*_cat_ for the arylation of glutathione using 1-chloro-2,4-dinitrobenzene as a substrate, a reaction that is thought to be limited by the rate of product release.^398^ Introduction of the noncanonical residues were shown by X-ray crystallography to introduce subtle conformational disorder into the active site, plausibly increasing the rate of product release. Interestingly, the *k*_cat_ was unchanged using phenanthrene 9,10-oxide and 4-phenyl-3-buten-2-one as substrates, where the chemical step is rate limiting.

SPI was also used to replace the four native Phe residues in the restriction endonucleause PvuII with 2-FPhe, 3-FPhe, or 4-FPhe.^399^ Of the three fluorinated Phe analogues, only 3-FPhe increased activity, by approximately 2-fold compared with the WT enzyme. The mutated residues were located distal to the catalytic center and DNA binding sites, with the observed activity increases attributed to conformational changes upon ncAA incorporation.

The specific activity of the lipase TTL toward 4-nitrophenyl palmitate hydrolysis increased upon substitution of 11 methionine residues with norleucine (Nle) using SPI. Activity increases were also observed upon substitution of Asp221 with BpAla by GCE.^400^ Notably, simultaneous incorporation of both Nle and BpAla into TTL also gave an engineered variant with higher activity than the WT, albeit with slightly reduced activity compared with TTL containing only the Met to Nle substitutions. Similarly, SPI of Nle into 13 Met residue positions in the heme domain of cytohrome P450_BM3_ increased the rate of peroxygenase catalysis ∼ 2-fold.^401^

In a modified directed evolution experiment, ten ncAAs Phe analogues were introduced at random positions within TEM-1 β-lactamase using SCS.^402^ The resulting library were evaluated using a cell viability assay, leading to identification of a Val216(4-acrylamido-phenylalanine) (4-AcrPhe) variant that increased *k*_cat_ 14-fold with only a slightly increase in *K*_M_. Structural analysis of both apo enzymes and cephalexin acyl-enzyme intermediates by X-ray crystallography revealed conformational changes to key active site residues that likely lower the barrier to transacylation in the ncAA containing mutant.

#### 4.3.4 Building Artificial Dimers Through Noncanonical Amino Acid Tethering

**Figure 19:**
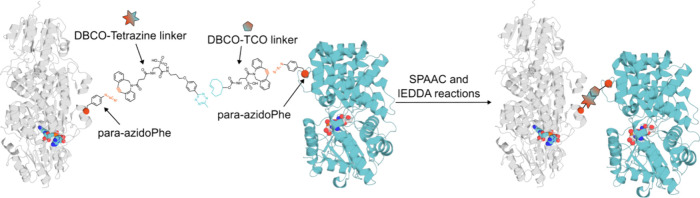
Introduction of 4-AzPhe into selected sites of formate dehydrogenase (FDH) and mannitol dehydrogenase (MNDH) created bioorthogonal handles for SPAAC conjugation to either a heterobifunctional linker harboring a tetrazine handle or an alternative linker with a cyclooctene handle (PDB: 3WR5,^407^1LJ8^408^). FDH and MNDH are shown as gray and blue cartoons, respectively. The sites of 4-AzPhe incorporation are shown as red spheres.

Genetic incorporation of the ncAA 4-AzPhe^403^ introduces a ‘clickable’ functional group into proteins, which has been used to form artificial heterodimers using SPAAC reactions with bifunctional linkers.^404^ Lim et al. used this approach to improve the cofactor shuttling and increase the efficiency of a two-enzyme cascade for *D*-mannitol production.^405^ Introduction of 4-AzPhe into selected sites of formate dehydrogenase and mannitol dehydrogenase (MNDH) created bioorthogonal handles for SPAAC conjugation to either a heterobifunctional linker harboring a tetrazine handle, or an alternative linker with a cyclooctene handle. The tetrazine and cyclooctene moieties could then undergo an IEDDA reaction to give the artificial heterodimer, with the spatial relationship between the two proteins defined by the site of ncAA incorporation and the length of the linkers (Figure 19). Subsequent work described how the relative orientation of the two enzymes’ active sites affected catalysis.^406^ With the active sites in closer proximity, *D*-mannitol production was 60% higher in comparison to the artificial heterodimer with active sites orientated further from one another.

### 4.4 Regulation of Enzyme Activity

NcAAs have been used to install artificial regulatory elements into biocatalysts to enable spatiotemporal control over enzyme activity *in vivo* or *in vitro*.

#### 4.4.1 Chemical and Photochemical Decaging

Genetic code expansion has been used to install protected analogues of functional amino acids into enzymes. These caged amino acids offer an effective strategy to inhibit enzyme activity, either through the steric hindrance of ligand binding sites or by masking key catalytic groups, with facile restoration of activity achievable through cleavage of the obstructing group using chemical^409−411^ or photochemical^412−424^ methods.

Light offers a minimally invasive stimulus that can be used to regulate protein function both *in vitro* and *in vivo*. The most frequently employed photocaging moieties are based upon *O*-nitrobenzyl (*O*-NB) groups, which can be applied to cap hydroxy, carboxy, thiol or amino groups and are easily cleaved upon irradiation with long-wave UV light.^425,426^ Chemical methods including both SPPS and the PTM of amino acid functional groups have proven effective in the photocaging of proteins. However, these techniques are usually restricted to smaller peptides or preferentially target reactive surface residues. One such example involved the photocaging of lysozyme through the reaction of solvent exposed Lys side chains with a photocleavable PEG reagent.^412^ PEGylation of the enzyme resulted in a surrounding polymer layer that prevented interaction with substrate and inhibited enzyme activity, which could only be restored upon irradiation-induced cleavage of the bound PEG polymers.

**Figure 20:**
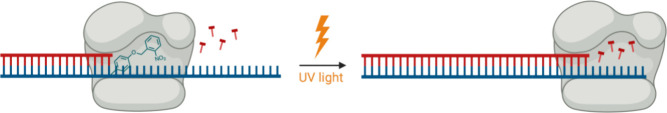
Introduction of a photocaged ncAA into a DNA polymerase through GCE occludes the active site, preventing the diffusion of nucleotides for extension. Brief irradiation with UV light cleaves the O-NB moiety to reveal the catalytic Tyr and restore polymerase activity. Created with BioRender.com.

Alternatively, GCE provides a more targeted approach to install new regulatory elements, and has been used to incorporate photocaged analogues of Tyr, Lys, Ser and Cys into enzyme active sites.^413−424^ An early example from the Schultz lab reported the replacement of an active site Tyr503 with *O*-NBTyr in β-galactosidase to afford an inhibited variant that could be activated upon irradiation with 365 nm light to recover 67% of WT activity.^414^ This approach was extended to the development of orthogonal translation components for incorporating photocaged tyrosine analogues in eukaryotic hosts,^427^ which has enabled photochemical control of enzymes such Cre recombinases,^428^ proteases,^422^ or nucleases.^417^ Zinc-finger nucleases (ZFNs) have been developed for the sequence-specific scission of double-stranded DNA to enable facile gene editing.^429−432^*O*-NBTyr was installed into ZFN to develop a photochemically activatable restriction enzyme.^417^ Rational replacement of active site Tyr471 by *O*-NBTyr occludes the active site, preventing binding of the DNA substrate, meaning that phosphodiester cleavage was only observed after brief irradiation with 365 nm light. Crucially, this photocaging strategy is entirely compatible with further engineering of the ZFN, allowing for additional modifications to the dimer interface and introduction of additional mutations to further augment nuclease activity.^433^ Photocaged analogues of Tyr have also enabled the regulation of polymerase activity. In the widely used *Thermus aquaticus* (*Taq*) polymerase, Tyr671 plays an important role in positioning the DNA template and the incoming dNTP.^434,435^ Replacement of Tyr671 with *O*-NBTyr led to an inactivated variant of *Taq* polymerase, where the bulky *O*-NB group is believed to occupy dNTP binding cavity (Figure 20). Upon release of the *O*-NB caging group via short irradiation with 365 nm light, polymerase activity was restored to 71% of that of the WT.^415^ This photocaged polymerase enabled development of a UV-inducible hot-start PCR method, a strategy which can reduce nonspecific DNA amplification and increase sensitivity and specificity.^436^ Since various monomeric DNA and RNA polymerases also rely on an analogous active site Tyr,^437^ recombinant replacement of this residue with *O*-NBTyr could provide a general approach to photochemical regulation of polymerase activity in these enzymes. To illustrate this broader applicability, Chou et al. engineered a photocaged variant of a bacteriophage T7 RNA polymerase (T7RNAP) with Tyr639 substituted with *O*-NBTyr.^416^ As T7RNAP is orthogonal to all endogenous prokaryotic and eukaryotic RNA polymerases, expression of genes of interest under T7 promoters in bacterial and mammalian cells could be precisely controlled *via* light activation. Spatiotemporal activation of the polymerase was demonstrated through the engineering of *E. coli* to produce the photocaged T7RNAP alongside either GFP or luciferase reporter genes. In both cases, light irradiation successfully initiated RNA polymerization leading to downstream gene expression, where fluorescence intensity correlated with the duration of UV exposure. A more recent study targeted Lys631 for genetic replacement with methyl-2-nitropiperonyllysine (MNPLys), producing an additional light activatable variant of T7RNAP.^420^

CRISPR/Cas9 is a powerful technology for genome editing that is widely used in medicine and biotechnology. The Deiters lab sought to add an optically controllable element to the Cas9 enzyme using GCE.^421^ A combination of Ala and ncAA scanning experiments revealed the catalytic importance of Lys886 for Cas9 activity, so this residue was targeted for substitution with a photocaged analogue using SCS. The presence of MNPLys in the protein active site abolished DNA cleavage and nicking activity. Enzyme activity could be fully restored upon photodecaging of Lys886 using 365 nm light. This light-activated Cas9 system was successfully used for endogenous gene silencing, leading to reduced expression of the CD71 gene and 50% reduction of the CD71 transmembrane transferrin receptor on the cell surface.

Photocaged ncAAs have also found application for studying and modulating kinase activity. MNPLys was incorporated in place of the near-universally conserved Lys97 in the ATP binding domain of the MAP kinase MEK1, inhibiting its activity by blocking ATP binding and preventing phosphorylation.^419^ Following photodecaging of Lys97, receptor-independent light activation of a designed subnetwork of the Raf/MEK/ERK signaling pathway was achieved in live mammalian cells, enabling study of the kinetics of individual steps in the transduction cascade.

The chemiluminescence activity of *Renilla* luciferase (RLuc) can also be controlled directly through the genetic replacement of a catalytic Cys residue with the photocaged analogue PCys using a previously engineered *Mb*PylCKRS.^423,127^ Following successful incorporation of the caged Cys, the activity of RLuc could be initiated with brief UV irradiation resulting in a > 150-fold increase in chemiluminescence activity over the caged enzyme. Protease activity can also be regulated via photocaging of catalytic Cys nucleophiles. An early study achieved genetic incorporation of *O*-NBCys in place of the active site Cys163 of capase-3 in yeast.^413^ In lysate assays of the mutant enzyme, protease activity was only detectable after photodecaging upon irradiation with UV light. Interestingly, while expression of WT capase is toxic to yeast cells, expression of the photocaged variant was not detrimental to cell growth. This methodology was extended to protease expression in *E. coli* through the engineering of a PylRS/tRNA_CUA_ pair to encode photocaged Cys variants, allowing the development of light-activatable TEV protease variant.^422^

#### 4.4.2 Azobenzene Photoswitches

**Figure 21:**
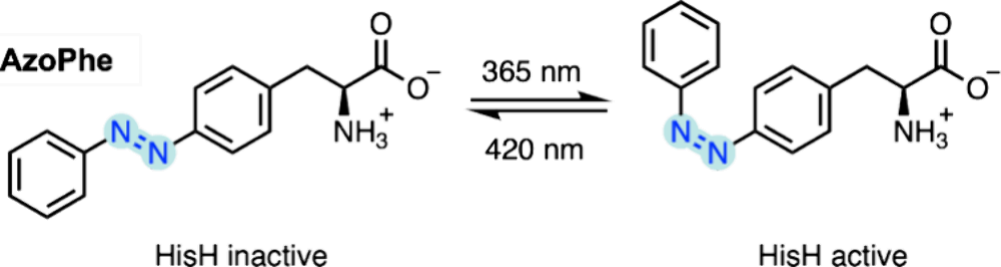
Photoresponsive ncAAs used in the allosteric light regulation of ImGPS. AzoPhe undergoes light induced reversible *E/Z* isomerizations enabling on–off switching of HisH activity.

Azobenzene derivatives have found diverse application in the reversible photocontrol of biomolecules, owing to their efficient light-induced *E/Z* isomerization which result in structurally disparate isomers (Figure 21).^438−447^ Notably, a variety of azobenzene-based photoswitches can now be selectively incorporated into proteins using GCE.^448−456^ In one study, incorporation of an azobenzene switch enabled photochemical control over an allosteric activation mechanism in imidazole glycerol phosphate synthase (ImGPS).^453^ The HisH glutaminase subunit of this bienzyme complex is allosterically stimulated by binding of a regulatory ribonucleotide to the HisF cyclase subunit. Light-mediated isomerization (356 nm to induce *E* to *Z* isomerization and 420 nm for *Z* to *E*) of 4-azobenzylphenylalanine (AzoPhe) introduced at position 55 of HisF resulted in reversible 10-fold regulation of HisH activity. In another study, AzoPhe, 4-azo-(2’,6’-difluorobenzyl)phenylalanine (F_2_AzoPhe), and 4-azo-(2’,6’-difluorobenzyl)-3,5-difluorophenylalanine (F_4_AzoPhe) were incorporated into firefly luciferase (FLuc) in both prokaryotic and eukaryotic cells.^454^ Unsubstituted AzoPhe demonstrates UV light-induced *E* to *Z* photoswitching but spontaneously reverts to the more stable *E*-isomer, posing challenges for long-term control of protein function. In contrast, F_4_AzoPhe possesses enhanced thermal stability in the *Z* state. Furthermore, the fluorine substituents lead to a red-shifted absorption spectrum enabling efficient *E* to *Z* and *Z* to *E* isomerizations to be initiated by visible light, which facilitates longer-term control of enzyme activity *in vivo* than would be possible with UV irradiation. Computational modeling, guided by local repacking analysis, identified potential allosteric sites in FLuc to aid rational amino acid substitution. Of the seven sites identified, it was found that reversible on/off switching of luciferase activity could be achieved through azobenzene incorporation at Trp417, enabling up to five cycles of photoswitching.

#### 4.4.3 Metal Responsive Regulation

**Figure 22:**
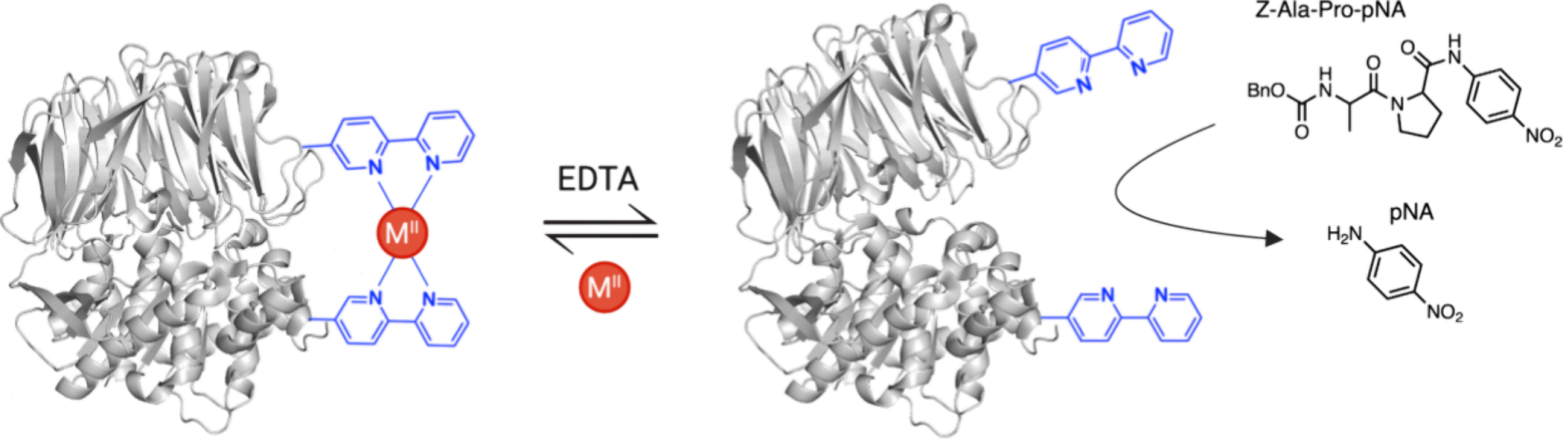
Introduction of a pair of BpyAlas into Pfu POP (PDB: 5T88(458)) enabled inhibition of protease activity when incubated in divalent metal salts. Metal binding of the noncanonical ligands holds POP in a closed inactive conformation, which can be released through chelation of metal ions with EDTA addition, thereby allowing reversible allosteric control of biocatalyst activity. Created with BioRender.com.

In a recent study, Zubi et al. developed a metal-responsive system for protein (in)activation involving the genetic incorporation of two spatially separated bidentate (2,2’-bipyridin-5-yl)alanine (BpyAla) residues to impart conformational control over proteins that do not otherwise exhibit allostery (Figure 22).^457^ The serine protease *Pyrococcus furiosus* (*Pfu*) prolyl oligopeptidase (POP) was selected as a model enzyme due to the dynamic domain opening/closing it undergoes to allow substrate entry and positioning of the catalytic triad, as revealed by MD simulations. To minimize screening efforts, pairs of positions for BpyAla incorporation were identified through parametrized MD simulations, which highlighted residues that were appropriately positioned to chelate metal ions in the closed conformational state to occlude the active site, but not in the open state. When incubated in excess divalent metal salts such as those of Ni(II), Cu(II), Co(II), and Zn(II), protease activity of selected POP variants was almost entirely inhibited as intended, with near quantitative recovery of enzyme activity achieved upon addition of EDTA for up to 24 on/off cycles. Spectroscopic, and computational investigations collectively suggested that the Bpy pairs engage in reversible metal binding to generate the targeted M(II)(BpyAla)_2_ complex, resulting in a conformational change which inhibits catalytic turnover. To illustrate the generality of this approach, the study was extended to develop a metal dependent *Photinus pyralis* luciferase (Pluc), with the best variant showing a 20-fold decreased *V*_max_ in the presence of Ni(II).

## 5 Designing New Catalytic Mechanisms and Functions

A major objective of modern biocatalysis is to broaden scope of chemical transformations beyond those known in nature, by developing enzymes that operate through new catalytic manifolds. To maximize the breadth of chemistry accessible with designed biocatalysts, it is important to be able to introduce a wide variety of functional groups within enzyme active sites. One way to achieve this is to embed new catalytic elements as ncAA side chains. A major advantage of this approach for enzyme design is its versatility. Having established suitable aaRS/tRNA pairs to encode a functional amino acid of interest, this residue can be positioned at various sites in diverse protein scaffolds.^14,27^ Importantly, it has been shown that directed evolution workflows can be adapted to optimize designed enzymes made from an expanded set of amino acid residues.^28,459,460^ As a result of rapid advances in the field, a variety of enzymes have now been developed that use ncAAs as key catalytic motifs.

### 5.1 Metalloenzymes

2,2′-Bipyridine is a widely used bidentate ligand for divalent cations.^461^ Xie et al. expressed T4 lysozyme mutants with BpyAla site-specifically incorporated at several positions on the protein surface.^462^ Incubation of the modified lysozyme variants with CuCl_2_ and subsequent MS and spectroscopic analyses confirmed Cu(II) binding, which was dependent on the BpyAla ligand. Subsequently, BpyAla was used to confer DNA cleavage activity on *E. coli* catabolite activator protein (CAP).^463^ Guided by a crystal structure of dimeric CAP bound to DNA, the surface residue Lys26 was selected for substitution to BpyAla due to its proximity to the DNA–protein interface. When incubated with redox active metal ions such as Fe(II) or Cu(II), a reducing agent and the allosteric activator cAMP, CAP Lys26BpyAla promoted site-selective oxidative cleavage of a bound DNA fragment. Cleavage occurred at either side of a specific nucleobase, suggesting that a freely diffusible oxidizing agent generated at the metal center is responsible for DNA cleavage.

**Figure 23:**
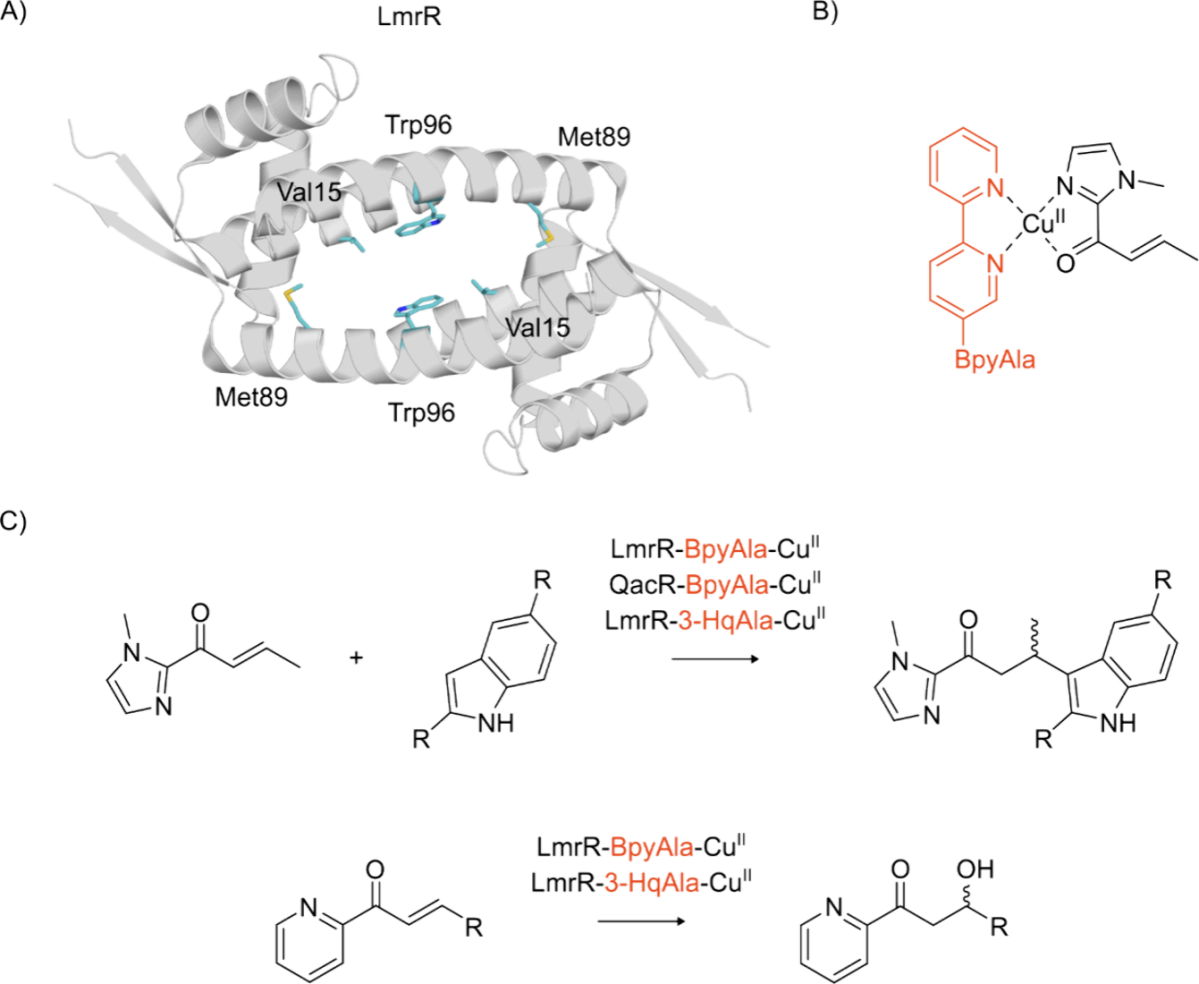
Catalytic metal-coordinating ncAAs. (A) Crystal structure of dimeric LmrR, with the positions Val15, Met89, and Trp96 in the binding pocket shown with blue carbons (PDB: 3F8B(474)). (B) BpyAla-coordinated Cu(II) complex which activates 1-(1-methyl-1*H*-imidazol-2-yl)but-2-en-1-one toward nucleophilic attack. (C) Schemes of vinylogous Friedel–Crafts alkylations (top) and α,β-unsaturated 2-acyl pyridine hydrations (bottom) catalyzed by BpyAla-Cu(II) or 3-HqAla-Cu(II) metalloenzymes.

The transcription factor *Lactoccocal* multidrug resistance Regulator (LmrR) has proven to be a versatile scaffold^464^ for developing metalloenzymes using BpyAla. Drienovská et al. generated copper metalloproteins for enantioselective vinylogous Friedel–Crafts alkylations by incorporating BpyAla into LmrR at position 89 (Figure 23A).^465^ Copper-loaded LmrR Met89BpyAla promoted alkylations of electron-rich indoles with 1-(1-methyl-1H-imidazol-2-yl)but-2-en-1-one (Figure 23B,C) with modest levels of stereocontrol. Reaction selectivity could be further improved through targeted mutagenesis of residues in proximity to the BpyAla ligand. In a later study, a combination of quantum mechanics, docking and MD calculations facilitated the design an LmrR-based metallohydratase.^466^ The designs utilized BpyAla-complexed Cu(II) to catalyze enantioselective hydration of α,β-unsaturated 2-acyl pyridines (Figure 23C), achieving up to 64% *ee*. The same group also demonstrated the binding and stabilization of 2-semiquinone radicals by artificial metalloproteins containing a variety of first row transition metal ions in the LmrR Met89BpyAla template.^467^ This ability to stabilize reactive radical species in the binding pocket of a protein could facilitate the development of artificial enzymes for controlling transformations involving radical intermediates.

In addition to LmrR, several other protein scaffolds have been elaborated into artificial copper metalloenzymes by incorporating BpyAla. For example, a Tyr123BpyAla variant of QacR proved to be an effective biocatalyst for stereocontrolled vinylogous Friedel–Crafts alkylations of indoles with 1-(1-methyl-1H-imidazol-2-yl)but-2-en-1-one (Figure 23C).^468^ Interestingly, this metalloprotein gave the opposite product enantiomers to those formed with LmrR Met89BpyAla. Another study installed BpyAla in a homohexameric acetyltransferase to create a dinuclear copper oxidase.^469^ The innate symmetry in the scaffold allowed the distance between the Cu(II) sites to be varied, inducing electronically coupled dinuclear behavior in variants where the sites were proximal and mononuclear behavior in variants where they were distal. Interestingly, the dinuclear copper variants were found to oxidize ascorbate more rapidly than their mononuclear counterparts.

Beyond BpyAla, other metal-binding ncAAs have been used to generate artificial metalloenzymes. The development of an *Mj*TyrRS pair to incorporate 3-HqAla^150^ enabled production of LmrR variants with 3-HqAla ligands at Val15 or Met89^470^ (Figure 23A) which can be used to chelate Cu(II), Zn(II) and Rh(II) ions. Slow amide bond hydrolysis activity was observed for Zn(II)-containing variants, which did not occur in the presence of the Zn(NO_3_)_2_ salt alone, suggesting that catalysis occurs within the LmrR binding pocket. Cu(II)-loaded variants were found to be competent catalysts for vinylogous Friedel–Crafts alkylations and hydration of α,β-unsaturated carbonyls, albeit with modest selectivities (Figure 23C). More recently, Stein et al. utilized a regioisomer of 3-HqAla, (8-hydroxyquinolin-5-yl)alanine (5-HqAla) to generate a Ru-based metalloenzyme for allylic deamination.^471^ Halotag, a self-labeling protein derived from *Rhodococcus* dehalogenase,^472^ was expressed with 5-HqAla incorporated at positions Phe144, Ala145 or Met175 in the hydrophobic binding cleft. After incubation with [Cp*Ru(MeCN)_3_]PF_6_, all three variants displayed increased deamination activity toward an *O*-allyl carbamate-protected coumarin compared to the WT, with the 5-HqAla175 variant performing up to 9 turnovers. In another study, 3-pyridylalanine (3-PyrAla) was successfully used to transform dimeric bovine pancreatic polypeptide (bPP) into an enantioselective biocatalyst for Diels–Alder cycloadditions and Michael reactions.^473^ A Tyr7 to 3-PyrAla mutation was proposed to create a bidentate metal binding site at the dimer interface. 3-PyrAla-modified bPP was produced by SPPS and incubated with Cu(H_2_O)_6_(NO_3_)_2._ The resulting metalloprotein was found to promote Diels–Alder cycloadditons of α,β-unsaturated carbonyls and cyclopentadiene, and Michael additions of dimethylmalonate and α,β-unsaturated 2-acyl imidazoles, with appreciable levels of stereocontrol (up to 83% and 86% *ee*, respectively).

**Figure 24:**
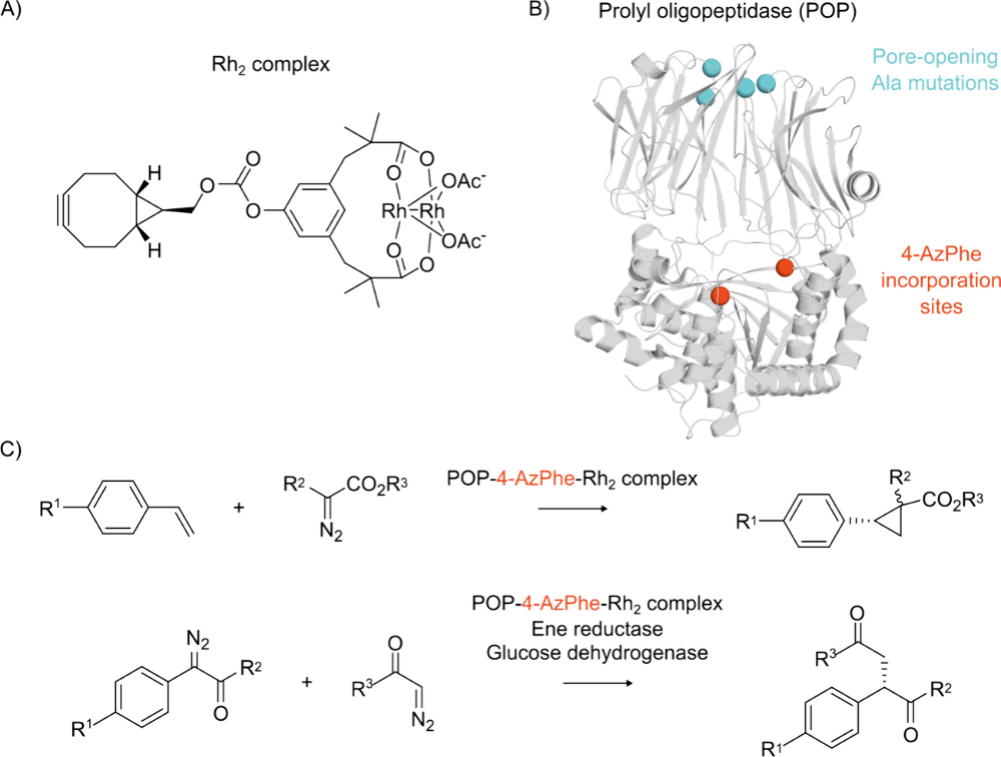
4-AzPhe-anchored metalloenzymes. (A) BCN-Derivatised dirhodium complex. OAc^–^ = acetate anion. (B) Crystal structure of POP, with positions of 4-AzPhe incorporation (orange spheres) and pore-opening alanine mutations (blue spheres) shown (PDB: 5T88(479)). (C) Schemes of styrene cyclopropanations (top) and the diazo cross-coupling cascade (bottom) catalyzed by POP variants containing 4-AzPhe-tethered dirhodium complexes.

Artificial metalloenzymes can also be created by using ncAAs to covalently tether metal complexes to proteins. Biorthogonal SPAACs to 4-AzPhe are well-suited for this purpose.^475^ Yang et al. reported the incorporation of 4-AzPhe into tHisF, a thermostable α,β-barrel protein, and subsequent SPAAC conjugation of metal complexes derivatized with bicyclo[6.1.0]nonyne (BCN).^476^ Tethering of tetracarboxylate dirhodium complexes and Cu- or Mn-terpyridines was demonstrated, with 50–90% conversion to the conjugated species observed. The artificial metalloenzymes were then evaluated for intermolecular cyclopropanation and Si–H insertion activities. Unfortunately, the catalysts gave reduced activity compared with the free metal complex and only minimal selectivity, suggesting the location of the metal complexes near the mouth of the α,β-barrel did not provide a conducive environment for selective catalysis. Greater success was found using POP as a scaffold, chosen for its thermostability and large internal volume suitable for hosting metal complexes.^477^ Initial efforts to conjugate POP variants modified with 4-AzPhe at various positions in the active site with a BCN-derivatized dirhodium paddlewheel complex (Figure 24A) failed, likely due to the enzyme adopting a closed conformation which shields the active site from solvent. Mutation of four residues lining the pore of the β-barrel domain to alanine overcame these issues (Figure 24B), enabling rapid conjugation to form the metalated enzyme. Catalysis of styrene cyclopropanation with a diazo ester (Figure 24C) gave a single diastereomer with 19% conversion and 11% *ee*, which could be further improved to 38% *ee* upon optimization of reaction conditions. Additional improvements were achieved with the introduction of an active site His to coordinate the proximal rhodium center and phenylalanine mutations around the distal rhodium, with the best variant achieving 74% conversion and 92% *ee*. In a subsequent study, a directed evolution platform was established to allow more extensive engineering of artificial metalloenzymes generated using SPAAC.^458^ Starting from the aforementioned quadruple alanine POP mutant, three rounds of mutagenesis and screening generated the improved variant 3-VRVH, which contained 12 mutations and achieved 92% *ee* for 4-methyoxystyrene cyclopropanation, and a significantly higher reaction rate than the optimal variant from the previous study. Directed evolution was also used to optimize the performance of artificial POP metalloenzymes containing dirhodium paddlewheel complexes for efficient cross-coupling of diazoesters.^478^ The most highly evolved metalloenzyme, 5-G, was able to perform an impressive 40,000 turnovers and delivered the cross-coupled products with high levels of stereocontrol (14.9:1 *E*/*Z* selectivity). 5-G was subsequently integrated into a biocatalytic cascade with an alkene reductase and glucose dehydrogenase for NADPH recycling (Figure 24C), leading to the production of derivatized succinate products with up to 61% conversion and > 99% *ee*.

### 5.2 Nucleophilic Catalysis

**Figure 25:**
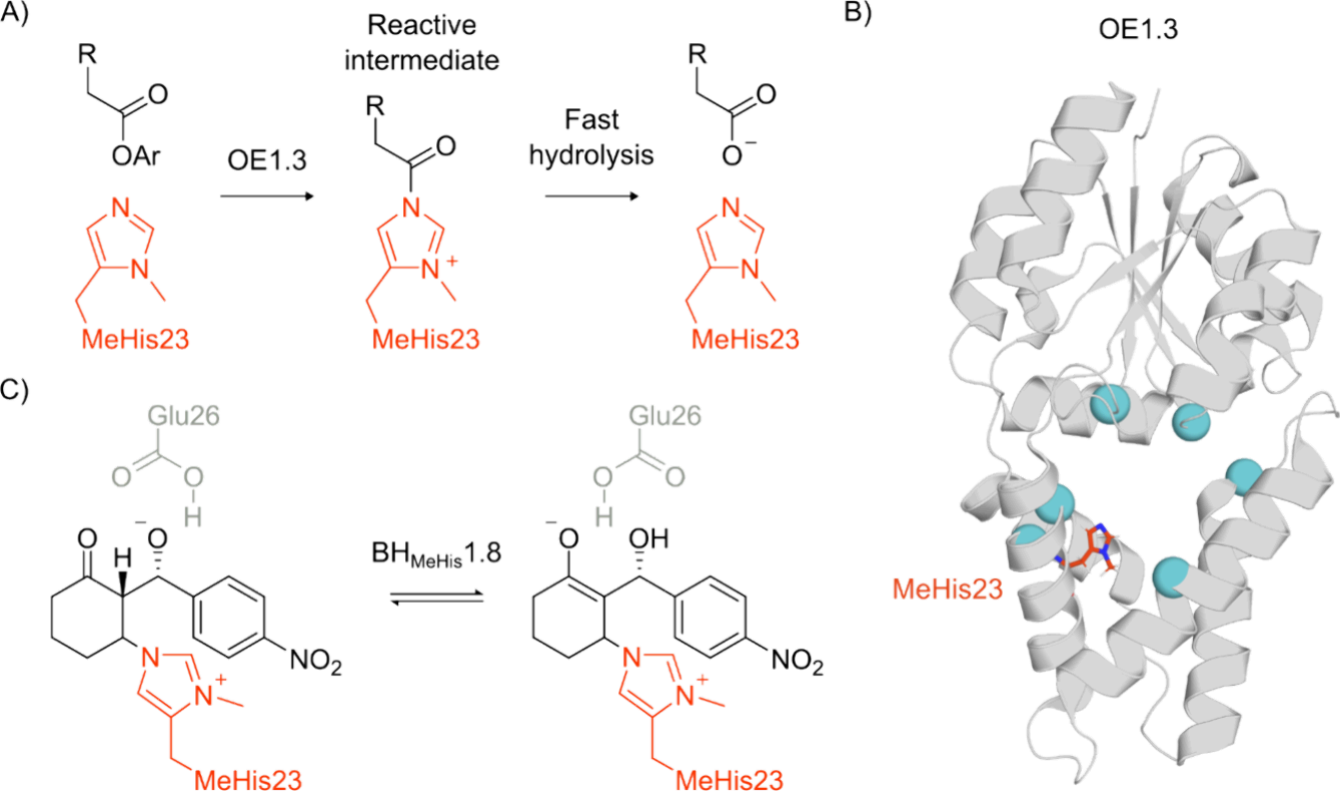
Nucleophilic catalysis utilizing MeHis. (A) Scheme of ester hydrolysis, showing the reactive covalent intermediate formed between the substrate and MeHis23 (orange). (B) Structure of OE1.3, with MeHis23 (orange carbons) and sites of mutations installed during evolution (blue spheres) shown (PDB: 6Q7Q(486)). (C) Scheme highlighting the proton transfer role of Glu26 (gray) in the evolved MBHase BH_MeHis_1.8. Intermediates 2 (left) and 3 (right) are shown, covalently bound to MeHis23 (orange).

Nucleophilic catalysis is a versatile strategy used in chemistry and biology to accelerate diverse transformations. For example, natural hydrolases or acyltransferases often feature an activated Ser or Cys nucleophile embedded within catalytic diads or triads.^480−482^ Efforts to recapitulate these mechanisms in designed enzymes commonly results in proteins where catalysis stalls due to the formation of stable acyl-enzyme intermediates that are resistant to hydrolysis.^483−485^ To overcome these limitations, our lab used GCE to reengineer a computationally designed protein BH32 into an efficient hydrolase.^486^ BH32 was originally designed to catalyze the Morita-Baylis-Hillman (MBH) reaction using a His23 nucleophile,^487^ but was also found to possess promiscuous ester hydrolase activity.^470^ Similar to previously designed hydrolases, BH32 displayed a biphasic reaction profile consistent with rapid acylation of His23 generating a stable acyl-imidazole intermediate that is resistant to hydrolysis. To resolve this bottleneck in catalysis, His23 was replaced by a noncanonical MeHis, which lead to the formation of more reactive acyl-imidazolium intermediates (Figure 25A). Hydrolase activity was subsequently enhanced by directed evolution, affording the enzyme OE1.3 with six mutations (Figure 25B) that is *ca*. 9,000-fold more active than free MeHis in solution, along with an enantioselective hydrolase OE1.4 that contains an additional three mutations (*cf*. OE1.3). MeHis has also been shown to be a valuable catalytic nucleophile for more complex chemical conversions. Installation of MeHis into an engineered BH32 template followed by extensive evolutionary optimization afforded a highly efficient and enantioselective enzyme (BH_MeHis_1.8) for Morita-Baylis-Hillman reactions, which are valuable C–C bond forming processes for which there are no natural enzymes known.^488^ BH_MeHis_1.8 is more than an order of magnitude more active than our earlier BH32.14 enzyme which contains a His23 nucleophile,^489^ and can also promote challenging MBH conversions of electron-rich aromatic aldehydes that were inaccessible with BH32.14. Interestingly, introduction of MeHis led to a dramatically altered mechanistic outcome following evolution, where a key catalytic Arg124 found in BH32.14 was abandoned in favor of a Glu26 residue that mediates a rate-limiting proton transfer step (Figure 25C).

**Figure 26:**
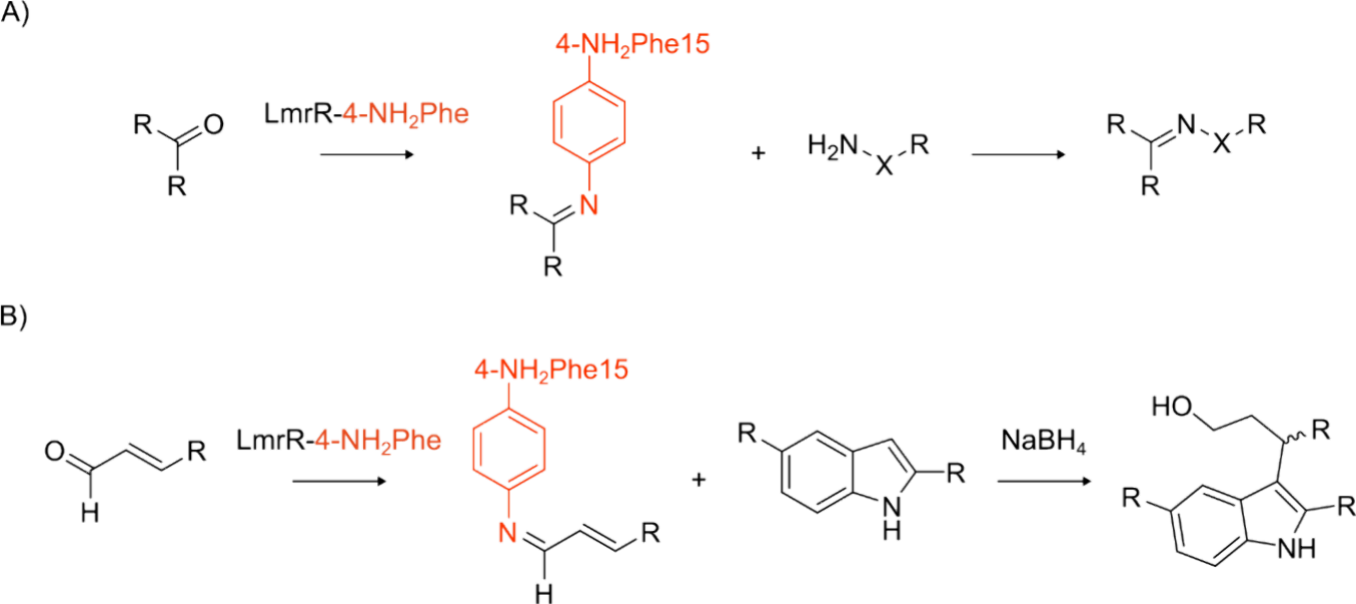
Nucleophilic catalysis utilizing 4-NH_2_Phe. (A) Scheme of hydrazone (X = N) and oxime (X = O) formations catalyzed by 4-NH_2_Phe (orange) incorporated into LmrR, with the covalent adduct formed by the carbonyl substrate and 4-NH_2_Phe15 shown. (B) Scheme of vinylogous Friedel–Crafts alkylations catalyzed by LmrR_V15_4-NH_2_Phe_RGN, with the activated imine intermediate formed between 4-NH_2_Phe15 (orange) and the aldehyde substrate shown. At the end of the reaction time NaBH_4_ is added to reduce the enzymatic product to the corresponding alcohol (right).

Aniline nucleophiles are known to catalyze hydrazone and oxime formations.^490,491^ LmrR was engineered to efficiently catalyze hydrazone formations by embedding an aniline nucleophile.^492^ A two-step protocol involving initial installation of 4-AzPhe by GCE followed by chemical reduction of the aromatic azide was used. The resulting enzyme, LmrR_Val15(4-NH_2_Phe), achieved 72% conversion for a hydrazone formation between 4-methoxybenzaldehyde and a benzoxadiazole, and was also found to catalyze the analogous oxime formation (Figure 26A), with a *k*_cat_/*K*_M_ 37-fold higher than WT LmrR. Hydrazone formation activity was subsequently optimized through directed evolution,^460^ affording triple and quadruple mutants that displayed 57- and 74-fold improved catalytic efficiency respectively compared to the parent enzyme. Mutation of the 4-NH_2_Phe nucleophile to an isosteric Tyr led to dramatic activity reductions in both variants, highlighting the critical role played by the ncAA in accelerating hydrazone formation. The evolved hydrazone-forming enzymes have subsequently been used in combination with alcohol oxidases and carboxylic acid reductases for in vivo biocatalytic cascades.^493^ The reaction scope of 4-NH_2_Phe-containing LmrR variants has been extended to include enantioselective Friedel–Crafts alkylations of indoles (Figure 26B)^459,494^ and enantioselective Michael additions of enals and 2-acylimidazoles.^495,496^ In the latter case, catalysis is dependent upon the synergistic action of the 4-NH_2_Phe nucleophile and a Cu(II)-phenanthroline complex that is sandwiched between two tryptophan residues within the hydrophobic binding pocket, with up to 99% *ee* observed.

More recent studies have disclosed other catalytic ncAAs. Gran-Scheuch et al. reported the synthesis and incorporation of several ncAAs featuring secondary amine motifs using the *Mb*PylRS system.^497^ LmrR containing a pyrrolidine-inspired ncAA at position 15 was found to catalyze Michael addition of nitromethane to cinnamaldehyde with moderate conversions and up to 38% *ee*. Incubation of the enzyme with cinnamaldehyde and NaBH_3_CN gave rise to an MS peak shift consistent with formation of a reduced Schiff-base adduct, implicating ncAA-mediated iminium ion activation of the aldehyde. Another study reported the use of 4-boronophenylalanine (4-BoPhe) installed in engineered LmrR variants to catalyze condensation of α-hydroxyketones with hydroxylamine to form enantioenriched oximes in a kinetic resolution process.^498^ MS and ^11^B NMR studies provided evidence for the catalytic role of 4-BoPhe, which was proposed to form transient boronate adducts with vicinal diol reaction intermediates in a stereoselective manner.

### 5.3 Photocatalysis

**Figure 27:**
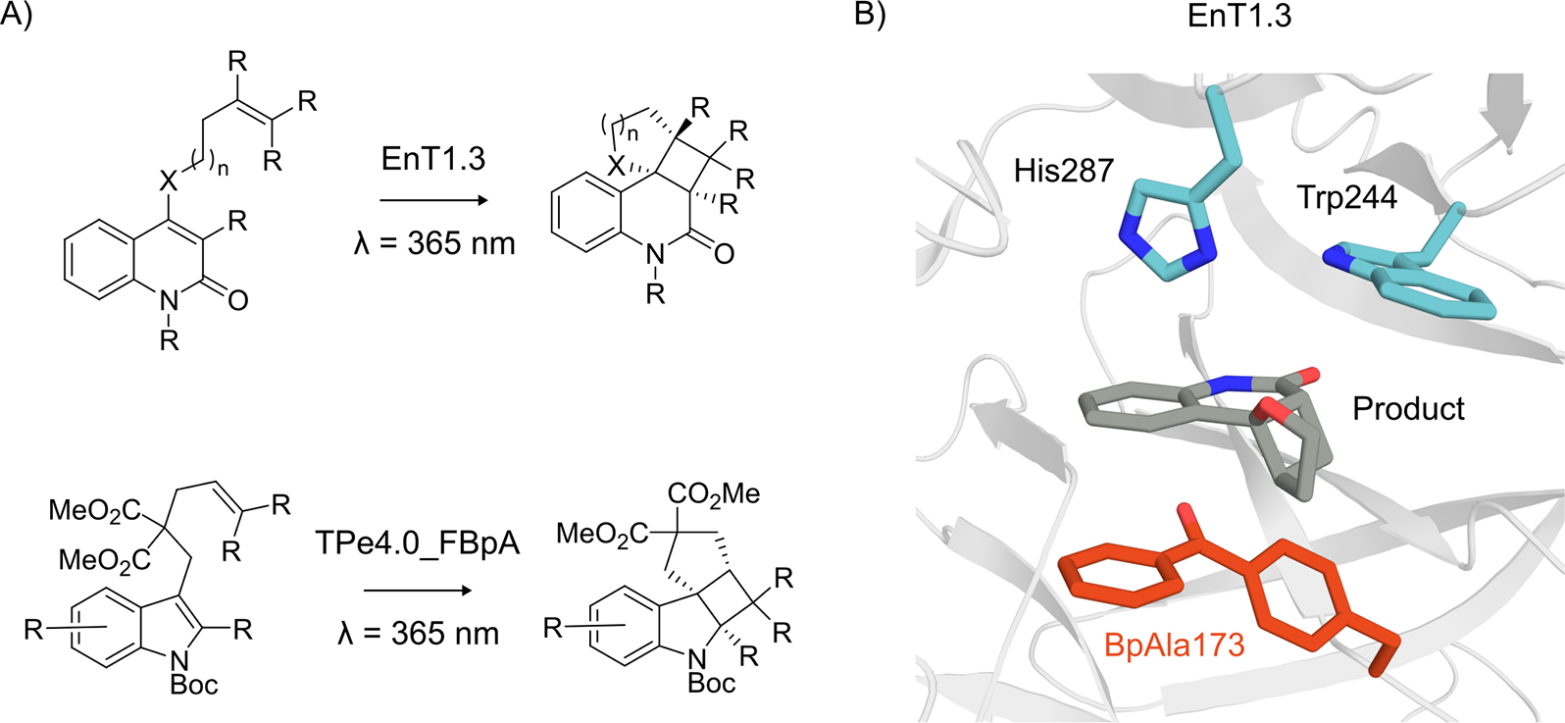
[2 + 2] Photocycloadditions catalyzed by BpAla. (A) Schemes of intramolecular [2 + 2] photocycloadditions of derivatized quinolones (top) and indoles (bottom). X = O or C, *n* = 1 or 2. (B) Crystal structure of EnT1.3 with product (green carbons) bound between BpAla (orange carbons), Trp244, and His287 (blue carbons) (PDB: 7ZP7(28)).

Photons provide a convenient and tuneable source of energy to selectively access reactive excited state intermediates under mild reaction conditions. This use of light energy opens up new modes of reactivity that are challenging to access in the ground state, resulting in the development of diverse synthetic methods for constructing carbon-carbon and carbon-heteroatom bonds.^499,500^ Light-driven reactions can be broadly divided into photoinduced electron transfer (photoredox) processes and triplet energy transfer processes. There are a handful of natural photoenzymes^501−503^ and several engineered photoenzymes^504−506^ that operate *via* photoredox mechanisms. In contrast, there are no natural enzymes known to mediate stereocontrolled transformations through energy transfer mechanisms. To address this limitation, Trimble et al. and Sun et al. independently developed enantioselective enzymes for thermally forbidden [2 + 2] cycloadditions. In the former study, a BpAla triplet sensitizer was incorporated into the active site of a previously designed Diels–Alderase, DA_20_00,^507^ giving rise to a first generation photocatalyst EnT1.0 that could mediate intramolecular [2 + 2] cycloadditions of quinolone substrates (Figure 27A) with modest regio- and enantioselectivity upon irradiation with 365 nm light.^28^ Introduction of five additional mutations *via* directed evolution afforded an optimized photoenzyme EnT1.3 with substantially improved activity, regioselectivity and enantioselectivity (>99% *ee*). This energy transfer photoenzyme can operate efficiently at ambient temperatures and in the presence of oxygen, and can also mediate bimolecular [2 + 2] cycloadditions with high levels of stereocontrol. A product-bound crystal structure of EnT1.3 reveals that the ligand is sandwiched between the BpAla side chain and an active site His (Figure 27B), an arrangement conducive to efficient energy transfer between the photosensitizer and substrate. Sun et al. used a similar approach to develop enantioselective photoenzymes for intramolecular cycloadditions of *N*-substituted indoles using LmrR as a protein scaffold.^508^ In this case, a fluorinated analogue of BpAla (3′-FBpAla) led to improved conversions and selectivities for a number of substrates compared with the parent sensitizer.

**Figure 28:**
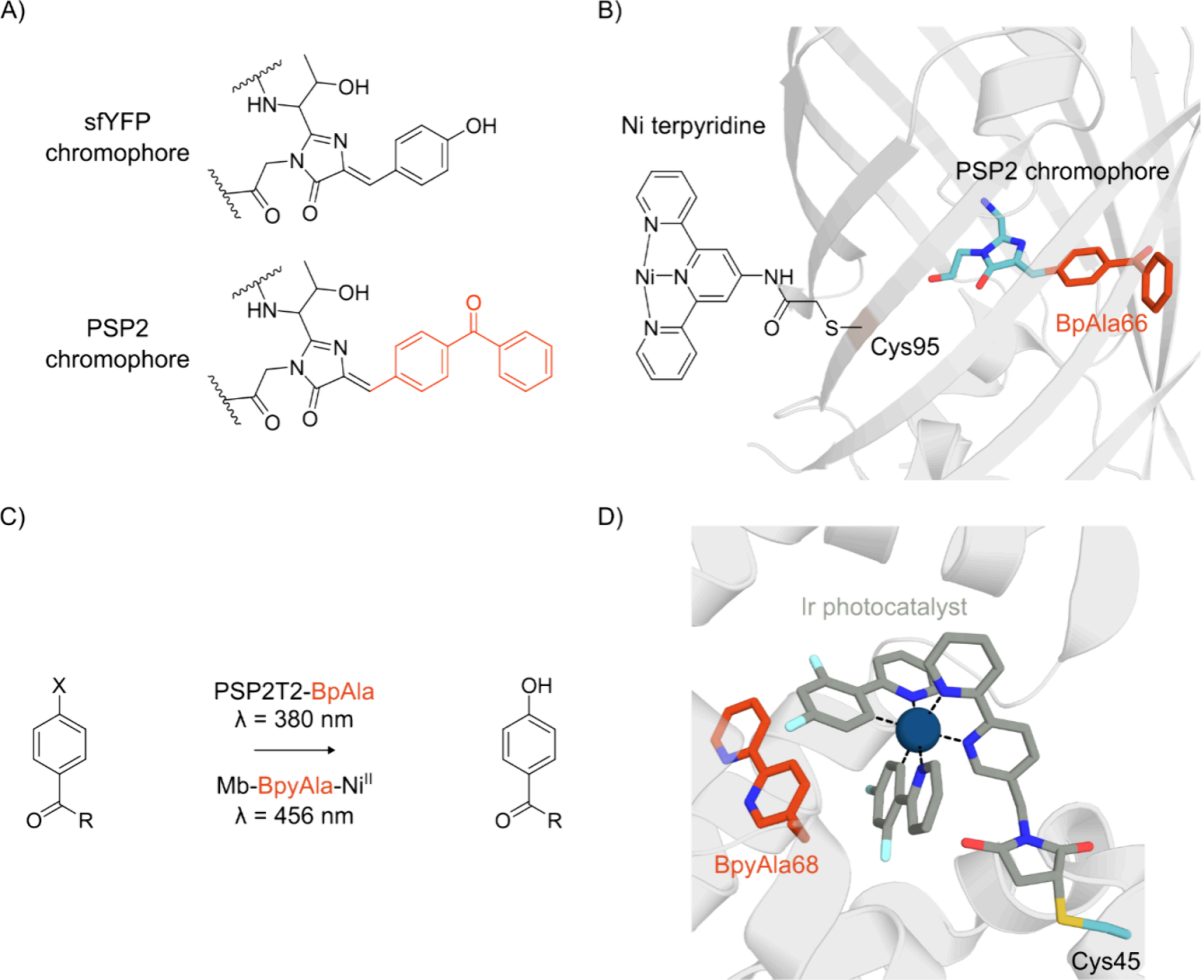
Metal-dependent ncAA-incorporating photoenzymes. (A) Chromophore autocatalytically generated in sfYFP and in PSP2, which incorporates BpAla (orange side chain) at position 66. (B) Structure of PSP2, with a chromophore shown (backbone indicated with gray carbons, BpAla side chain with orange carbons). The Cys95 site of nickel–terpyridine complex ligation is shown in dark gray (PDB: 5YR3(506)). (C) Scheme of dehalogenation reactions catalyzed by BpAla-incorporating PSP2T2 or by BpyAla-incorporating Mb. X = Cl, Br, or I. (D) Structure of Mb incorporating BpyAla (orange carbons) and with an iridium photocatalyst (green carbons) ligated to Cys45 (gray carbons) (PDB: 7YLK(516)).

A large number of chemical transformations can be driven by photoinduced electron transfer (PET) processes.^509,510^ Incorporation of ncAAs into fluorescent proteins has delivered photoredox catalysts by tuning the absorption profiles and redox properties of the chromophore. Liu et al. generated a miniature photocatalytic CO_2_-reducing enzyme by modifying superfolder yellow fluorescent protein (sfYFP),^506^ which features a chromophore generated by autocatalytic cyclization and oxidation of residues Ser65, Tyr66 and Gly67^511^ (Figure 28A, top). Substitution of Tyr66 with BpAla (Figure 28A, bottom) in a His148Glu Phe203Asp mutant of sfYFP generated the photosensitizer protein PSP2.^506^ Photochemical reduction of PSP2 with sacrificial reductants produced super-reducing radicals (PSP2•) which were sufficiently potent to drive CO_2_ reduction by a nickel-terpyridine complex ligated to PSP2 through a Cys residue introduced at position 95 (Figure 28B). Introduction of proton-donating Tyr residues around the nickel complex further improved activity, with the resulting enzyme PSP2T2 exhibiting a CO_2_/CO conversion quantum efficiency of 2.6%. PSP2T2 was also found to catalyze light-driven dehalogenations of simple aryl halides to phenolic products with up to 98% conversion (Figure 28C).^512^

Photoenzymes can also be generated *via* biorthogonal conjugation of organic or metal photocatalysts to 4-AzPhe incorporated into proteins. Gu et al. anchored a BCN-derivatized 9-mesityl-10-methylacridinium cofactor into the active site of POP.^513^ Upon irradiation with 450 nm light, the modified enzyme catalyzed the conversion of thioanisoles to the corresponding sulfoxides, albeit with somewhat lower conversions than the free cofactor. More success was found by anchoring a BCN-derivatized Ru(Bpy)_3_^2+^ complex into POP.^514^ The conjugated enzyme catalyzed photoreductive cyclization of a dienone substrate with 72% conversion, compared to 36% with the free ruthenium complex. Similarly, [2 + 2] photocycloaddition of 4-methoxystyrene and cinnamoyl imidazole proceeded with 81% conversion using the conjugated enzyme but only 25% with the free complex. The same reaction could also be catalyzed using polypyridyl iridium complexes anchored within POP.^515^ Artificial photoenzymes have also been developed by incorporating multiple metal complexes within a protein scaffold. Lee and Song developed an artificial dehalogenase in Mb by introducing a genetically encoded BpyAla to chelate a nickel cofactor and an iridium photocatalyst ligated to a Cys at position 45 (Figure 28D).^516^ The resulting enzyme catalyzed a mixture of light-driven hydrolytic and reductive dehalogenation of 4′-iodoacetophenone (Figure 28C), achieving 86% conversion to the phenolic product and *ca*. 10:1 selectivity over the reduced side product. Reaction selectivity could be further improved through judicious placement of the BpyAla ligated nickel cofactor. In contrast, using the free metal complexes in solution gave substantially lower conversion and chemoselectivity.

## 6 Conclusions and Outlook

The ability to introduce new functional elements into proteins as ncAA side chains has opened a wealth of opportunities in the field of biocatalysis. In this review, we have highlighted how an expanded alphabet of amino acids can be used to study enzyme mechanisms,^255,271,392^ augment biocatalyst properties,^351,362,370,373,339,517^ and create enzymes with new catalytic functions.^450,470^ Moving forward, there are opportunities for new innovations to maximize the impact of ncAAs in biocatalysis research.

First, continued expansion of the genetic code to include a greater repertoire of functional amino acids will be key to unlocking new modes of catalysis within protein active sites. For example, the recent encoding of phosphine-containing ncAAs holds great promise for metalloenzyme design and engineering.^518^ Similarly, a recent article highlights the potential of genetically encoded boronic acids as catalytic motifs.^498^ The introduction of a wider range of ncAAs harboring photoresponsive elements should also allow the development of selective biocatalysts for diverse photochemical reactions. Such photoresponsive ncAAs can also be interfaced with modern structural biology techniques, such as X-ray free electron laser crystallography, to provide new insights into conformational changes that take place during enzyme catalysis.^159^ At present, the application of genetic code reprograming in biocatalysis has largely focused on the introduction of one or multiple copies of a single ncAA. However, with advances in synthetic genomics and the discovery of mutually orthogonal aaRS-tRNA pairs it is now possible to selectively introduce multiple ncAAs into proteins.^116,117,519−523^ In principle, these advances will greatly expand the range of active site arrangements accessible in proteins, which should allow the development of increasingly sophisticated catalytic mechanisms.

Second, to fully capitalize on our ability to embed new functional motifs into proteins, we require accelerated enzyme engineering pipelines to deliver ncAA-containing biocatalysts with the properties required for target applications. For example, the design and evolution of new enzymes that use ncAAs as key functional elements typically takes several years with existing workflows. One approach to speed up biocatalyst development is to integrate ncAA mutagenesis with ultrahigh throughput screening methods (e.g., fluorescence-activated droplet sorting^524^), which allow greater exploration of protein sequence space to accelerate directed evolution campaigns. To complement experimental engineering techniques, the latest deep-learning methods for protein design^6,7,525−527^ and structure prediction^528^ also hold enormous promise. These methods have allowed the accurate design of proteins that bind metal ions, small molecules and peptides, and have recently been used to design new and improved enzymes.^8^ A crucial next step is to integrate ncAAs into these deep-learning frameworks, which should enable the rapid design of ncAA-containing enzymes with a high degree of accuracy.

Finally, in the coming years it is essential to transition ncAA-containing enzymes from academic laboratories into large-scale biocatalytic applications. Current barriers to translation include the limited efficiency of orthogonal translation components and suboptimal performance of common production strains for genetic code reprogramming applications. These limitations lead to reduced titers of ncAA-containing proteins and the requirement for large excesses of ncAAs supplemented to the culture media, which results in prohibitively high production costs. To overcome these challenges, more efficient orthogonal translation components are required that can operate effectively at low ncAA concentrations. The development of such systems should be achievable through multiple rounds of laboratory evolution to progressively improve performance, or through advanced techniques such as multiplex automated genome engineering,^122^ phage-assisted continuous evolution,^120^ tRNA display^119^ and computational approaches.^529,530^ A recent study has also shown that exploration of a wider range of PylRS homologues can dramatically improve the efficiency of encoding catalytically important ncAAs.^531^ To further improve protein titers, engineered or synthetic production strains can be used that have been specifically tailored for efficient ncAA incorporation.^532−536^ Another attractive option to reduce the costs of producing ncAA-containing proteins is developing engineered hosts that contain the necessary biosynthetic machinery to produce target ncAAs *in vivo*,^330,537−539^ thus avoiding the need to supply expensive ncAAs to the culture media.

For the reasons outlined above, we are optimistic that genetic code reprogramming methodologies will become increasingly important tools in enzymology and biocatalysis in the coming years. By overcoming the constraints of the genetic code, enzyme designers and engineers can now begin to tackle a vast array of chemical transformations that were previously thought inaccessible to biocatalysis.

## Abbreviations

aaRS: Aminoacyl tRNA synthetase
AFM: Atomic force microscopy
AKR: Aldehyde ketone reductase
APX2: Ascorbate peroxidase, engineered
AsSec: *Aeromonas salmonicida* selenocysteine synthase
AzoR: Azoreductase
BCN: Bicyclo[6.1.0]nonyne
BODIPY: Boron-dipyrromethene
bPP: Bovine pancreatic polypeptide
cAA: Canonical amino acid
CalB: *Candida antartica* lipase B
CAP: Catabolite activator protein
CAT: Chloramphenicol acetyltransferase
C*c*P: Cytochrome *c* peroxidase
CDO: Cysteine dioxygenase
CFE: Cell-free expression
ChPylRS: Chimeric pyrrolysyl tRNA synthetase
CuAAC: Copper-catalyzed azide–alkyne coupling
CYP: Cytochrome P450
DADP: Diacetyl deuteroporphyrin
ddNTP: 2′,3′-dideoxynucleotide triphosphate
DEER: Double electron–electron resonance
DET: Direct electron transfer
DKR: Diketoreductase
DQF-COSY: Double quantum filtered correlation spectroscopy
*dr*: Diastereomeric ratio
*Ec*LeuRS: *Escherichia coli* leucyl tRNA synthetase
EDA: Ethyl diazoacetate
EDTA: Ethylenediaminetetraacetic acid
*ee*: Enantiomeric excess
ELP: Elastin-like polypeptide
EPL: Expressed protein ligation
EPR: Electron paramagnetic resonance
FDH: Formate dehydrogenase
FLuc: Firefly luciferase
GCE: Genetic code expansion
GFP: Green fluorescent protein
GST: Glutathione *S*-transferase
HAT: Hydrogen atom transfer
HCO: Heme copper oxidase
HRP: Horseradish peroxidase
HSQC: Heteronuclear single quantum coherence
IEDDA: Inverse-electron-demand Diels–Alder
ImGPS: Imidazole glycerol phosphate synthase
IR: Infrared spectroscopy
KlenTaq: DNA polymerase I from *Thermus aquaticus*
KSI: Ketosteroid isomerase
LaL: Lysozyme from bacteriophage λ
LmrR: *Lactoccocal* multidrug resistance regulator
ManA: Mannose-6-phosphate isomerase
Mb: Myoglobin
MBH: Morita–Baylis–Hillman
*Mb*PylCKRS: *Methanosarcina barkeri* pyrrolysyl photocaged lysine tRNA synthetase
*Mb*PylRS: *Methanosarcina barkeri* pyrrolysyl tRNA synthetase
MD: Molecular dynamics
MLDH: Malate dehydrogenase
MNDH: Mannitol dehydrogenase
mDHFR: Murine dihydrofolate reductase
*Mj*TyrRS: *Methanocaldococcus jannaschii* tyrosyl tRNA synthetase
*Mm*PylRS: *Methanosarcina mazeii* pyrrolysyl tRNA synthetase
*Mm*SepRS: *Methanococcus maripaludis* phosphoseryl-tRNA synthetase
MS: Mass spectrometry
MTG: Microbial transglutaminase
mtRNAP: Mitochondrial RNA polymerase
ncAA: Noncanonical amino acid
NCL: Native chemical ligation
NiSOD: Nickel-dependent superoxide dismutase
NMR: Nuclear magnetic resonance
PCR: Polymerase chain reaction
pdCpA: 5′-phospho-2′-deoxyribocytidylriboadenosine
PEG: Polyethylene glycol
PET: Photoinduced electron transfer
PLA: Phospholipase A_2_
*P*Luc: *Photinus pyralis* luciferase
POP: Prolyl oligopeptidase
POR: Protochlorophyllide oxidoreductase
PRMT1: Protein arginine methyltransferase 1
PTE: Phosphotriesterase
PTM: Post-translational modification
*R*Luc: *Renilla* luciferase
RNase: Ribonuclease
RNR: Ribonucleotide reductase
ROS: Reactive oxygen species
SAD: Single-wavelength anomalous diffraction
SAM: *S*-adenosylmethionine
SCS: Stop codon suppression
sfYFP: Superfolder yellow fluorescent protein
SHC: Squalene-hopene cyclase
SPAAC: Strain-promoted azide–alkyne coupling
SPI: Selective pressure incorporation
SPPS: Solid-phase peptide synthesis
sTCO: Strained *trans*-cyclooctene
T7RNAP: T7 RNA polymerase
tHisF: *Thermotoga maritima* synthase subunit of ImGPS
TMS: Trimethylsilyl
TOCSY: Total correlation spectroscopy
TR: Thioredoxin
TrpOx: Tryptophan oxidase
tsCA: Thermostable carbonic anhydrase II
TTL: *Thermoanaerobacter thermohydrosulfuricus* lipase
TTN: Total turnover number
*Tv*NiR: *Thioalkalivibrio nitratireducens* cytochrome *c* nitrite reductase
VHR: Vaccinia H1-related
VSE: Vibrational Stark effect
WT: Wild type
XANES: X-ray absorption near edge structure
XFEL: X-ray free-electron laser
ZFN: Zinc finger nuclease
